# Optimization of 4‐Amino‐2‐Pyridone Inhibitors of Proprotein Convertase Subtilisin/Kexin Type 9: Integrating Structure–Activity and Structure–Metabolism Relationships

**DOI:** 10.1002/cmdc.202500651

**Published:** 2025-11-28

**Authors:** Lisa Giannessi, Maria Giovanna Lupo, Martina Ugolotti, Bianca Papotti, Beatrice Mattina, Maria Grazia Martina, Anna Demurtas, Cristina Padula, Sara Nicoli, Marco Crescenzio, Nicola Ferri, Francesca Zimetti, Marco Radi

**Affiliations:** ^1^ Dipartimento di Scienze degli Alimenti e del Farmaco (DipALIFAR) Università degli Studi di Parma Viale delle Scienze 27/A 43124 Parma Italy; ^2^ Department of Medicine University of Padova 35128 Padova Italy

**Keywords:** green chemistry, proprotein convertase subtilisin/kexin type 9, PCSK9, pyridones, small molecules, structure–metabolism relationship

## Abstract

Proprotein convertase subtilisin/kexin type 9 (PCSK9) is a key drug target for the treatment of different hypercholesterolemia‐related diseases. A new class of small‐molecule inhibitors of PCSK9 transcription, characterized by a 4‐amino‐2‐pyridone scaffold, has been recently identified by our research group. Among them, the early lead compound **5c** shows high in vitro potency and favorable in vivo tolerability. However, given the suboptimal in vitro metabolic stability of **5c**, its optimization is reported herein by modification of the predicted metabolic soft spots through chemistry‐driven late‐stage functionalization (LSF) strategies. Microsomal stability on the newly synthesized derivatives allows drawing structure–metabolism relationships (SMRs) that, coupled with a thorough pharmacological investigation on HepG2 cells, leads to the identification of novel C3‐ and dual C3/NHC4‐functionalized pyridones with improved stability and superior pharmacological profiles. Notably, compounds **6b**, **7**, and **18a** emerge as the best candidates, demonstrating markedly improved metabolic stability/PCSK9 IC_50_ ratio and comparable or lower cytotoxicity with respect to the parent compound **5c**. These findings underscore the value of LSF strategies in generating optimized analogs of **5c** with strong potential for further preclinical development.

## Graphical abstract

Green chemistry‐based late‐stage functionalization (LSF) of the anti‐proprotein convertase subtilisin/kexin type 9 (PCSK9) early lead compound **5c** led to optimized analogs with improved metabolic stability/PCSK9 IC_50_ ratio.

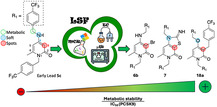

## 1 Introduction

**Figure 1:**
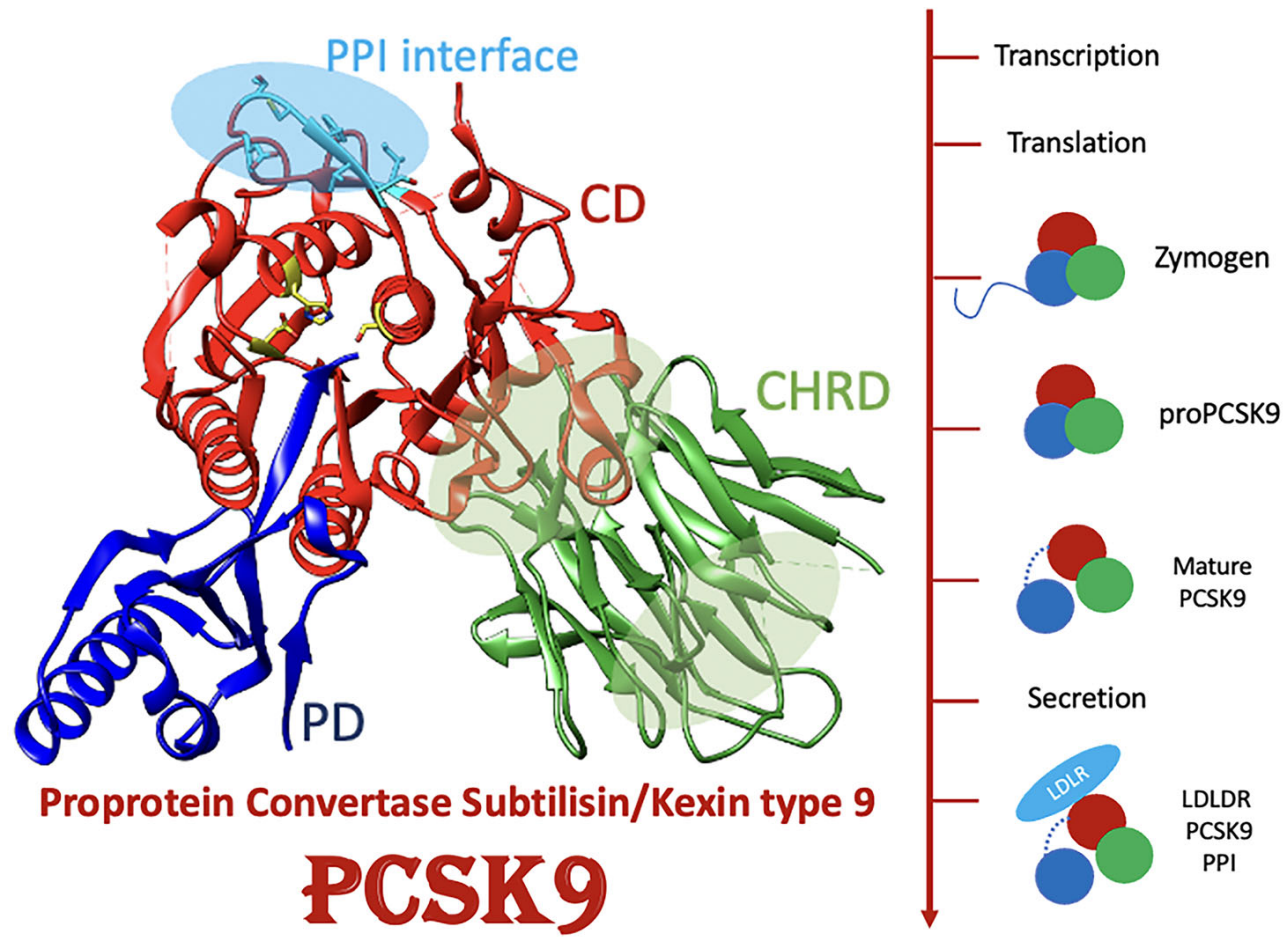
Representation of PCSK9 structure, domains, and biosynthesis. The three main domains are highlighted: prodomain (PD), catalytic domain (CD), and cysteine and histidine‐rich C‐terminal domain (CHRD). PCSK9 is initially synthesized as a zymogen and matures through self‐cleavage from proPCSK9 to PCSK9, after which it can be secreted. Mature PCSK9 interacts with the low‐density lipoprotein receptor (LDLR), promoting its lysosomal degradation and thereby lowering the LDL‐cholesterol clearance.

Proprotein convertase subtilisin/kexin Type 9 (PCSK9) is a serine protease and the ninth member of the proprotein convertase family (**Figure** **1**).^[^ ^1^ ^]^ From its discovery in 2003 as a key player in cholesterol homeostasis, PCSK9 has quickly become a target for drug development. Two monoclonal antibodies, alirocumab and evolocumab, as well as the small interfering RNA Inclisiran, which inhibit PCSK9, have been approved for the treatment of hypercholesterolemia‐related cardiovascular diseases (CVD).^[^ ^2^ ^]^ Beyond this established role in CVD, PCSK9 is being investigated for its involvement in a range of other pathological conditions, including cancer, infectious diseases, and neurodegenerative disorders. In cancer research, PCSK9 could promote tumor growth through lipid metabolism alterations and immune system modulation. Furthermore, recent studies have highlighted that it may also promote metastatic processes. In this context, PCSK9 inhibitors have shown promising potential to enhance immune responses, especially when used in combination with immune checkpoint inhibitors.^[^ ^3^, ^4^, ^5^, ^6^, ^7^, ^8^ ^]^ Altered levels of PCSK9 have also been observed in patients with viral infections, and in vitro studies suggest that PCSK9 inhibitors could provide protective benefits against diseases like dengue and SARS‐CoV‐2 infections, sepsis, and septic shock.^[^ ^9^, ^10^, ^11^, ^12^, ^13^ ^]^ Emerging evidence also points to PCSK9 as a potential biomarker and therapeutic target in neurodegenerative diseases. PCSK9 has been studied in relation to Alzheimer's disease (AD) pathogenesis.^[^ ^14^ ^,^ ^15^ ^]^ Initial findings suggest that PCSK9 promotes neuronal death and exacerbates beta‐amyloid (Aβ)‐induced neurotoxicity. The neurotoxic effect occurs through the activation of caspases and/or suppression of ApoER2 expression.^[^ ^16^ ^,^ ^17^ ^]^ In addition, silencing PCSK9 in AD mice models led to improved memory functions, reduced Aβ plaque deposition and attenuated neuroinflammation.^[^ ^18^ ^]^ Moreover, elevated PCSK9 levels have been detected in the cerebrospinal fluid of AD patients.^[^ ^19^ ^]^ However, the exact relationship between PCSK9 and AD remains unclear and requires further investigation. Together, these findings underscore PCSK9's therapeutic versatility across a wide range of conditions and emphasize the need for the development of novel, affordable, and orally administrable PCSK9 inhibitors, which are yet not available on the market, representing a significant and promising drug discovery opportunity.

**Figure 2:**
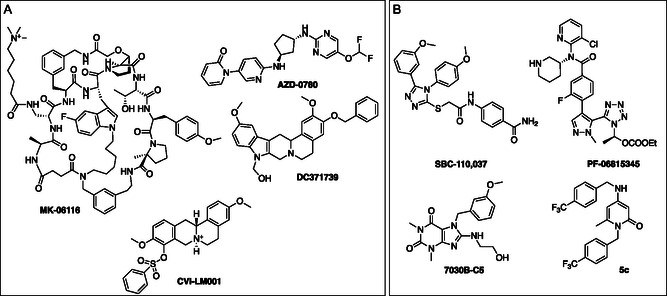
Representative examples of small‐molecules PCSK9i. A) Compounds in the clinical phase of experimentation; B) Compounds in the preclinical phase of experimentation.^[^ ^20^ ^]^

Despite initial challenges in identifying small‐molecule PCSK9 inhibitors (PCSK9i), recent years have seen significant progress, with numerous inhibitors being designed and developed by pharmaceutical companies and academic research groups (**Figure** **2**A,B). These efforts validated the druggability of PCSK9 with small molecules and culminated with the development of four inhibitors (MK‐0616, AZD‐0780, DC371739, and CVI‐LM001) currently in clinical trials for CVD.^[^ ^20^ ^]^ Our research group has recently identified the 4‐amino‐2‐pyridone class of small‐molecule PCSK9i, with compound **5c** emerging as an early lead (Figure 2B).^[^ ^21^ ^]^ Compound **5c** effectively reduced PCSK9 secretion from HepG2 cells at a 5 µM concentration and was well tolerated in mice following seven days of daily subcutaneous administration. However, the in vivo experiments did not demonstrate a significant reduction in circulating PCSK9 levels, indicating room for improvement.

In the present study, we describe the optimization of the early lead **5c** through sustainable late‐stage functionalization (LSF) that generated a series of new point‐modified derivatives with improved metabolic stability/PCSK9 IC_50_ ratio, which is a key requirement to overcome the poor in vivo performance of **5c** and select the most promising derivatives for future efficacy testing in mice.

## 2 Results and Discussion

Achieving an optimal balance between potency and parameters influencing pharmacokinetic (PK) behavior, such as metabolic stability, represents a major challenge in drug development.^[^ ^22^ ^,^ ^23^ ^]^ This issue became evident when the early lead compound **5c** was evaluated in vitro for metabolic stability using a microsomal assay: as shown in Figure 4, **5c** revealed a suboptimal metabolic stability that could be responsible for the lack of effect on circulating PCSK9 observed in mice and needs to be addressed by a focused lead optimization campaign. However, since the specific molecular target of this molecule is still not known, its optimization cannot be assisted by a target‐based analysis. As a result, LSF was identified as the best strategy to perform small chemoselective functionalizations aimed at improving the compounds’ properties with a single synthetic step, while retaining the functional effect on PCSK9.^[^ ^24^ ^,^ ^25^ ^]^

**Figure 3:**
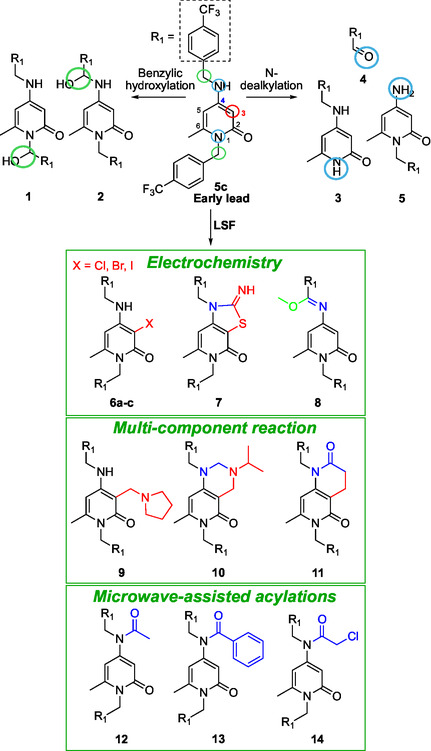
Metabolites of compound **5c** predicted by BioTransformer 3.0 and collection of LSF derivatives prepared by exploiting different green chemistry tools: electrochemistry, MCR, and μW‐assisted acylation.

**Scheme 1:**
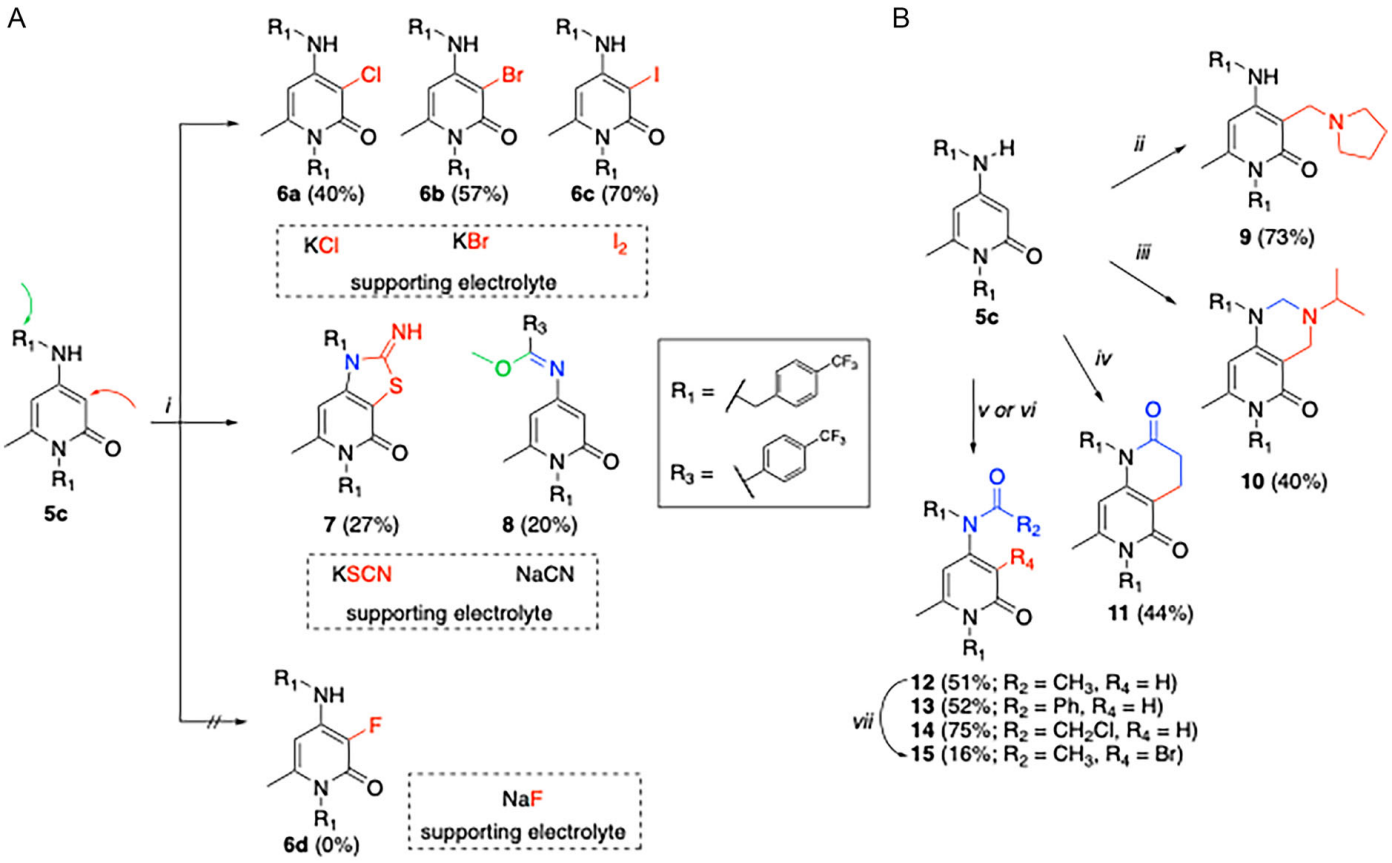
Reagents and conditions: A) i) **5c** (1.0 eq; 0.1 mmol scale), Electrasyn 2.0 apparatus, undivided cell, room temperature, graphite electrodes, DDQ (0.20 equiv.) electrocatalyst, proper supporting electrolyte (2.0 equiv.), and MeOH (4.0 mL). Constant current of 10 mA (for products **6a–c** and **7**) and of 1.5 mA for compound **8**, until complete conversion of the starting material (2–8 F mol^–^^1^). B) ii) Paraformaldehyde (1.5 equiv.), pyrrolidine (2.0 equiv.), CH_3_CN/H_2_O 1/1 (2.0 mL), μW, sealed tube, 40 min, 80 °C; iii) paraformaldehyde (4 equiv.), isopropyl amine (2.0 equiv.), CH_3_CN/H_2_O 1/1 (2.0 mL), μW, open vessel, 20 min, 80 °C; iv) paraformaldehyde (3.5 equiv.), Meldrum acid (4.0 equiv.), CH_3_CN/H_2_O 1 mL/1 mL, 80 °C, 24 h, conventional heating in flask; v) For **12** and **13**, acyl chloride (2.0 equiv.), Et_3_N (2.0 equiv.), dry DCM, μW, 40 °C, 40 min., sealed tube; vi) for **14**, chloroacetyl chloride (4.0 equiv.), Et_3_N (4.0 equiv.), dry DCM, reflux overnight; vii) **12** (1.0 eq; 0.1 mmol scale), Electrasyn 2.0 apparatus, undivided cell, room temperature, graphite electrodes, DDQ (0.20 equiv.) electrocatalyst, proper supporting electrolyte (2.0 equiv.), and MeOH (4.0 mL). Constant current of 10 mA, until complete conversion of the starting material (4 F mol^–^^1^).

Specific sites for point modifications of **5c** were chosen based on two key factors: the known chemical reactivity of the 4‐amino‐2‐pyridone scaffold, and the predicted metabolic soft spots of **5c** (**Figure** **3**). To identify potential metabolic vulnerabilities, the BioTransformer 3.0 platform was used as a predictive tool for metabolic transformations.^[^ ^26^ ^]^ This analysis highlighted benzylic hydroxylation products (**1**–**2**) and N‐dealkylation derivatives (**3**–**5**) as the primary metabolites of **5c**. These predictions suggested the functionalization sites for LSF transformations that may lead to **5c** derivatives with improved metabolic stability. The C3 position and the amino group at C4 were chosen as functionalization sites due to their established reactivity and strategic placement near metabolically vulnerable regions of the molecule. Although the biological significance of the 2‐pyridone scaffold is well established, its chemical reactivity has been relatively underexplored. To address this gap, we have recently published the development of different green chemistry protocols for the LSF of the 4‐amino‐2‐pyridone scaffold.^[^ ^27^ ^]^ These approaches included electrochemical LSF (e‐LSF) reactions, multicomponent LSF (MCR‐LSF) protocols and microwave‐assisted acylation that have been employed for the synthesis of all derivatives herein reported (**Scheme** **1**A,B). All methods were designed to be chemoselective and environmentally sustainable. N‐acylation of the amino group at C4 (**12–13**) was hypothesized to introduce steric hindrance, protecting both the NH group at C4 and the nearby benzylic position. Additionally, the acyl group's mesomeric and electron‐withdrawing properties were expected to decrease electron density in adjacent positions, possibly improving metabolic stability. Similarly, the addition of electron‐withdrawing groups at the C3 position (**6a–c**) aimed to lower the compound's susceptibility to oxidative metabolism by reducing electron density across the heterocyclic framework. Finally, the contemporary functionalization of C3 and NH in C4 (**7**, **10,** and **11**), the benzylic position in C4 (**8**) or the C3 position (**9**), might protect these positions from metabolic reactions.

### 2.1 Chemistry

The e‐LSF protocols enabled the sustainable synthesis of point‐modified derivatives of **5c** at all three key sites of interest (Scheme 1A). Different products were selectively obtained by varying the supporting electrolyte. The C3‐halogenated derivatives **6a–c** were synthesized using appropriate halide inorganic salts (KBr and KCl) or molecular iodine (I_2_). In the presence of KSCN, compound **7** was formed, while using NaCN, **5c** underwent benzylic methoxylation followed by further oxidation to yield the corresponding enamine **8**. Fluorination at the C3 position of the 4‐amino‐2‐pyridone scaffold was also explored as a strategy to enhance the metabolic stability of compound **5c**. Accordingly, electrochemical fluorination of **5c** was attempted under the standard e‐LSF conditions, using as fluorinating reagent NaF (Scheme 1A), but no reaction was observed. In addition to the inorganic fluorinating reagent NaF, organic fluorinating reagents (Et_3_N·3HF and TBAF·3H_2_O, entries 1–4, Table S3, Supporting Information) were evaluated, while DME and CH_3_CN were also attempted as alternative solvents (entries 1–3, Table S3, Supporting Information) to MeOH. To address possible anode passivation, frequently reported as a limiting factor in these reactions, electrode polarity was alternated (entry 4, Table S3, Supporting Information). Despite extensive optimization, including variations in solvent, current density, applied potential, and electrode material, no conversion to the desired fluorinated derivative **6d** was achieved. To investigate the effect of dual functionalization on the metabolic stability of **5c**, the acetylated derivative **12** was brominated using the established e‐LSF protocol, successfully producing the corresponding product **15**.

The application of a MCR‐LSF protocol on compound **5c** allowed the chemoselective functionalization on C3 or the dual functionalization on C3 and NHC4 (Scheme 1B). Specifically, **5c** was reacted with formaldehyde and different nucleophiles in three‐component reactions, yielding compounds **9–11**. Using secondary amines (e.g., pyrrolidine) the C3 modified product **9** was obtained, while primary amines (e.g., isopropyl amine) afforded the bicyclic compound **10**, an example of functionalization on two possible metabolic soft‐spots (C3 and NHC4). Also, C‐nucleophiles were investigated, requiring a specific optimization of reaction conditions (see Table S1, Supporting Information). Under optimized conditions, Meldrum's acid afforded the bicyclic derivative **11**, as further example of dual‐site functionalization. To obtain derivatives with a single modification at the NH group in the C4 position of **5c**, acylations with opportune acyl chlorides were performed to generate compounds **12–14** (Scheme 1B). The microwave‐assisted protocol previously optimized for the synthesis of **12** was also employed to prepare **13** in acceptable yields (52%). However, when the same protocol was used to synthesize **14**, the reaction was unsuccessful, likely due to the high reactivity of the chloroacetyl chloride reagent. Alternatively, standard acylation conditions conducted in a flask successfully produced **14** with a good yield (75%).

**Scheme 2:**
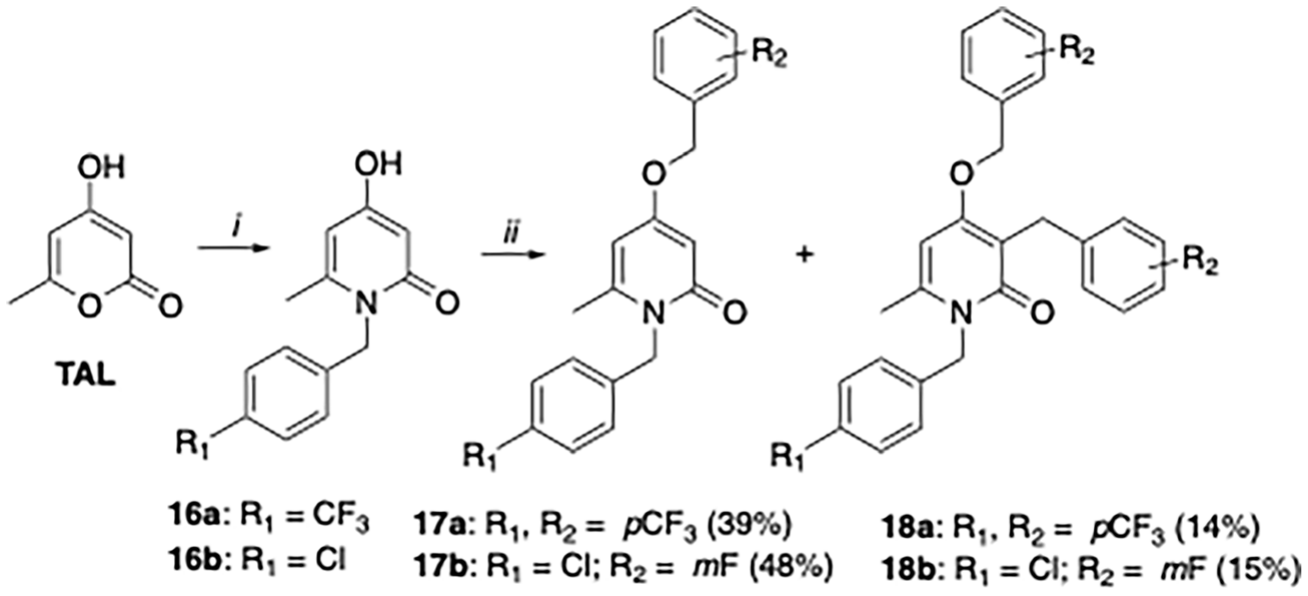
General procedure for the synthesis of 4‐alkoxy‐2‐pyridone compounds. Reaction conditions: i) substituted benzylamine (1.0 equiv.), water reflux 2–4 h; ii) substituted benzyl bromide (1.2 equiv.), K_2_CO_3_ (3.0 equiv.), DMF, r.t., overnight.

In addition, to further expand the structure–metabolism relationships (SMRs) of the 2‐pyridone scaffold, a point modification of compound **5c** was performed through an isosteric replacement of the C4 amine with an oxygen atom, since the resulting ether derivative **17a** should be more resistant to dealkylation‐based metabolic transformations. The synthesis of **17a** required a two‐step protocol (**Scheme** **2**): first, intermediate **16a** was prepared by reacting triacetic acid lactone (TAL) with 4‐(trifluoromethyl)benzylamine in refluxing water following a previously established method.^[^ ^21^ ^]^ Next, the resulting **16a** was reacted with 4‐(trifluoromethyl)benzylbromide to give the desired compound **17a**. Surprisingly, the latter compound was obtained in mixture with the double functionalized derivative **18a**, which represents another dual modified derivative on the hypothetical metabolic soft spots in NHC4 and C3. To verify if this unexpected reactivity was due to the specific functionalization of the compounds, we applied the same protocol to the synthesis of **16b**, which also gave a mixture of two compounds (**17b**, **18b**) after the second step.

**Figure 4:**
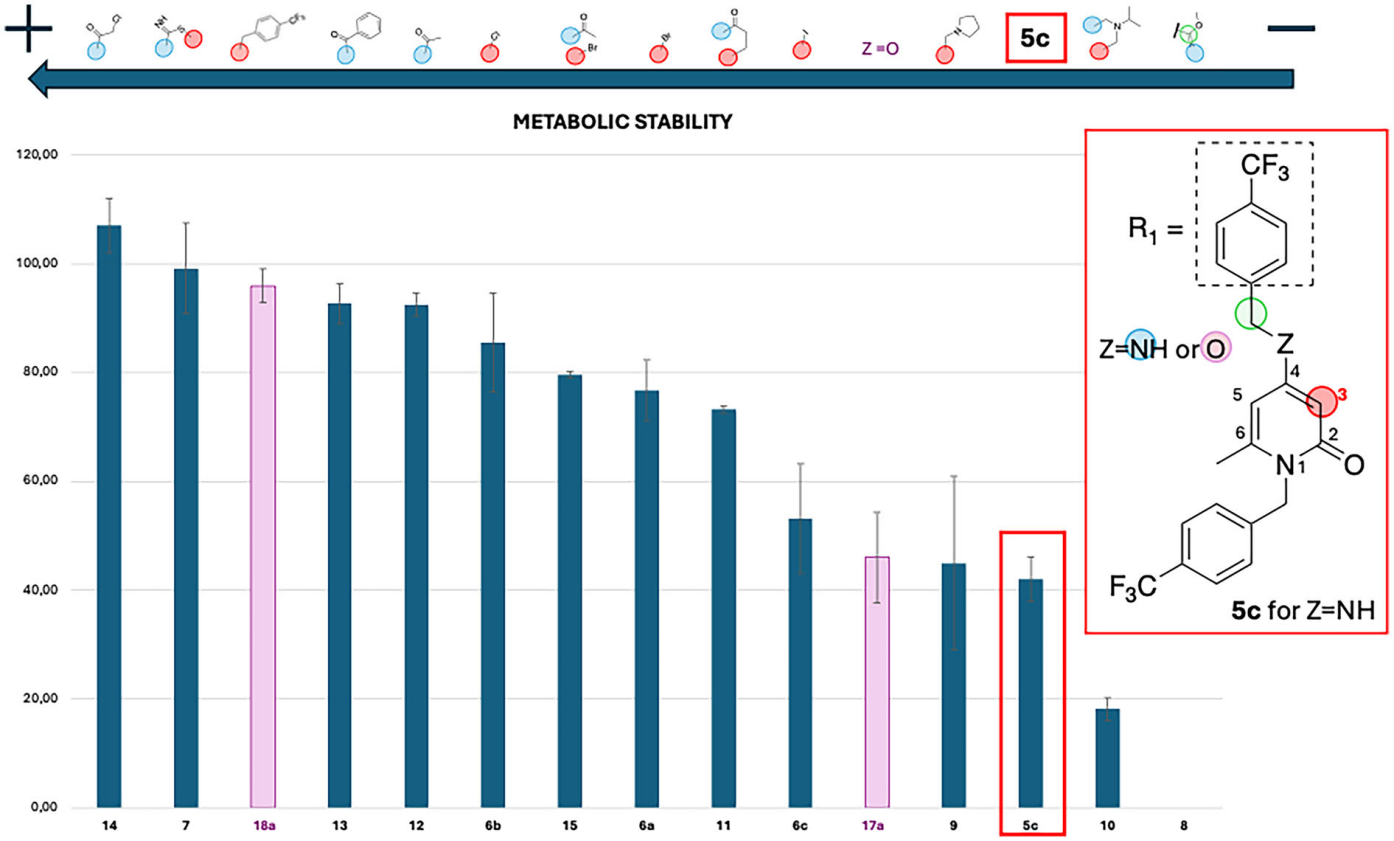
Metabolic stability data of the LSF derivatives of **5c** and 4‐akoxy‐2‐pyridones. Results are presented as the percentage of intact drug recovered relative to the control sample. LSF derivatives were incubated with MLM and an NADPH‐generating system for 60 min, followed by HPLC analysis. Control samples included compounds and liver microsomes without the NADPH system, allowing for the assessment of nonspecific binding to microsomes. Pink bars indicate the 4‐alkoxy‐2‐pyridone derivatives of compound **5c.**

All newly synthesized LSF derivatives of **5c** were biologically evaluated to determine whether the introduced modifications influenced both metabolic stability and anti‐PCSK9 activity. Microsomal stability assays were conducted to assess the impact of LSF modifications on improving the stability of **5c**. Results, expressed as the percentage of intact compound recovered relative to the control sample, are summarized in **Figure** **4**. In these assays, LSF derivatives were incubated with mouse liver microsomes (MLM) and an NADPH‐generating system for 60 min, followed by HPLC analysis. Control samples included compounds and liver microsomes without the NADPH system, allowing for the assessment of nonspecific binding to microsomes. As shown in Figure 4, modifications to both the C4‐amine and C3 positions significantly enhanced metabolic stability. Acylated derivatives **12**, **13**, and **14** achieved stability levels exceeding 95%, as did the bicyclic derivative **7**, which exemplifies a dual‐site functionalization. Among halogenated compounds, brominated **6b** demonstrated greater stability compared to chlorinated **6a**, while **6c** did not improve compared to the parent compound **5c**. However, combined functionalization in **15** did not result in improved stability over the single‐site modifications in **12** or **6b**. The MCR‐LSF product **11** showed a modest yet notable improvement in stability, whereas **9** did not exhibit any enhancements relative to **5c**. Compound **10** was less stable, likely due to rapid amine dealkylation of its newly introduced aliphatic amine group. Compound **8** was entirely metabolized, likely due to the hydrolysis‐prone enamine moiety. The 4‐alkoxy derivative **17a** did not show improved metabolic stability compared to **5c**, whereas the introduction of a 4‐trifluoromethylbenzyl group at the C3 in the dual functionalized derivative **18a** markedly enhanced the stability, reaching values above 95%. The other two synthesized analogs (**17b**, **18b**) did not show any improvement in metabolic stability compared to **5c** (data shown in Supporting Information, Figure S1). These results highlighted that the applied LSF strategies significantly improved metabolic stability in most cases. Exceptions were observed for MCR‐LSF products (**9** and **10**), for the Shono‐type derivative **8** and for the 4‐alkoxy derivatives **17a**, **17b**, **18b**.

### 2.2 Pharmacology

**Figure 5:**
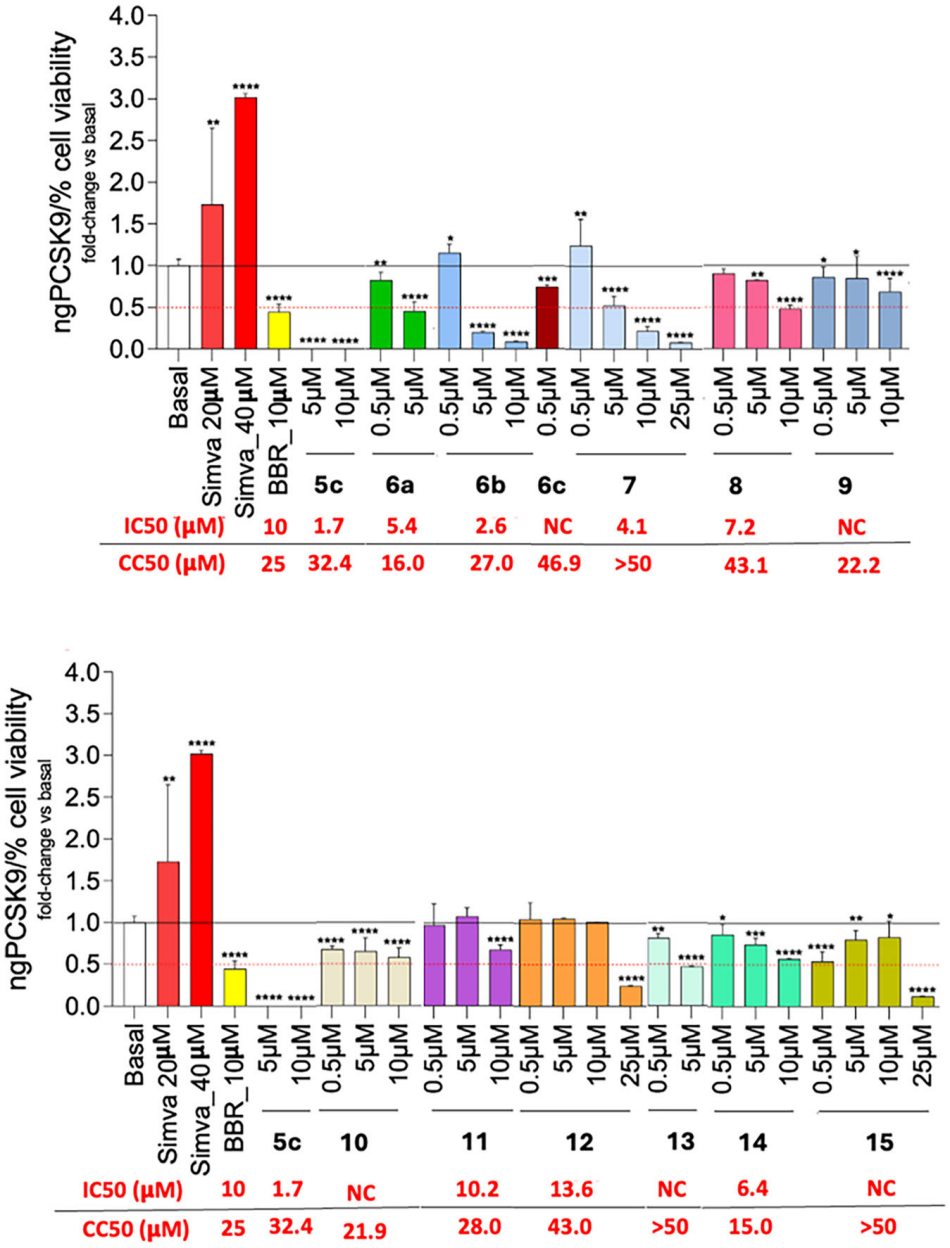
Effect of **5c** LSF derivatives on PCSK9 expression in HepG2 cell line. HepG2 cell cultures was treated with different concentrations of compounds for 24 h and PCSK9 secretion measured through ELISA assay on cell cultures supernatants. Only concentrations where cell viability was higher than 90% were selected for PCSK9 quantification. Data were normalized based on the percentage of HepG2 viability as determined by the MTT assay. Half‐maximal cytotoxic concentrations are indicated as CC_50_ values, while anti‐PCSK9 activities are indicated as IC_50_ in red below each graph. Data are presented as mean ± SD of two biological replicates. Statistical analysis was carried out using one‐way ANOVA followed by Tukey's multiple comparison test. NC: not calculable; *p < 0.05, **p < 0.01, ***p < 0.001, ****p < 0.0001 versus basal.

The approach we set‐up to test the new PCSK9i was based on functional assay, which evaluated at the same time compounds cytotoxicity, and their effect on the secreted PCSK9 on the same cell line. First, cell viability was evaluated in HepG2 cell cultures using the MTT assay (CC_50_ values are reported in **Table** **1** and **Figure** **5**) after 24 h of incubation in starving conditions (0.4% fetal bovine serum (FBS)). The low FBS concentration, and thus low extracellular lipoproteins, stimulates the sterol responsive element binding protein (SREBP) pathway, inducing the expression of LDLR, PCSK9, HMG‐CoA reductase,^[^ ^28^ ^]^ thus increasing the uptake of exogenous cholesterol and enhancing the synthesis of endogenous cholesterol. This first step allowed us to calculate the CC_50_ and identify the noncytotoxic concentrations (viability higher than 90%; Table 1), then tested for PCSK9 inhibitory activity.

**Table 1:** CC_50_ of LSF derivatives calculated from MTT assay results of compounds after 24 h incubation in HepG2 cell cultures at different concentrations (0.5; 5.0; 10.0; 25.0; 50.0 μM). For each compound the 95% confidence interval (CI) is reported.

Entry	Compound	CC_50_ (CI 95%) [μM]	Selected concentrations for ELISA assay
1	**6a**	16.0 (11.41–22.02)	0.5; 5.0
2	**6b**	27.0 (19.18–41.33)	0.5; 5.0; 10.0
3	**6c**	46.9 (20.21–485.4)	0.5
4	**7**	90.9 (54.35–331.6)	0.5; 5.0; 10.0; 25.0
5	**8**	30.2 (26.41–34.98)	0.5; 5.0; 10.0
6	**9**	22.2 (N/C)a)	0.5; 5.0; 10.0
7	**10**	21.9 (N/C)	0.5; 5.0; 10.0
8	**11**	28.0 (21,44–38.76)	0.5; 5.0; 10.0
9	**12**	43.0 (37.03–53.06)	0.5; 5.0; 10.0; 25.0
10b)	**13**	63.0 (23.08–4330)	0.5; 5.0
11	**14**	15.0 (N/C)	0.5; 5.0; 10.0
12	**15**	60.5 (N/C)	0.5; 5.0; 10.0; 25.0

a) N/C: CI not calculable;

b) The cytotoxic effect was non‐dose dependent.

Next, the levels of secreted PCSK9 were quantified by ELISA assay (Figure 5) only on supernatants collected from cells treated with noncytotoxic concentrations of each compound, as reported in Table 1. Simvastatin (Simva) at 20 and 40 μM and berberine (BBR) at 10 μM were included as controls. As described in the literature, simvastatin induces PCSK9 expression as a compensatory mechanism in response to its cholesterol‐lowering effects, being a pharmacological competitor for HMG‐CoA reductase, the rate‐limiting enzyme of the cholesterol biosynthetic pathway.^[^ ^29^ ^]^ In contrast, BBR, a natural compound, reduces the expression of PCSK9 levels through transcriptional inhibition, thus increasing the LDLR availability on plasma membrane.^[^ ^30^ ^]^ As additional and distinct mechanism to the latter, BBR is also able to stabilize the transcript of LDLR, thus increasing its half‐life.^[^ ^31^ ^]^ As expected, cells stimulated with simvastatin released more PCSK9 into the culture medium compared to unstimulated cells, with a statistically significant concentration‐dependent effect (+73% at 20 μM, *p* < 0.01; +200% at 40 μM, *p* < 0.0001), while BBR at 10 μM decreased PCSK9 levels in the medium more than 50% (*p* < 0.0001 vs. basal) (Figure 5).^[^ ^32^ ^]^

The brominated compound **6b** had a strong decreasing activity on PCSK9 secretion already at 5 μM (–75% vs. basal, *p* < 0.0001), with an increased efficacy at 10 μM (–90%, *p* < 0.0001). The bicyclic derivative **7** showed a perfect dose–response lowering of PCSK9 secreted amount from 5 to 25 μM (*p* < 0.0001 vs*.* basal for all of them), reaching a 74% and 90% decrease at 10 and 25 μM, respectively. In contrast, the MCR‐LSF products **9** and **11** showed a moderate but significant inhibition of PCSK9 secretion, which was more pronounced at higher concentrations (*p* < 0.0001 vs. basal for all the three compounds). In this MCR‐LSF series, compound **10** produced a quite flat 35%–40% reduction at all tested concentrations (0.5–10 μM). The iodinated and benzoylated derivatives (**6c** and **13**) demonstrated a suboptimal cytotoxicity profile (−20% viability at 5 μM for the former and ‐45% viability at 10 μM for the latter) which limited the quantification of PCSK9 levels to a maximum concentration of 0.5 μM for **6c** (−20% vs. control, *p* < 0.001) and of 0.5 μM (−17%, *p* < 0.01 vs. basal) and 5 μM (−51%, *p* < 0.0001 vs. basal) for compound **13**. The acetyl derivative **12** and the bromo‐acetyl derivative **15** did not inhibit PCSK9 in a dose‐dependent manner but were highly effective in reducing secreted PCSK9 at 25 μM (−75% and −80% vs*.* basal, both *p* < 0.0001). Moderate inhibitory activity was observed for the 3‐chlorinated compound **6a**, which showed significant −80% and −55% reductions at 0.5 μM (*p* < 0.01 vs. basal) and 5 μM (*p* < 0.0001 vs. basal), respectively. The Shono‐type derivative **8** was mostly effective at 10 μM, with a PCSK9 lowering capacity of 50% versus untreated cells (*p* < 0.0001), whilst the acylated compound **14** exhibited a more defined dose–response reduction profile of secreted PCSK9 but was a bit less effective than **8** at the highest concentration used (*p* < 0.0001 vs. basal). These results indicate that the most promising LSF derivatives are **6b** and **7** respect to **5c**, also showing a comparable or lower cytotoxicity in HepG2 cells. The calculated IC_50_ values on inhibitory effect on secreted PCSK9 are reported in Table S2, Supporting Information.

**Figure 6:**
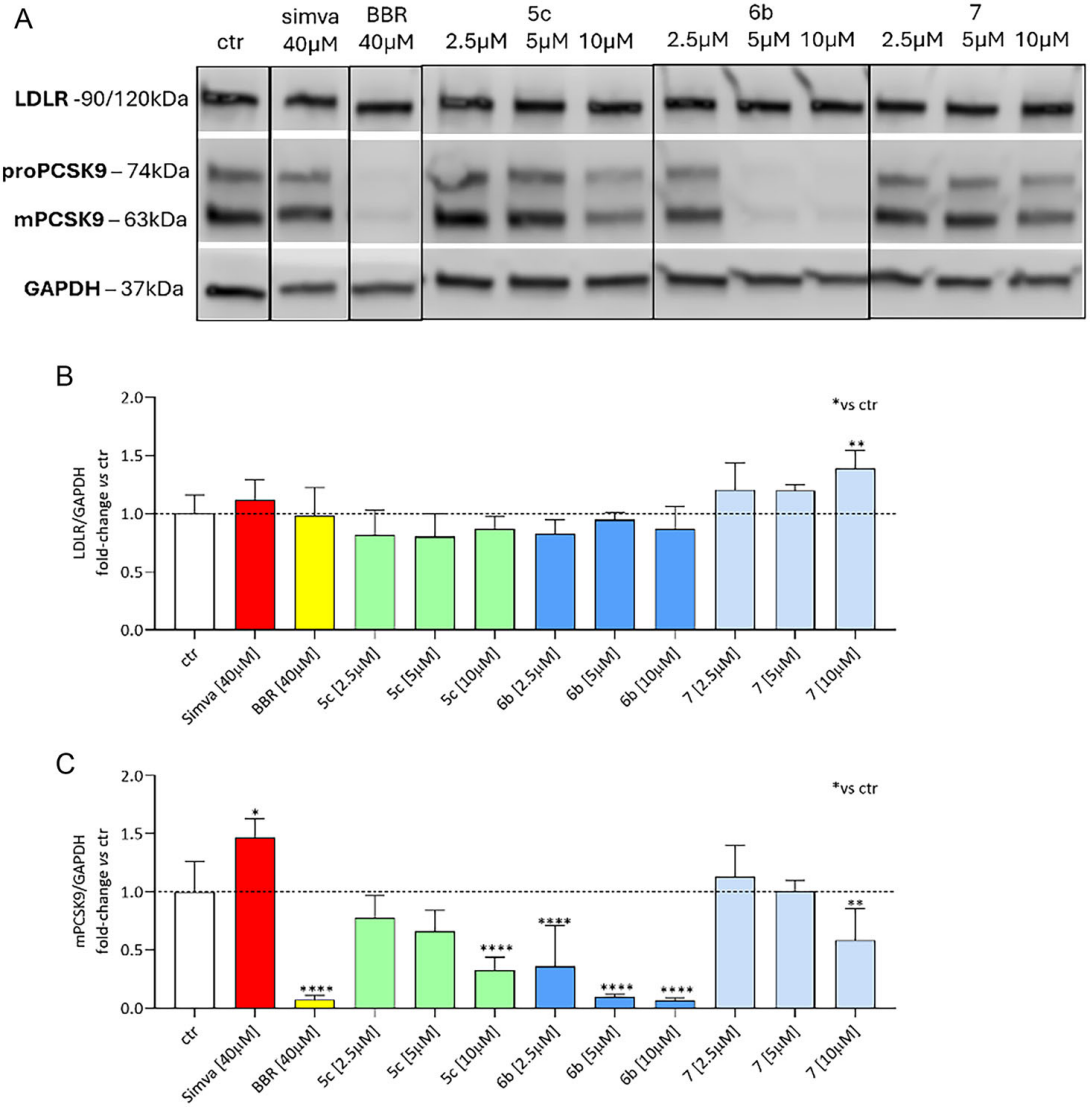
Effect of **5c** LSF derived compounds on PCSK9 and LDLR protein levels in HepG2 cell line. A) Western Blot and densitometric analyses of B) LDLR and C) PCSK9 protein levels after treatments with indicated concentrations of the compounds. Simvastatin (Simva) was used as known PCSK9 and LDLR inducer, whilst BBR as known PCSK9 silencer and LDLR inducer. Glyceraldehyde‐3‐phosphate dehydrogenase (GAPDH) was used as normalizing protein. Data are presented as mean ± SD of two biological replicates. Statistical analysis was carried out using one‐way ANOVA followed by Tukey's multiple comparison test. *p < 0.05, **p < 0.01, ***p < 0.001, ****p < 0.0001 versus basal.

The third step of our functional assay was focused on the most promising compounds from the previous step (**6b** and **7**), looking at the PCSK9 and LDLR protein expressions in HepG2 cells treated with increasing concentrations of the compounds (2.5–5–10 µM). This analysis, conducted in comparison with **5c** using a Western blot assay, aimed to provide a more comprehensive understanding of the intracellular activity and efficacy of these novel PCSK9i , further supporting their potential for preclinical development. As shown in **Figure** **6**, compound **6b** produced a significant and concentration‐dependent decrease in PCSK9 protein levels, reaching a maximal reduction of 93% at 10 µM (*p* < 0.0001) compared to basal condition, and pairing the PCSK9 lowering activity of BBR 40 μM (Figure 6A,C). In addition, in comparison with lead compound **5c** tested at the same concentrations, **6b** seems to be more effective in reducing the intracellular content of PCSK9. When looking at LDLR, **6b** did not modulate its expression at all concentrations tested, with a behavior similar to **5c**. Compound **7** produced a significant inhibition of PCSK9 only at 10 μM compared to basal condition (−42%, *p* < 0.01, Figure 6A,C). Importantly, LDLR protein levels were increased by compound **7** only at 10 µM (+39%, *p* < 0.01, Figure 6B) as compared to basal condition.

**Figure 7:**
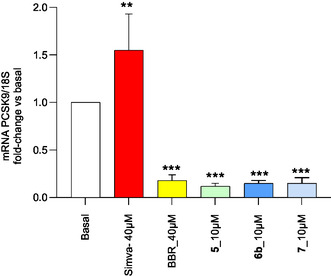
Effect of **5c** LSF derivatives on PCSK9 mRNA expression in HepG2 cell line. HepG2 cell culture was treated with indicated concentrations of compounds for 24 h and PCSK9 mRNA measured by RTqPCR. Data were given as mean ± SD of three independent experiments. Statistical analysis was carried out using Student's t‐test. **p < 0.01, ***p < 0.001 versus basal.

The lack of marked modulation on LDLR, together with a potent PCSK9 inhibitory effect, suggests a mechanism of action similar to BBR behavior for compounds **6b** and **7**. However, at 10 μM, both compounds reduced PCSK9 release into the medium more markedly than BBR (Figure 6A,C, −52% for BBR, −90% for compound **6b**, −74% for compound **7**), indicating a stronger potency. To further investigate the mechanism of action of these compounds, we evaluated the PCSK9 mRNA levels after 24 h incubation with increasing concentrations of **5c**, **6b,** and **7**. As shown in **Figure** **7**, all three compounds strongly suppressed the PCSK9 mRNA expression, indicating that their mechanism of action is likely related to an inhibitory effect on gene transcription.

**Figure 8:**
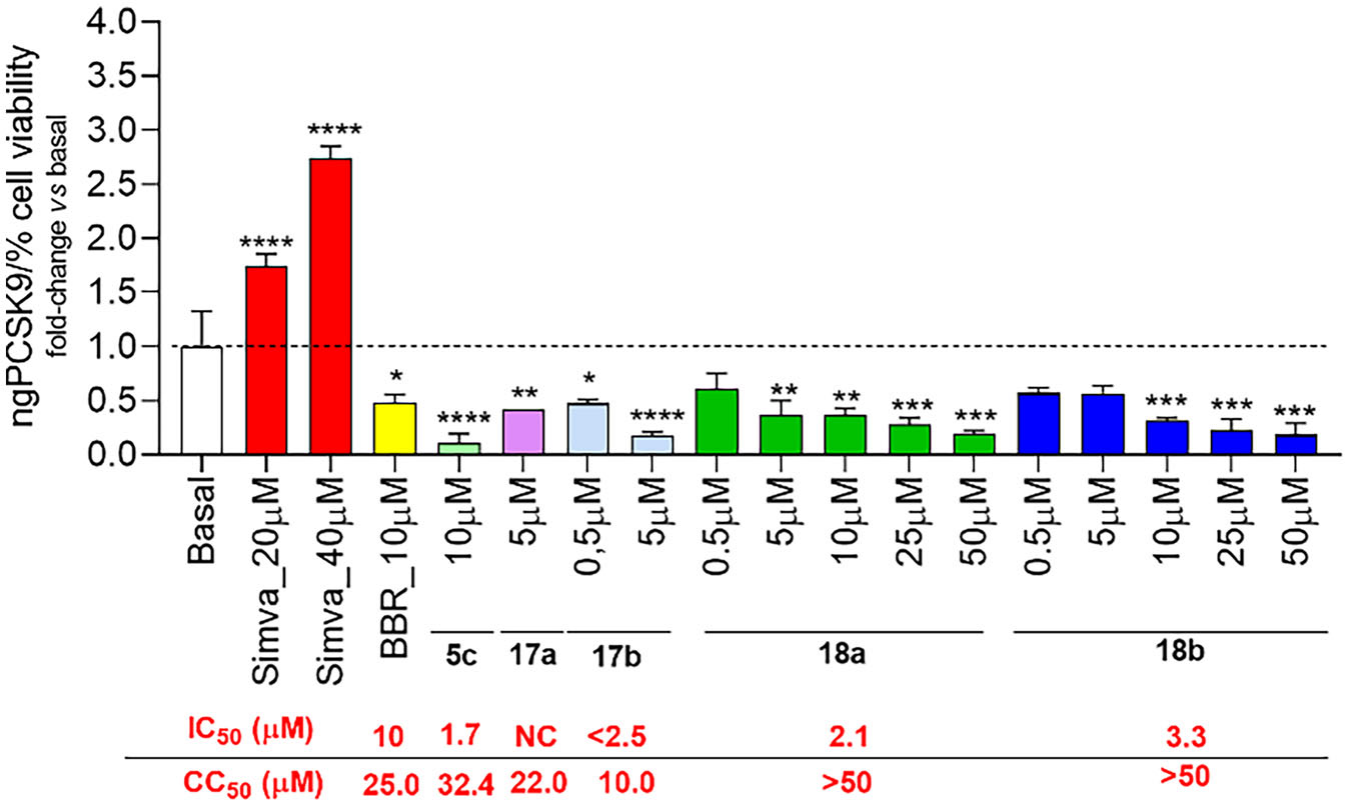
Effect of 4‐alkoxy‐2‐pyridones on PCSK9 expression in HepG2 cell line. HepG2 cell cultures was treated with different concentrations of compounds for 24 h and PCSK9 secretion measured through ELISA assay on cell cultures supernatants. Data were normalized based on the percentage of HepG2 viability as determined by the MTT asssay presented as mean ± SD of two biological replicates. Half‐maximal cytotoxic concentrations are indicated as CC_50_ values, while anti‐PCSK9 activities are indicated as IC_50_ in red below each graph. NC: not calculable; *p < 0.05, **p < 0.01, ***p < 0.001, ****p < 0.0001 versus basal.

Finally, the 4‐alkoxy‐2‐pyridone derivatives **17a**, **17b**, **18a,** and **18b** were preliminarily analyzed for their inhibitory activity on PCSK9 secretion, at the nontoxic concentrations previously determined (**Table** **2**). Compounds **17a** and **17b** showed a significant PCSK9 inhibitory activity at 5 μM (−58%, *p* < 0.01 and −82%, *p* < 0.0001, respectively vs. basal, **Figure** **8**). Interestingly, compounds **18a** and **18b**, nontoxic at all the concentrations tested, effectively inhibited PCSK9 secretion in a concentration‐dependent manner, reaching a −80% (*p* < 0.001) and −81% (*p* < 0.001) at 50 µM (IC_50_ of 2.1 and 3.3 µM, respectively, Figure 8). These results suggest a better selectivity index (CC_50_/IC_50_) of these derivatives as compared to the parent compound **5c**.

**Table 2:** CC_50_ of 4‐alkoxy‐2‐pyridone derivatives calculated from MTT assay results of compounds after 24 h incubation in HepG2 cell cultures at different concentrations (0.5; 5.0; 10.0; 25.0; 50.0 μM). For each compound, the 95% CI is reported.

Entry	Compound	CC_50_ (CI 95%) [μM]	Selected concentrations for ELISA assay
	**17a**	22.0 (N/C)a)	5.0
	**17b**	10.0 (N/C)a)	0.5; 5.0
	**18a**	210.9 (68.65–59,946)	0.5; 5.0; 10.0; 25.0; 50.0
	**18b**	72.6 (47.86–505.8)	0.5; 5.0; 10.0; 25.0; 50.0

a) N/C: CI not calculable.

### 2.3 Structure‐Metabolism‐Relationships (SMRs)

**Figure 9:**
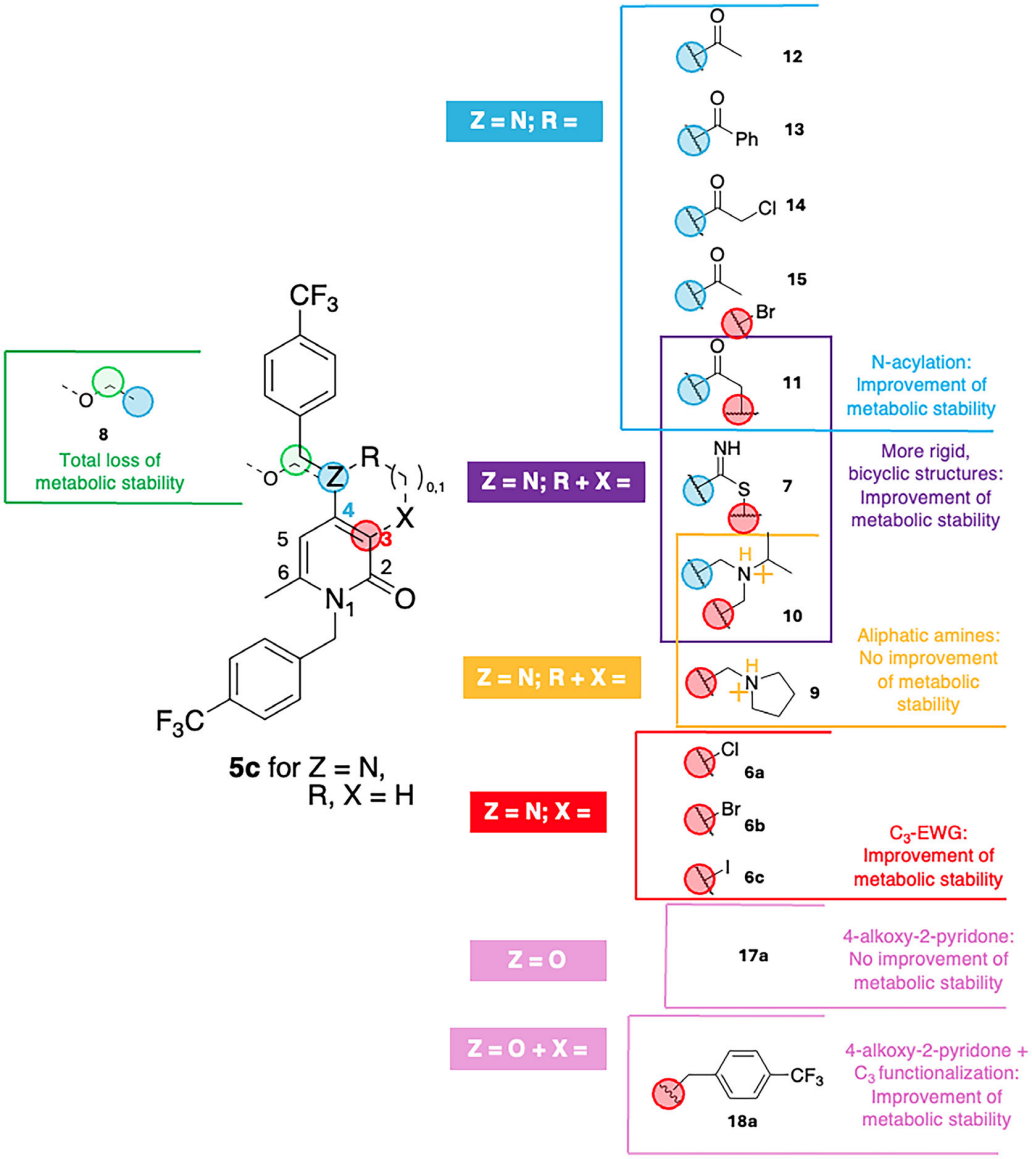
SMR of LSF‐ derivatives of **5c**. The 4‐alkoxy‐2‐pyridone derivatives are evidenced in pink; modifications on C3 are evidenced in red; The introduction of aliphatic amine groups are in yellow; the bicyclic derivatives are circled in purple; the N‐acylated compounds are evidenced in light blue; the benzylic metoxylation is evidenced in green.

In the search for orally available small‐molecule inhibitors of PCSK9, achieving a balance between potency and metabolic stability is a central challenge. SMRs offer critical insight into how structural modifications influence a compound's susceptibility to metabolic degradation, especially during first‐pass metabolism—a major barrier to oral bioavailability. In our study, LSF compounds were designed to retain the biological activity of the early lead compound **5c**, while significantly improving metabolic stability. This optimization is not merely incremental; it addresses a fundamental requirement for oral drug development. By demonstrating that these analogs maintain efficacy while reducing metabolic liability, we can provide a compelling case for their advancement and translational value to our optimization strategy. Based on the results reported in the previous sections, the following SMR were drawn (see also **Figure** **9**).

The introduction of electron‐withdrawing substituents, which reduce the electron density of the heterocycle—making it less susceptible to oxidative metabolic reactions—proved effective in enhancing metabolic stability. However, this modification did not always ensure the retention of anti‐PCSK9 activity. Notably, when halogens were introduced at the C3 position of the pyridone core (compounds **6a–c**), bromine emerged as the most effective in preserving metabolic stability. This is likely due to a combination of steric hindrance (as bromine is larger than chlorine) and electronegativity (as bromine is more electronegative than iodine). Compared to **5c**, the bromo‐derivative **6b** retained the inhibitory effect on PCSK9 secretion and was more effective in reducing the intracellular content of PCSK9, with a higher metabolic stability. Acylation of the nitrogen was explored both as a single‐site modification of this metabolic soft spot (compounds **12–14**) and as a dual‐site functionalization in the cases of the MCR‐LSF bicyclic derivative **11** and the acetylated bromo‐derivative **15**. All compounds **11–15** displayed improved metabolic stability, likely due to the steric hindrance introduced by the acyl groups, which protect the amine from metabolic transformation, as well as their electron‐withdrawing effects, which further decrease the electron density of the heteroaromatic scaffold. Among these, compound **11** exhibited significantly greater stability than **5c** but remained less stable than the other acetylated derivatives, possibly due to the presence of two newly introduced methylene groups, which may be susceptible to metabolic modifications. Regarding biological activity, these functionalizations yielded varied results, likely due to differences in geometry and steric hindrance introduced by the substituents. Among the acetylated derivatives, **12** and **13** demonstrated the most favorable balance of cytotoxicity and inhibitory activity. Similarly to the acylated derivatives on NHC4, the introduction of an imine moiety in the bicyclic derivative **7** led to a remarkable improvement in metabolic stability, with this compound showing the higher stability under experimental conditions. Compared to **5c**, **7** exhibited strong dose‐dependent inhibitory effect on PCSK9 secretion, a similar reduction of intracellular PCSK9, no reduction of LDLR expression, and a much higher metabolic stability. Conversely, the Shono‐type compound **8** and the derivatives obtained through MCR‐LSF with amines (**9–10**) displayed poor metabolic stability, likely due to the metabolic vulnerability of the newly introduced functional groups (the enamine in **8** and the aliphatic amine in **9** and **10**). These compounds also showed an unsatisfactory inhibitory profile, as **9** and **10** exhibited a nondose‐dependent response, while **8**'s evaluation was limited due to its cytotoxicity. Finally, the replacement of the C4 amine with an oxygen atom did not change much the metabolic stability but resulted in a compound (**17a**) with lower activity and higher cytotoxicity than **5c**. On the other hand, the dual‐site functionalization of NHC4 with oxygen, coupled with the benzylic functionalization in C3, led to a compound (**18a**) with high metabolic stability, low cytotoxicity and high efficacy in reducing the intracellular content of PCSK9.

## 3 Conclusion

In the present work, a chemistry‐driven LSF approach has been employed to conduct a sustainable medicinal chemistry campaign for the optimization of the early lead compound **5c**. Despite the excellent in vitro anti‐PCSK9 effect and in vivo tolerability, this molecule has shown poor in vitro metabolic stability with MLM that may limit its further development. Three metabolic soft spots within the structure of **5c** were selected for chemoselective modifications through LSF approaches or bioisosteric modification aimed at improving its metabolic stability while maintaining the anti‐PCSK9 activity. Overall, most of the planned functionalizations on the metabolic soft‐spots in C3 and NHC4 led to compounds with better metabolic stability than the parent compound **5c**, with the only exception of compounds **8** and **10,** which proved to be quite unstable after incubation with microsomes. The C3 functionalization with bulky electron‐withdrawing halogens (**6a–c**) significantly improved the metabolic stability, with the bromo‐substituted compound **6b** showing both high stability and anti‐PCSK9 activity, while alkylamines in the same position (**9**) did not improve the stability and lost efficacy. The NHC4 functionalization proved to be a robust strategy for metabolic stabilization. Acyl derivatives **12–14** showed high stability and a good balance between cytotoxicity and efficacy. The dual C3/NHC4 functionalizations also gave very promising results, especially for the bicyclic compound **7**, which emerged as one of the most promising candidates with strong PCSK9 inhibition and no metabolic stability degradation. Also the C3‐substituted 4‐alkoxy derivative **18a** showed an excellent metabolic stability while maintaining potent anti‐PCSK9 activity.

The present study demonstrates that LSF strategies focused on metabolic soft spots can effectively improve the microsomal stability of PCSK9 inhibitors while preserving or enhancing biological activity, an essential requirement for the development of orally available therapeutics. The most promising derivatives **6b**, **7,** and **18a** exhibited a higher metabolic stability/PCSK9 IC_50_ ratio with respect to **5c**, showing comparable or lower cytotoxicity in HepG2 cells. These compounds represent, therefore, promising candidates for further preclinical investigation and validation in other PCSK9‐related diseases.

## 4 Experimental Section

4.1

4.1.1

##### Chemistry

**General:** All commercially available chemicals were purchased from Fisher Scientifics and Fluorochem and, unless otherwise noted, used without any previous purification. Solvents used for work‐up and purification procedures were of technical grade. TLC was carried out using Sigma‐Aldrich TLC plates (silica gel on Al foils, SUPELCO Analytical, Merck 60 F254 silica plates). Where indicated, products were purified by silica gel flash chromatography on columns packed with Merck Geduran Si 60 (40–63 μm), or Merck 60 silica gel, 230–400 mesh. ^1^H, ^19^F, and ^13^C NMR spectra were recorded on BRUKER AVANCE 400 MHz and JEOL 600 MHz ECZ600R spectrometers. Chemical shifts (δ scale) are reported in parts per million relative to TMS. ^1^H NMR spectra are reported in this order: number of protons, multiplicity, coupling constant (*J*, in Hz); signals were characterized as: s (singlet), d (doublet), dd (doublet of doublets), dd (doublet of doublet of doublets), t (triplet), m (multiplet), bs (broad signal). ESI‐mass spectra were recorded on an API 150EX apparatus and are reported in the form of (m/z). Elemental analyses were performed by using a FlashEA 1112 series CHNS/O analyzer (Thermo Fisher) with gas‐chromatographic separation, and the data for C, H, and N are within 0.4% of the theoretical values. Microwave irradiation experiments were conducted using a CEM Discover Synthesis Unit (CEM Corp., Matthews, NC). The machine consists of a continuous focused microwave power delivery system with an operator‐selectable power output from 0 to 300 W. The temperature inside the reaction vessel was monitored using a calibrated infrared temperature control mounted under the reaction vessel. All experiments were performed using a stirring option whereby the reaction mixtures were stirred by means of a rotating magnetic plate located below the floor of the microwave cavity and a Teflon‐coated magnetic stir bar in the vessel. Electrochemistry experiments were carried out using an Electrasyn 2.0 apparatus.

##### Synthetic Procedures

eLSF derivatives (**6a–c**, **7**; **8**; **15**), MCR‐LSF derivatives (**9**; **10**), and acylation‐LSF derivative (**12**) were synthesized following the procedures reported in ref. [27].

##### Procedure for the Synthesis of the Early Lead 5c

According the general procedure A reported in ref. [21], in a 10 mL microwave tube, equipped with magnetic stir bar and septum, a mixture 4‐hydroxy‐6‐methyl‐2‐pyrone (100 mg, 1 equivalent, 0.79 mmol) and 4‐(trifluoromethyl)benzylamine (2 equivalents, 1.58 mmol, 138 μL) was heated at 100 °C for 6 min in the microwave apparatus (maximum power input: 300 W; maximum pressure: 250 psi; power max: OFF; stirring: ON). After cooling to room temperature, the solid was solubilized in AcOEt, washed with distilled H_2_O, and the organic phase extracted three times, then the collected organic layers were washed with brine, and finally dried over Na_2_SO_4_. Once removed the solvent under reduced pressure, the resulting crude was purified by flash chromatography (CHCl_3_/MeOH 99/1–98/2), affording the desired product as white solid in 55% yield (180.40 mg). ^1^H NMR (400 MHz, CDCl_3_) δ 7.505 (d, 2H, *J* = 7.83 Hz), 7.46 (d, 2H, *J* = 7.83 Hz), 7.33 (d, 2H, *J* = 7.83 Hz), 7.16 (d, 2H, *J* = 7.83 Hz), 5.53 (d, 1H, *J* = 1.89 Hz), 5.46 (d, 1H, *J* = 1.89 Hz), 5.18 (bs, 2H), 4.79 (bs, 1H), 4.26 (d, 2H, *J* = 4.60 Hz), 2.04 (s, 3H).^13^C NMR (100.6 MHz, CDCl_3_) δ 163.5, 153.9, 144.5, 140.7, 140.5, 128.8 (q, *J*_C–F_ = 32.51 Hz), 128.5 (q, *J*_C–F_ = 32.51 Hz), 126.4, 125.6, 124.7, 124.7, 123.0 (q, *J*_C–F_ = 271.69 Hz), 99.0, 89.9, 45.3, 44.9, 19.4. ^19^F NMR (564 MHz, CDCl_3_) δ – 62.43 (3F, s), −62.45 (3F, s). MS (ESI) m/z 441.2 [M+H]^+^, 463.3, [M+Na]^+^.

##### General Procedure for the Synthesis of e‐LSF Derivatives 6a–c, 7, 15

A 10 mL IKA Electrasyn electrochemical vial was charged with compound **5c or 12** (0.10 mmol, 1 equiv.), DDQ (4.54 mg, 0.02 mmol, 0.2 equiv.), the appropriate supporting electrolyte (0.2 mmol, 2 equiv.), TFA (depending on the case, catalytic amount, one drop), and MeOH (4 mL). The resulting mixture was then electrolyzed at a constant current of 10 mA until complete conversion of the starting material, as monitored by TLC analysis (2–4 F mol^–1^). Upon completion, the crude reaction mixture was poured into water and extracted with ethyl acetate. The combined organic extracts were washed with brine, dried over Na_2_SO_4_, filtered, and the solvent was removed under vacuum. The desired product was then obtained pure after flash chromatography purification.

##### 3‐Chloro‐6‐Methyl‐1‐(4‐(Trifluoromethyl)Benzyl)‐4‐((4‐(Trifluoromethyl)Benzyl)Amino)pyridin‐2(1H)‐One (6a)

According to GP2 of ref. [27], with compound **5c** as staring material (44.0 mg), using KCl (14.91 mg) as supporting electrolyte, and in the presence of TFA, after electrolyzing the reaction mixture for 4 F mol^–1^, the title compound (19.00 mg) was isolated by flash chromatography (dichloromethane/methanol 98/2) in 44% yield. White solid. m.p. 72–75 °C. ^1^H NMR (400 MHz, CDCl_3_) δ 7.56 (2H, d, *J* = 8.55 Hz), 7.48 (2H, d, *J* = 8.55 Hz), 7.35 (2H, d, *J* = 7.60 Hz), 7.21 (2H, d, *J* = 7.60 Hz), 5.56, (1H, s), 5.33 (1H, t, *J* = 5.70 Hz), 5.27 (2H, s), 4.48 (2H, d, *J* = 5.70 Hz), 2.10 (3H, s). ^13^C NMR (100.6 MHz, CDCl_3_) δ 158.5, 149.3, 143.6, 140.7, 139.8, 129.1 (q, *J*_C–F_ = 33.88 Hz), 128.8 (q, *J*_C–F_ = 31.88 Hz), 126.0, 125.9, 125.0, 124.9, 124.8, 124.7, 123.0 (2C, q, *J*_C–F_ = 271.78 Hz), 98.7, 94.0, 46.3, 45.3, 19.9. ^19^F NMR (564 MHz, CDCl_3_) δ −62.46 (3F, s), −62.48 (3F, s). MS (ESI) m/z 475.3, 477.1 [M+H]^+^.

##### 3‐Bromo‐6‐Methyl‐1‐(4‐(Trifluoromethyl)Benzyl)‐4‐((4‐(Trifluoromethyl)Benzyl)Amino)pyridin‐2(1H)‐One (6b)

According to GP2 of ref. [27], with compound **5c** as staring material (44.0 mg), using KBr (23.80 mg) as supporting electrolyte, and in the presence of TFA, after electrolyzing the reaction mixture for 4 F mol^–1^, the title compound (29.60 mg) was isolated by flash chromatography (dichloromethane/methanol 98/2) in 62% yield. White solid. m.p. 74–76 °C. ^1^H NMR (400 MHz, CDCl_3_) δ 7.55 (2H, d, *J* = 8.48 Hz), 7.47 (2H, d, *J* = 8.48 Hz), 7.34 (2H, d, *J* = 7.63 Hz), 7.19 (2H, d, *J* = 8.48 Hz), 5.52 (1H, s), 5.37 (1H, t, *J* = 5.85 Hz), 5.27 (2H, s), 4.47 (2H, d, *J* = 5.85 Hz), 2.08 (3H, s). ^13^C NMR (100.6 MHz, CDCl_3_) δ 159.8, 151.8, 145.4, 141.8, 141.0, 130.0 (m), 127.0, 126.9, 126.0, 125.9, 125.7, 125.7, 124.0 (2C, q, *J*_C–F_ = 272.1 Hz), 95.2, 91.2, 47.6, 46.5, 20.9. ^19^F NMR (564 MHz, CDCl_3_) δ −62.45 (3F, s), −62.48 (3F, s). MS (ESI) m/z 519.2, 521.2 [M+H]^+^.

##### 3‐Iodo‐6‐Methyl‐1‐(4‐(Trifluoromethyl)Benzyl)‐4‐((4‐(Trifluoromethyl)Benzyl)Amino)pyridin‐2(1H)‐One (6c)

According to GP2 of ref. [27], with compound **5c** as starting material (44.0 mg), using I_2_ (50.76 mg) as supporting electrolyte, after electrolyzing the reaction mixture for 2 F mol^–1^, the title compound (40.80 mg) was isolated by flash chromatography (dichloromethane/methanol 98/2) in 70% yield. Yellowish solid. m.p. 135–138 °C. ^1^H NMR (400 MHz, CDCl_3_) δ 7.56 (2H, d, *J* = 8.18 Hz), 7.47 (2H, d, *J* = 8.18 Hz), 7.34 (2H, d, *J* = 8.18 Hz), 7.19 (2H, d, *J* = 8.18 Hz), 5.33 (1H, t, *J* = 6.00 Hz), 5.29 (2H, s), 4.48 (2H, d, *J* = 6.00 Hz), 2.09 (3H, s). ^13^C NMR (100.6 MHz, CDCl_3_) δ 169.7, 163.8, 152.8, 147.6, 140.7, 139.8, 129.9 (2C, q, *J*_C–F_ = 33.25 Hz), 128.1, 126.8, 126.0 (2C), 125.7 (2C), 124.0 (2C, q, *J*_C–F_ = 272.15 Hz), 114.3, 106.5, 51.5, 46.9, 22.8, 20.8. ^19^F NMR (564 MHz, CDCl_3_) δ −62.45 (3F, s), −62.48 (3F, s). MS (ESI) m/z 567.2 [M+H]^+^.

##### 2‐Imino‐6‐Methyl‐1,5‐Bis(4‐(Trifluoromethyl)benzyl)‐1,2‐Dihydrothiazolo[5,4‐c]pyridin‐4(5H)‐One (7)

According to GP2 of ref. [27], with compound **5c** as staring material (44.0 mg), using KSCN (19.44 mg) as supporting electrolyte, after electrolyzing the reaction mixture for 4 F mol^–1^, the title compound (13.43 mg) was isolated by flash chromatography (Petroleum ether/AcOEt 9/1–8/2) in 29% yield. Yellow solid. m.p. 155–160 °C. ^1^H NMR (400 MHz, CDCl_3_) δ 7.54 (2H, d, *J* = 8.28 Hz), 7.49 (2H, d, *J* = 8.28 Hz), 7.32 (2H, d, *J* = 8.28 Hz), 7.20 (2H, d, *J* = 8.28 Hz), 7.57 (1H, s), 5.30 (2H, s), 5.08 (2H, s), 2.18 (3H, s). ^1^H NMR (400 MHz, DMSO‐d_6_) δ 8.84 (1H, bs), 7.74–7.70 (4H, m), 7.53 (2H, d, *J* = 7.97 Hz), 7.35 (2H, d, *J* = 7.97 Hz), 6.47 (1H, s), 5.37 (2H, s), 5.18 (2H, s), 2.26 (3H, s). ^13^C NMR (100.6 MHz, CDCl_3_) δ 163.0, 156.9, 146.7, 146.0, 140.3, 139.5, 130.1 (2C, q, *J*_C–F_ = 32.39 Hz), 127.03, 126.9, 126.0 (2C), 125.9, 122.4 (2C, q, *J*_C–F_ = 272.1 Hz), 103.9, 94.4, 46.8, 45.8, 21.3. ^19^F NMR (564 MHz, CDCl_3_) δ −62.53 (3F, s), −62.54 (3F, s). MS (ESI) m/z 498.1 [M+H]^+^.

##### N‐(3‐Bromo‐6‐Methyl‐2‐Oxo‐1‐(4‐(Trifluoromethyl)Benzyl)‐1,2‐Dihydropyridin‐4‐yl)‐N‐(4‐(Trifluoromethyl)Benzyl)Acetamide (15)

According to GP2 of ref. [27], with compound **12** as staring material (48.2 mg), using KBr (23.80 mg) as supporting electrolyte, and in the presence of TFA, after electrolyzing the reaction mixture for 4 F mol^–1^, the title compound (9.0 mg) was isolated by flash chromatography (dichlorometane 100%–dichloromethane/acetone 95/5) in 18% yield. White solid. m.p. 73–75 °C. ^1^H NMR (400 MHz, CDCl_3_) δ 7.53 (2H, d, *J* = 8.16 Hz), 7.48 (2H, d, *J* = 7.70 Hz), 7.33 (2H, d, *J* = 8.16 Hz), 7.20 (2H, d, *J* = 7.70 Hz), 5.62 (1H, s), 5.32 (2H, d, *J* = 5.22 Hz), 5.26 (1H, d, *J* = 14.70 Hz), 4.35 (1H, d, *J* = 14.70 Hz), 2.14 (3H, s), 1.95 (3H, s). ^13^C NMR (100.6 MHz, CDCl_3_) *δ* 167.7, 159.6, 150.0, 144.9, 139.3, 137.9, 129.1 (2C, q, *J*_C–F_ = 32.51 Hz), 128.1, 125.9, 124.9, 124.8, 124.2, 122.8 (q, *J*_C–F_ = 273.5 Hz), 122.6 (q, *J*_C‐F_ = 270.1 Hz), 113.5, 107.0, 49.1, 47.6, 21.0, 19.4. ^19^F NMR (564 MHz, CDCl_3_) δ −62.47 (3F, s), −62.63 (3F, s). MS (ESI) m/z 561.3, 563.3 [M+H]^+^.

##### Procedure for the Synthesis of e‐LSF Derivative Methyl (Z)‐N‐(6‐Methyl‐2‐Oxo‐1‐(4‐(Trifluoromethyl)Benzyl)‐1,2‐Dihydropyridin‐4‐yl)‐4‐(Trifluoromethyl)Benzimidate (8)

According to GP3 of ref. [27], with compound **5c** as staring material (44.0 mg, 0.1 mmol), using NaCN (9.80 mg, 0.2 mmol, 2 equiv.) as supporting electrolyte, after electrolyzing the reaction mixture for 8 F mol^–1^ at a constant current of 1.5 mA, the title compound (10.0 mg) was isolated by flash chromatography (petroleum ether/ethyl acetate 60/40–50/50) in 24% yield. Yellowish solid. m.p. 80–90 °C. ^1^H NMR (400 MHz, CDCl_3_) δ 7.5 (4H, s), 7.48 (2H, *J* = 8.00 Hz, s), 7.13 (2H, d, *J* = 8.00 Hz), 5.71 (1H, d, *J* = 2.00 Hz), 5.62 (1H, s), 5.25 (2H, s), 3.89 (3H, s), 2.11 (3H, s). ^13^C NMR (100.6 MHz, CDCl_3_) δ 163.0, 156.6, 156.5, 145.1, 139.5, 132.4, 131.5, 128.4 (2C, q, *J*_C‐F_ = 33.34 Hz), 128.0, 125.3, 124.49, 124.46, 124.0 (2C, q, *J*_C–F_ = 270.1 Hz), 103.7, 103.3, 53.4, 45.0, 28.4, 19.3. ^19^F NMR (564 MHz, CDCl_3_) δ −62.53 (3F, s), −62.54 (3F, s). MS (ESI) m/z 469.2 [M+H]^+^.

##### Procedure for the Synthesis of MCR‐LSF Derivative 6‐Methyl‐3‐(Pyrrolidin‐1‐ylmethyl)‐1‐(4‐(Trifluoromethyl)Benzyl)‐4‐((4‐(Trifluoromethyl)Benzyl)Amino)pyridin‐2(1H)‐One (9)

Following GP4 of ref. [27], a microwave tube was charged with **5c** (1 equiv., 44.0 mg, 0.1 mmol), pyrrolidine (2 equiv., 0.2 mmol, 14.22 mg, 16.42 μL), formaldehyde (0.15 mmol, 1.5 equiv.) acetic acid (20 mol%), and acetonitrile/distilled water (1:1, 2 mL). The resulting mixture was heated at 80 °C in the microwave apparatus in sealed tube (maximum power input: 300 W; maximum pressure: 250 psi; power max: OFF; stirring: ON) for 40 min, verifying the complete conversion of the starting material by TLC monitoring. Then saturated aqueous NaHCO_3_ was added, and the aqueous phase was extracted three times with dichloromethane, the combined organic layers dried over Na_2_SO_4_, and the solvent evaporated under reduced pressure. The obtained crude of the reaction was purified by flash chromatography (dichloromethane/methanol 90/10), affording the desired product as yellowish solid in 71% yield. White solid. m.p. 120–123 °C. ^1^H NMR (400 MHz, CDCl_3_) δ 8.19 (1H, s), 7.62 (2H, d, *J* = 7.79 Hz), 7.56 (2H, d, *J* = 7.79 Hz), 7.45 (2H, d, *J* = 8.12 Hz), 7.25 (2H, d, *J* = 8.12 Hz), 5.60 (1H, s), 5.30 (2H, s), 4.52 (2H, d, *J* = 5.91 Hz), 4.03 (2H, s), 2.87 (4H, s), 2.15 (3H, s), 1.88 (4H, s). ^13^C NMR (100.6 MHz, CDCl_3_) δ 163.4, 154.6, 146.7, 141.8, 140.0, 128.7, (q, *J*_C–F_ = 30.79 Hz), 128.4 (q, *J*_C–F_ = 32.02 Hz), 126.1, 125.3, 124.8 (2C), 124.6 (2C), 123.11 (q, *J*_C–F_ = 272.1 Hz), 122.9 (q, *J*_C–F_ = 272.1 Hz), 95.5, 92.0, 51.5, 48.8, 45.7, 45.0, 22.1, 20.2. ^19^F NMR (564 MHz, CDCl_3_) δ −62.33 (3F, s), −62.50 (3F, s). MS (ESI) m/z 524.2 [M+H]^+^.

##### Procedure for the Synthesis of MCR‐LSF Derivative 3‐Isopropyl‐7‐Methyl‐1,6‐Bis(4‐(Trifluoromethyl)Benzyl)‐2,3,4,6‐Tetrahydropyrido[4,3‐d]pyrimidin‐5(1H)‐One (10)

Following GP5 of ref. [27], a 10 mL round‐bottomed flask was charged with compound **5c** (44.0 mg, 0.1 mmol, 1 equiv.), isopropyl amine **23** (11.82 mg, 17.03 μL, 0.2 mmol, 2 equiv.), formaldehyde (0.4 mmol, 4 equiv.), acetic acid (20 mol%), and acetonitrile/distilled water (1:1, 2 mL). The resulting mixture was heated at 80 °C in the microwave apparatus (maximum power input: 300 W; maximum pressure: 250 psi; power max: OFF; stirring: ON) for 20 min, verifying the complete conversion of the starting material by TLC monitoring. Then saturated aqueous NaHCO_3_ was added, and the aqueous phase was extracted three times with dichloromethane, the combined organic layers dried over Na_2_SO_4_, and the solvent evaporated under reduced pressure. The obtained crude of the reaction was purified by flash chromatography (ethyl acetate/methanol 100/0–97/3), affording the desired product as white solid in 40% yield (20.5 mg). White solid. m.p. 83–87 °C. ^1^H NMR (400 MHz, CDCl_3_) δ 7.64 (2H, d, *J* = 8.11 Hz), 7.57 (2H, d, *J* = 8.11 Hz), 7.41 (2H, d, *J* = 8.11 Hz) 7.28 (2H, d, *J* = 8.11 Hz), 5.63 (1H, s), 5.34 (2H, s), 4.57 (2H, s), 4.10 (2H, s), 3.85 (2H, s), 3.00–2.95 (1H, m), 2.15 (3H, s), 1.18 (3H, s), 1.16 (3H, s). ^13^C NMR (100.6 MHz, CDCl_3_) δ 161.6, 150.5, 143.8, 141.9, 141.6, 129.8 (q, *J*_C–F_ = 39.83 Hz), 129.5 (q, *J*_C–F_ = 40.06 Hz), 126.8, 125.9 (2C), 125.7 (2C), 124.1 (2C, q, *J*_C–F_ = 277.54 Hz), 99.9, 96.2, 67.1, 52.5, 51.4, 46.1 29.7, 20.9, 19.9. ^19^F NMR (564 MHz, CDCl_3_) δ −62.40 (3F, s), −62.41 (3F, s). MS (ESI) m/z 524.2 [M+H]^+^.

##### Procedure 6 for the Synthesis of MCR‐LSF Derivative 7‐Methyl‐1,6‐Bis(4‐(Trifluoromethyl)Benzyl)‐4,6‐Dihydro‐1,6‐Naphthyridine‐2,5(1H,3H)‐Dione (11)

A 10 mL flask was charged with compound **5c** (44.04 mg, 0.1 mmol, 1 equiv.), Meldrum acid (0.4 mmol, 4 equiv.), formaldehyde (0.35 mmol, 3.5 equiv.), acetic acid (20 mol%), and acetonitrile/distilled water (1:1; 2.0 mL). The resulting mixture was heated at 80 °C for 24 h, verifying the complete conversion of the starting material by TLC monitoring. Then distilled water was added, and the aqueous phase extracted three times with dichloromethane, the combined organic layers were dried over Na_2_SO_4_ and then solvent evaporated under reduced pressure. The obtained crude of the reaction was purified by flash chromatography (Hexane/Ethyl Acetate 4/6), affording the desired product as yellowish solid (21.70 mg, 0.044 mmol, 44%). ^1^H NMR (400 MHz, CDCl_3_) δ 7.63–7.58 (4H, m), 7.33–7.27 (4H, m), 5.76 (1H, s), 5.37 (2H, s), 5.16 (2H, s), 3.00 (2H, t, *J* = 7.42 Hz), 2.82 (2H, t, *J* = 7.42 Hz), 2.21 (3H, s). ^13^C NMR (100.6 MHz, CDCl_3_) δ 169.4, 161.0, 145.9, 143.8, 139.4, 139.1, 128.7, 125.5, 125.2, 124.7, 124.0, 106.9, 96.6, 45.8, 43.9, 29.7, 28.4, 19.9, 17.5.^19^F NMR (564 MHz, CDCl_3_) δ –62.53 (3F, s), –62.46 (3F, s). MS (ESI) *m*/*z* 495.1 [M+H]^+^.

##### General Procedure for the Synthesis of Acylation‐LSF 12–13

In a 10 mL microwave tube, equipped with magnetic stir bar and septum, a mixture of compound **5c** (0.10 mmol, 44.0 mg), the proper acyl chloride (2.5 equiv., 0.25 mmol), and pyridine (2.5 equiv., 0.25  mmol, 20.14 μL) in anhydrous DCM (1.5 mL) was heated at 45 °C for 30 min in the microwave apparatus (maximum power input: 300 W; maximum pressure: 250 psi; power max: OFF; stirring: ON). After cooling to room temperature, the solution was diluted with ethyl acetate and washed with NaHCO_3_ sat. sol. The aqueous phase was then extracted with ethyl acetate (three times), and the collected organic layers washed with brine, dried over Na_2_SO_4_, filtered, and the solvent removed under reduced pressure. The crude of the reaction was then purified by flash chromatography, affording the desired products.

##### N‐(6‐Methyl‐2‐Oxo‐1‐(4‐(Trifluoromethyl)Benzyl)‐1,2‐Dihydropyridin‐4‐yl)‐N‐(4‐(Trifluoromethyl)Benzyl)Acetamide (12)

Using acetyl chloride (2.5 eq, 0.25 mmol, 17.77 μL), as acyl chloride, the title compound was obtained after purification (DCM/MeOH 98/2) in 53% yield (46.00 mg) as yellowish solid. m.p. 82–84 °C. ^1^H NMR (400 MHz, CDCl_3_) δ 7.52–7.49 (m, 4H), 7.28 (2H, d, *J* = 8.37 Hz), 7.17 (2H, d, *J* = 8.37 Hz), 6.19 (1H, d, *J* = 2.30 Hz), 5.84 (1H, s), 5.26 (2H, s), 4.85 (2H, s), 2.18 (3H, s), 2.09 (3H, s). ^13^C NMR (100.6 MHz, CDCl_3_) δ 168.6, 162.7, 151.7, 146.5, 139.6, 138.7, 128.9 (q, *J*_C–F_ = 32.83 Hz), 128.8 (q, J_C–F_ = 32.51 Hz), 126.9, 125.7, 124.8, 124.8, 124.6, 124.5, 122.9 (q, *J*_C–F_ = 272.08 Hz), 121.8 (q, *J*_C–F_ = 272.08 Hz), 113.1, 105.4, 50.3, 45.8, 21.7, 19.7. ^19^F NMR (564 MHz, CDCl_3_) δ −62.47 (3F, s), −62.57 (3F, s). MS (ESI) m/z 483.1 [M+H]^+^.

##### N‐(6‐Methyl‐2‐Oxo‐1‐(4‐(Trifluoromethyl)Benzyl)‐1,2‐Dihydropyridin‐4‐yl)‐N‐(4‐(Trifluoromethyl)Benzyl)Benzamide (13)

Using benzoyl chloride (2.5 equiv., 0.37 mmol, 52.71 mg, 43.56 μL) as acyl chloride, the title product was obtained after flash chromatography (Hexane/Ethyl Acetate 6/4 in 40% yield (32.67 mg, 0.06 mmol) as yellowish solid. ^1^H NMR (400 MHz, CDCl_3_) δ 7.52 (d, *J* = 7.77 Hz, 2H), 7.47 (d, *J* = 7.34 Hz, 4H), 7.39 (d, *J* = 8.20 Hz, 2H), 7.36 (d, *J* = 7.34 Hz, 1H), 7.28–7.24 (m, 2H), 7.02 (d, *J* = 8.20 Hz, 2H), 6.14 (s, 1H), 5.59 (s, 1H), 5.18 (s, 2H), 5.09 (s, 2H), 1.98 (s, 3H). ^13^C NMR (100.6 MHz, CDCl_3_) δ 169.4, 162.6, 152.5, 145.3, 139.8, 138.9, 133.8, 132.2, 130.4, 129.1 (m), 128.8 (m), 127.6, 127.3, 126.9, 125.4, 124.8, 124.8, 124.7, 124.7, 123.0 (q, *J*_C–F_ = 272.03 Hz), 122.9 (q, *J*_C–F_ = 272.03 Hz), 110.9, 105.8, 51.1, 45.5, 19.5. ^19^ F NMR (564 MHz, CDCl_3_) δ −62.54 (3F, s), −62.47 (3F, s). MS (ESI) *m*/*z* 545.1 [M+H]^+^ **.**

##### Procedure 8 for the Synthesis of Acylation‐LSF derivative 2‐Chloro‐N‐(6‐Methyl‐2‐Oxo‐1‐(4‐(Trifluoromethyl)Benzyl)‐1,2‐Dihydropyridin‐4‐yl)‐N‐(4‐(Trifluoromethyl)Benzyl)Acetamide (14)

An oven‐dried flask was charged under Argon protection with **5c** (1 equiv., 0.15 mmol, 66.06 mg), freshly distilled DCM (2.0 mL), and Et_3_N (4.0 equiv., 0.6 mmol, 60.71 mg, 83.62 μL), the resulting mixture was cooled down to 0 °C (ice bath), chloroacetyl chloride (4.0 eq, 0.6 mmol, 67.76 mg, 47.75 μL) was slowly added and the final reaction mixture refluxed overnight. Upon complete conversion of the starting material, monitored by TLC analysis, the reaction was quenched by slowly adding distilled H_2_O, the aqueous phase was then extracted three times with DCM; and the collected organic layers washed with brine, dried over Na_2_SO_4_, filtered and the solvent removed under reduced pressure. The collected crude of reaction was purified through flash chromatography (DCM/Acetone 8/2), affording the desired product (57.00 mg, 0.11 mmol, 75%). ^1^H NMR (400 MHz, CDCl_3_) δ 7.52–7.49 (4H, m), 7.29 (2H, d, *J* = 8:04 Hz), 7.16 (2H d, *J* = 8.07 Hz), 6.30 (1H, d, *J* = 2.33 Hz), 5.89 (1 H, d, *J* = 1.71 Hz), 5.27 (2H, s), 4.87 (2H, s), 4.07 (2H, s), 2.21 (3H, s). ^13^C NMR (100.6 MHz, CDCl_3_) δ 169.2, 166.0, 163.8, 151.5, 148.5, 139.8, 139.4, 130.3 (q, *J*_C–F_ = 32.66 Hz), 130.2 (q, *J*_C–F_ = 32.66 Hz), 128.3, 126.8, 126.0, 126.0, 125.8, 125.8, 124.2 (q, *J*_C–F_ = 273.30 Hz), 123.9 (q, *J*_C–F_ = 271.73 Hz), 114.7, 106.4, 52.1, 47.2, 41.5, 20.9. ^19^ F NMR (564 MHz, CDCl_3_) δ –62.58 (3F, s), –62.52 (3F, s). MS (ESI) *m*/*z* 517.1 [M+H]^+^.

##### General Procedure for the Synthesis of 16a–b

To a solution of 4‐hydroxy‐6‐methyl‐2‐pyrone (500 mg, 3.96 mmol, 1 equiv.) in H_2_O (10 mL), the proper amine (3.96 mmol, 1 equiv.) was added. The resulting mixture was refluxed until complete conversion of the starting materials (3–24 h), monitored by TLC. The solid mass was filtrated under vacuum, washed with diethyl ether and purified by silica gel flash chromatography to afford the desired compounds as white solids.

##### 4‐Hydroxy‐6‐Methyl‐1‐(4‐(Trifluoromethyl)Benzyl)pyridin‐2(1H)‐One (16a)

Using 4‐(trifluoromethyl)benzylamine (564.3 μL) as amine, the desired product was obtained after 3.5 h of reaction and flash chromatography (DCM/MeOH 99/1) in 40% yield (449.00 mg, 1.58 mmol). ^1^H NMR (300 MHz, DMSO‐d_6_) δ 10.59 (1H, bs), 7.69 (2H, d, *J* = 8.97 Hz), 7.30 (2H, d, *J* = 7.98 Hz), 5.84 (1H, dd, *J* = 2.64, *J* = 0.81 Hz), 5.62 (1H, d, *J* = 2.61 Hz), 5.27 (2H, bs), 2.16 (3H, s). MS (ESI) m/z 284.1[M+H]^+^.

##### 1‐(4‐Chlorobenzyl)‐4‐Hydroxy‐6‐Methylpyridin‐2(1H)‐One (16b)

Using 4‐(chloro)benzylamine (481.7 μL) as amine, the desired product was obtained pure after flash chromatography (DCM/MeOH 99/1) in 55% yield (538.00 mg, 2.18 mmol). ^1^H NMR (300 MHz, DMSO‐d_6_) δ 10.50 (1H, bs), 7.39 (2H, d, *J* = 8.58 Hz), 7.12 (2H, d, *J* = 8.64 Hz), 5.80 (1H, dd, *J* = 2.64, *J* = 0.81 Hz), 5.59 (1H, d, *J* = 3.58 Hz), 5.16 (2H, bs), 2.16 (3H, s). MS (ESI) m/z 248.1[M+H]^+^.

##### General Procedure for the Synthesis of 17a–b and 18a–b

An oven‐dried flask was charged under argon protection with **16a–b** (0.18 mmol, 1 equiv.), oven‐dried K_2_CO_3_ (49.75 mg, 0.36 mmol, 2 equiv.), dry DMF (1.80 mL) and the proper benzylbromide (0.22 mmol, 1.2 equiv.); the resulting mixture was stirred at room temperature until complete conversion of the starting material, monitored by TLC analysis (2–12 h). DMF was then diluted with CHCl_3_, and the organic phase washed several times with LiCl saturated solution and brine, then dried over Na_2_SO_4_, filtered and the organic solvent removed under reduced pressure, affording the crude of the reaction. Finally, the products were isolated from the crude of the reaction through flash chromatography (Hexane/AcOEt, 8/2 to 7/3).

##### (6‐Methyl‐1‐(4‐(Trifluoromethyl)Benzyl)‐4‐((4‐(Trifluoromethyl)Benzyl)Oxy)pyridin‐2(1H)‐One) (17a)

Yield%: 39%.^1^H NMR (400 MHz, CDCl_3_) δ 7.59 (2H, d, *J* = 8.12 Hz), 7.50 (2H, d, *J* = 7.90 Hz), 7.45 (2H, d, *J* = 8.12 Hz), 7.19 (2H, d, *J* = 7.90 Hz), 5.88 (1H, s), 5.82 (1H, s), 5.26 (2H, s), 5.00 (2H, s), 2.15 (3H, s). ^13^C NMR (100.6 MHz, CDCl_3_) δ 165.0, 163.7, 145.0, 139.5, 138.0, 129.2 (q, *J*_C–F_ = 33.34 Hz), 128.3 (q, *J*_C–F_ = 33.34 Hz), 126.2, 125.3, 124.4, 124.4, 124.4, 124.3, 124.3, 122.7 (q, *J*_C–F_ = 272.26 Hz), 122.6 (q, *J*_C–F_ = 270.27 Hz), 100.4, 94.5, 67.7, 44.9, 19.1. ^19^ F NMR (564 MHz, CDCl_3_) δ −62.55 (3F, s), −62.47 (3F, s). MS (ESI) *m*/*z* 442.1 [M+H]^+^ **.**

##### (1‐(4‐Chlorobenzyl)‐4‐((3‐Fluorobenzyl)Oxy)‐6‐Methylpyridin‐2(1H)‐One) (17b)

Yield%: 48%. ^1^H NMR (400 MHz, CDCl_3_) δ 7.29–7.24 (1H, s), 7.17 (2H, d, *J* = 2.18 Hz), 7.07 −7.03 (1H, m), 7.00 (2H, d, *J* = 8.11 Hz), 6.95 (1H, dt, *J* = 8.11 Hz, 1.87 Hz), 5.85 (1H, d, *J* = 2.92 Hz), 5.77 (1H, d, *J* = 2.11 Hz), 5.14 (2H, s), 4.91 (2H, s), 2.12 (3H, s). ^13^C NMR (100.6 MHz, CDCl_3_) δ 166.3, 165.2, 164.2, 146.3, 138.0, 135.4, 133.1, 130.4, 130.3, 128.9, 127.9, 122.9, 115.4, 115.2, 114.5, 114.3, 101.7, 95.9, 69.1, 46.0, 20.5. ^19^ F NMR (564 MHz, CDCl_3_) δ −112.32 (1F, s). MS (ESI) *m*/*z* 358.8 [M+H]^+^ **.**

##### (6‐Methyl‐1,3‐Bis(4‐(Trifluoromethyl)Benzyl)‐4‐((4‐(Trifluoromethyl)Benzyl)Oxy)pyridin‐2(1H)‐One) (18a)

Yield%: 14%. ^1^H NMR (400 MHz, CDCl_3_) δ 7.66 (d, *J* = 8.35 Hz, 2H), 7.59 (d, *J* = 7.76 Hz, 2H), 7.50–7.40 (m, 6H), 7.27 (d, *J* = 7.76 Hz, 2H), 5.99 (s, 1H), 5.41 (s, 2H), 5.19 (s, 2H), 4.05 (s, 2H), 2.29 (s, 3H). ^13^C NMR (100.6 MHz, CDCl_3_) δ 164.2, 162.4, 145.5, 140.7, 139.8, 131.0 (q, *J*_C–F_ = 33.02 Hz), 129.8 (q, *J*_C–F_ = 31.64 Hz), 129.0, 128.0 (q, *J*_C–F_ = 31.64 Hz), 127.2, 126.7, 125.9, 125.8, 125.8, 125.7, 124.0 (q, *J*_C–F_ = 273.21 Hz), 123.9 (q, *J*_C–F_ = 273.21 Hz), 123.1, 111.2, 96.4, 69.4, 47.1, 29.8, 21.1. ^19^ F NMR (564 MHz, CDCl_3_) δ −62.56 (3F, s), −62.49 (3F, s), −62.14 (3F, s). MS (ESI) *m*/*z* 600.1 [M+H]^+^, 622.1 [M+Na]^+^.

##### (1‐(4‐Chlorobenzyl)‐3‐(3‐Fluorobenzyl)‐4‐((3‐Fluorobenzyl)Oxy)‐6‐Methylpyridin‐2(1H)‐One) (18b)

Yield%: 15%.^1^H NMR (400 MHz, CDCl_3_) δ 7.27–7.21 (1H, m), 7.17 (2H, d, *J* = 8.17 Hz), 7.11–7.01 (2H, m), 6.98 (2H, d, *J* = 8.17 Hz), 6.94–6.87 (4H, m), 6.76–6.72 (1H, m), 5.84 (1H, s), 5.19 (2H, s), 4.98 (2H, s), 3.87 (2H, s), 2.14 (3H, s). ^13^C NMR (100.6 MHz, CDCl_3_) δ 163.2, 163.1, 163.0, 161.3, 160.7, 160.6, 144.3, 142.9, 142.8, 137.4, 137.3, 134.2, 132.1, 129.3, 129.2, 128.4, 128.3, 127.9, 126.9, 123.4, 121.5, 121.5, 114.5, 114.3, 114.1, 113.1, 112.9, 111.5, 111.3, 110.2, 95.4, 68.3, 45.8, 28.5, 20.0. ^19^F NMR (564 MHz, CDCl_3_) δ −114.16 (1F, s), −112.13 (1F, s). MS (ESI) *m*/*z* 466.1 [M+H]^+^ **.**

##### In Vitro Metabolic Stability

MLM (pooled male) were obtained from Xenotech, LLC (Cambridge, Kansas City, USA). Glucose‐6‐phosphate (G6P), oxidized nicotinamide‐adenine‐dinucleotide phosphate (NADP^+^), magnesium chloride (MgCl_2_), and glucose‐6‐phosphate‐dehydrogenase (G6PDH) were supplied by Sigma‐Aldrich (Milan, Italy). Metabolic stability assays were modified from Ferlenghi et al.^[^ ^33^ ^]^ Briefly, samples were prepared in 100 mM phosphate buffered saline (PBS), pH 7.4, containing 1 mg ml^–1^ MLM and a NADPH‐generating system (5 mM MgCl_2_, 10 mM glucose‐6‐phosphate, 2 mM NADP^+^, 1.5 U ml^–1^ of glucose‐6‐phosphate dehydrogenase). After incubation (37 °C, 5 min stirring), compounds stock solutions in DMSO were added to a final concentration of 10 µM. The final DMSO percentage in samples was kept at 0.5%. After 60 min, single aliquots of reaction mixture were withdrawn, added of two volumes of acetonitrile, centrifuged (10,000 g, 10 min) and the supernatant was analyzed by HPLC. The unspecific binding to MLM was evaluated by means of control samples in which the compounds were incubated in the absence of a NADPH generating system. The apparatus used for HPLC analysis was a Shimadzu instrument, with a reverse‐phase Nova‐Pak column (Waters) (C18 4 μm 3.9 × 150 mm, Waters). The mobile phase, pumped at a flow rate of 1.2 ml min^–1^, consisted of water and acetonitrile in either a 50:50 (v/v) ratio (compounds **5c**, **6a**, **6b**, **6c**, **7**, **8**, **12**, **13**, **14**, **17b**) or a 40:60 (v/v) ratio (compounds **11** and **15**) or a 30:70 (v/v) ratio (for compounds **17a**, **18a**, **18b**). For compounds **9** and **10**, a phosphate buffer (5.98 g L^–1^ Na_2_HPO_4_, 0.19 g L^–1^ KH_2_PO_4_, adjusted to pH 7.4 with 85% H_3_PO_4_ and supplemented with 1.4 mL L^–1^ triethylamine; final pH: 8) and acetonitrile (50:50, v/v) were pumped at a flow rate of 2 mL min^–1^. In all cases, column temperature was 35 °C, injection volume was 50 µl, and absorbance was monitored at 254 nm. Serial dilutions for the calibration curves were prepared in the interval 0.5–20 µM in either PBS:CH_3_CN or H_2_O:CH_3_CN mixture (50:50, v/v) for compounds **5c**, **6a**, **6b**, **8**, **9**, **10**, **11**, **12**, **13**, **14**, **15**, **17a**, **17b**, **18a**, **18b,** and in acetonitrile for compounds **6c** and **7**.

##### Biology

**Cell Culture Maintenance:** To conduct the in vitro testing of the selected compounds 2, 5, the human hepatocarcinoma cell line HepG2 has been used. The cells have been maintained in Modified Eagle's Medium (MEM) medium supplemented with 10% FBS, 1% penicillin/streptomycin, 1% sodium pyruvate, 1% nonessential amino acids, 1% L‐glutamine (complete medium), and incubated at 37 °C and 5% CO_2_.

##### Compounds Preparation for In Vitro Testing

The compounds were dissolved in DMSO to obtain a 80 mM stock solution. Treatments were prepared in MEM medium supplemented with 0.4% FBS, 1% penicillin/streptomycin, 1% sodium pyruvate, 1% nonessential amino acids, 1% L‐glutamine (starving medium). Simvastatin was prepared in physiologic solution according to manufacturer instruction. BBR was dissolved in DMSO according to manufacturer instructions. Hepatocellular carcinoma HepG2 cell line was cultured in a 75 cm^2^ adhesion flask in MEM supplemented with 10% v/v FBS, penicillin/streptomycin (10000 U ml^–1^ and 10 mg ml^–1^, respectively, at a final concentration of 1% v/v); 1% v/v L‐glutamine [200 mM], 1% v/v Na‐pyruvate [100 mM], 1% v/v nonessential amino acids. Cells were maintained at 37 °C, 95% humidity and 5% CO_2_ and subcultured at 80% confluence. All plastic materials were purchased from VWR International (Radnor, Pennsylvania, USA), cell culture medium and FBS from Euroclone (Milan, Italy) and supplemental reagents from Thermo Fisher Scientific (Waltham, Massachusetts, USA).

##### Cell Viability Assay

Cell viability was assessed using the MTT assay. Cells were seeded in 96‐well plates (2.5 × 10^4^ cells/well) in MEM supplemented with 10% v/v FBS and incubated for 24 h. The cells were then treated with the synthesized compounds dissolved in DMSO at increasing concentrations (from 0.5 to 50 µM) in MEM supplemented with 0.4% v/v FBS for 24 h. Simvastatin [20 and 40 µM] and BBR [10 µM] (both from Merck, St. Louis, Missouri, USA) were used as internal controls as PCSK9 inducer and inhibitor, respectively.^[^ ^21^ ^,^ ^34^ ^,^ ^35^ ^]^ The cells were then incubated for 2 h with a solution of MTT [1 mg mL^–1^] in MEM supplemented with 0.4% v/v FBS, away from light sources. The supernatant was then collected and 200 µL of DMSO was added to each well to solubilize the newly formed formazan. Finally, an aliquot of 100 µL was taken to measure the absorbance at a wavelength of 570 nm using an absorbance microplate reader (Spark Tecan, Switzerland). Every experimental condition was run in duplicate and the average cell viability and standard deviation was calculated. Cell viability was expressed as the percentage of treated cells compared to untreated (basal) cells. CC_50_ values for each compound were calculated by interpolating the percentage of cell viability through nonlinear regression analysis of the log (inhibition) versus response using GraphPad Prism software version 8.0 (GraphPad Software Inc., La Jolla, CA).

##### Evaluation of PCSK9 Secretion in the Culture Medium

The amount of PCSK9 secreted into the culture media was measured with an appropriate ELISA kit (R&D System, DY3888 and DY008B2) on the supernatants collected during the MTT assay. Every experimental condition was run in duplicate and the average PCSK9 secretion and standard deviation was calculated. A 4PL regression analysis was used to interpolate unknowns with the standard curve using GraphPad Prism v10.2, according to manufacturers’ instructions. The data were normalized onto the corresponding cell viability, and IC_50_ was then calculated using GraphPad Prism v10.2.

##### Evaluation of PCSK9 and LDLR Protein Expression

HepG2 cells were seeded at the cellular density of 500,000 cells/well in a 6‐well tray, in 2 mL of complete medium, and incubated as described above. The day after, treatments were prepared in starving medium. The cells were washed once with PBS and then incubated with media containing treatments. Compounds were tested at the nontoxic concentrations of 2, 2.5, 5, and10 μM. Simvastatin and BBR at the concentration of 40 μM were used as negative and positive controls for PCSK9 expression, respectively. The appropriate amount of DMSO was added in the control condition as well as in the simvastatin condition. Where needed, the % DMSO was adjusted to normalize all the tested conditions. The treatments lasted 24 h, upon which conditioned media were discarded and the cell layers washed 2X with cold PBS to remove any media residue. The cells were then lysed on ice with a nonionic NP‐40‐based lysis buffer made‐up according to Abcam's recipe, moved into prechilled vials and incubated on ice for 30 min, after which the vials were centrifuged at 20,000 g, 4 °C, for 10 min. The protein‐containing supernatants were collected, and the debris‐rich pellets were discarded. Total protein content was measured through bicinchoninic acid (BCA) assay (Euroclone, EMP014250), according to manufacturer's instructions. When necessary, samples were diluted in lysis buffer, then Laemmli buffer 4X (Bio‐Rad recipe) was added, and samples boiled for 5 min to reach the complete denaturation of the proteins. A minimum of 20 μg of proteins were used for the electrophoretic run (Bio‐Rad apparatus) on 4%–20% SDS‐PAGE gels (Bio‐Rad, 4,561,094). TransBlot Turbo (Bio‐Rad) was used for the semi‐dry transfer of the proteins to a 0.22 μM nitrocellulose membrane (Bio‐Rad, 1,704,158). Red Ponceau S was used to evaluate the transfer. Membranes were then blocked 1 h at room temperature with 5% w/v nonfat dry milk in TBS‐Tween 20 1X, upon which they were incubated o/n with primary antibodies diluted in blocking solution and specific for LDLR (1:1000, GeneTex, GTX132860), PCSK9 (1:1000, GeneTex, GTX129859), and GAPDH (1:10000, GeneTex, GTX100118). The day after, primary antibodies were discarded and membranes were washed three times at room temperature with TBS‐Tween 20 1X, 15 min each wash. Membranes were then incubated at room temperature with anti‐rabbit secondary antibody (1:5000, Jackson ImmunoResearch, 111‐036045) for 90 min and immediately washed three times with TBS‐Tween 20 1X as before. Immunobands were detected with Imager Alliance Q9 ATOM Supreme by UVITEC (Cambridge, UK) after 5 min of membranes incubation in the dark with ECL Star solution (EuroClone, EMP001005). Every experimental condition was run in triplicate and the average cell viability and standard deviation was calculated.

##### Evaluation of PCSK9 mRNA Expression

Total RNA was extracted using the iScript RT‐qPCR Sample Prep reagent (Bio‐Rad), according to the manufacturer's instructions. QuantiNova SYBR Green RT‐PCR Kit (QIAGEN, Hilden, Germany) was used for qPCR, along with specific primers for 18S (FWD 5′‐CGGCTACCACATCCACGGAA‐3′, REV 5′‐CCTGAATTGTTATTTTTCGTCACTACC‐3′) PCSK9 (FWD 5′‐CCTGCGCGTGCTCAACT‐3′, REV 5′‐GCTGGCTTTTCCGAATAAACTC‐3′). The analyses were performed with the CFX96 Touch Real‐Time PCR Detection System (Bio‐Rad) with cycling conditions of 45 °C for 10 min, 95 °C for 5 min, and a repetition of 40 cycles at 95 °C for 5 s followed by 30 s at 60 °C. The data were expressed as Ct values and used for relative quantification of targets with ΔΔCt calculations. The ΔΔCt values were determined by multiplying the ratio value between the efficiency of specific primers and housekeeping 18S. The efficiency was calculated as ((10(‐1/slope))−1) × 100.

##### Statistical Analysis

Statistical analysis was performed using the Prism statistical analysis package (GraphPad Software, San Diego, CA, USA). When possible, *p* values were determined by Student's t test. Otherwise, differences between treatment groups were evaluated by one‐way ANOVA. A probability value of *p* < 0.05 was considered statistically significant.

## Conflict of Interest

The authors declare no conflict of interest.

## Supporting information

Supplementary Material

## Data Availability

The data that support the findings of this study are available in the supplementary material of this article.
