# Supplementary material for: Optimization of 4‐Amino‐2‐Pyridone Inhibitors of Proprotein Convertase Subtilisin/Kexin Type 9: Integrating Structure–Activity and Structure–Metabolism Relationships
Source: ChemMedChem. 2025 Nov 28;21(1):e202500651. doi: 10.1002/cmdc.202500651 (PMC12812009; doi:10.1002/cmdc.202500651)

Supporting Information

Optimization of 4-Amino-2-Pyridone Inhibitors of Proprotein Convertase Subtilisin/Kexin type 9 (PCSK9): Integrating Structure-Activity and Structure-Metabolism Relationships

Lisa Giannessi,[a,1] Maria Giovanna Lupo,[b,1] Martina Ugolotti,[a] Bianca Papotti,[a] Beatrice Mattina,[a] Maria Grazia Martina,[a] Anna Demurtas,[a] Cristina Padula,[a] Sara Nicoli,[a] Marco Crescenzo,[b] Nicola Ferri,[b,*] Francesca Zimetti[a,*] and Marco Radi [a,*]

[a] Dipartimento di Scienze degli Alimenti e del Farmaco (DipALIFAR), Università degli Studi di Parma, Viale delle Scienze, 27/A, 43124 Parma, Italy

[b] Department of Medicine, University of Padova, 35128 Padova, Italy

[1] These authors equally contributed to the present work.

<u>Table of contents:</u>

Supplementary Tables S2-S3
Supplementary Figures S4
NMR Spectra S5-S31

Table S1. Optimization of reaction conditions for the synthesis of compound **11**.
During reaction optimization, microwave heating proved unsuitable as it lowered the yield of the desired product. Instead, it favored the formation of a side product, where **5c** competed with Meldrum's acid as a nucleophile, resulting in the dimeric product **23**

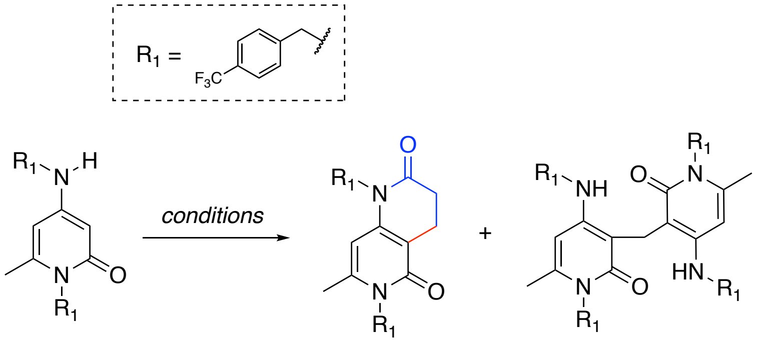

5c **11** side propduct **23**

Entry	C-nucleophile	solvent	heating	time	yield (%)
1[b]	Meldrum acid	H_2O	conventional	on	0
**2**	**Meldrum acid**	**H_2O/CH_3CN**	**conventional**	**on**	**44**
3	Meldrum acid	$H_2O/EtOH$	conventional	on	0
4	Meldrum acid	EtOH	conventional	on	0
5	Meldrum acid	CH_3CN	conventional	on	18
6[b]	Meldrum acid	H_2O	microwave	20 min	0
7[c]	Meldrum acid	H_2O/CH_3CN	microwave	20 min	10
8[c]	Meldrum acid	H_2O/CH_3CN	microwave (open vessel)	20 min	10
9[c]	Meldrum acid	$H_2O/EtOH$	microwave	20 min	0
10[c]	Meldrum acid	EtOH	microwave	20 min	0
11[c]	Meldrum acid	CH_3CN	microwave	20 min	10
12[c]	Meldrum acid	neat	microwave	20 min	0

[a]*Reaction conditions.* ***5c*** *(1.0 eq), paraformaldehyde (3.5 eq), Meldrum acid (4.0 eq), AcOH (0.2 eq), solvent (2.0 mL), under vigorous stirring at 80°C, monitored by TLC analysis. Where not otherwise specified, microwave heating was performed in a sealed tube.*
[b]*No reaction for solubility issues.*
[c]*Side product* ***23*** *formation.*

Table S2. Inhibitory potency of synthesized compounds on PCSK9 secreted by HepG2 cell line. PCSK9 levels were quantified by ELISA assay from conditioned media collected after incubation with non-cytotoxic concentrations of different compounds. Results are expressed as IC_{50}, inhibitory concentration of 50% ± standard deviation.

**Compound**	**IC_{50} ± SD (μM)**
**5c**	1.7 ± 0.67
**6a**	5.4 ± 2.65
**6b**	2.6 ± 1.61
**7**	4.1 ± 0.36
**8**	7.2 ± 0.51
**11**	10.2 ± 2.81
**12**	13.6 ± 2.67
**14**	6.4 ± 0.37

Table S3. *Experiments for the fluorination of* ***5c***. Constant parameters: 0.1 mmol scale (44.0 mg); DDQ (20 mol%); no TFA. Entry 5 corresponds to the standard conditions reported in Scheme 1

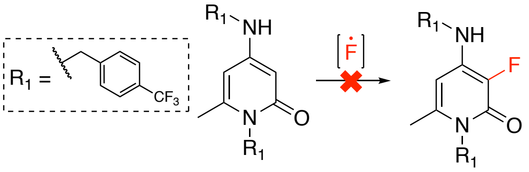

Entry	Solvent (4.0 mL)	Fluorinating reagent (2.0 eq)	Electrodes	I/V	Additive	Outcome
1	DME	Et_3N*3HF	Cgr/Cgr	10 mA	/	High resistance
2	DME	Et_3N*3HF	Cgr/Cgr	10 mA	MsOH	No reaction
3	CH_3CN	TBAF*$3H_2O$	Cgr/Pt	4.0 V	MsOH	No reaction
4	MeOH	TBAF*$3H_2O$	Cgr/Pt	-4 - +4 V; a.p.	MsOH	No reaction
5	MeOH	NaF	Cgr/Cgr	10 mA	/	No Reaction

Table S4. Elemental analysis of final compounds

Compd.	Elemental analysis (%)					
	Calculated			Found		
	C	H	N	C	H	N
**6a**	55.65	3.61	5.90	55.77	3.73	5.86
**6b**	50.89	3.30	5.39	50.96	3.35	5.29
**6c**	46.66	3.03	4.95	46.75	3.12	5.05
**7**	55.53	3.44	8.45	55.71	3.58	8.36
**8**	58.98	3.87	5.98	59.12	3.94	5.92
**9**	61.95	5.20	8.03	62.10	5.32	8.14
**10**	61.95	5.20	8.03	61.88	5.26	8.09
**11**	60.73	4.08	5.67	60.87	4.21	5.60
**12**	59.75	4.18	5.81	59.66	4.25	5.72
**13**	63.97	4.07	5.14	64.07	4.18	5.03
**14**	55.77	3.71	5.42	55.88	3.79	5.32
**15**	51.35	3.41	4.99	51.44	3.52	5.12
**17a**	59.87	3.88	3.17	59.80	3.96	3.07
**17b**	67.14	4.79	3.91	67.30	4.91	3.81
**18a**	60.11	3.70	2.34	60.20	3.77	2.38
**18b**	69.60	4.76	3.01	69.71	4.84	2.93

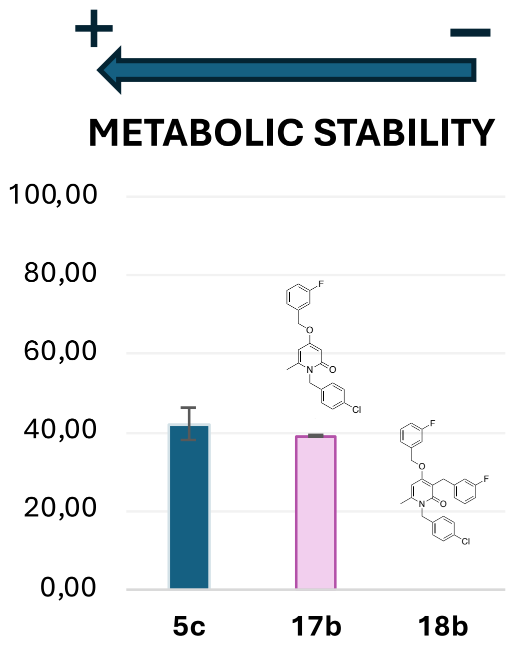

Figure S1. *Metabolic stability data of the 4-alkoxy-2-pyridone derivatives* ***17b*** *and* ***18b*** *in comparison to* ***5c****.* Derivatives were incubated with mouse liver microsomes and an NADPH-generating system for 60 minutes, followed by HPLC analysis. Control samples included compounds and liver microsomes without the NADPH system, allowing for the assessment of non-specific binding to microsomes.

NMR SPECTRA

^{1}H NMR (400 MHz, $CDCl_3$)

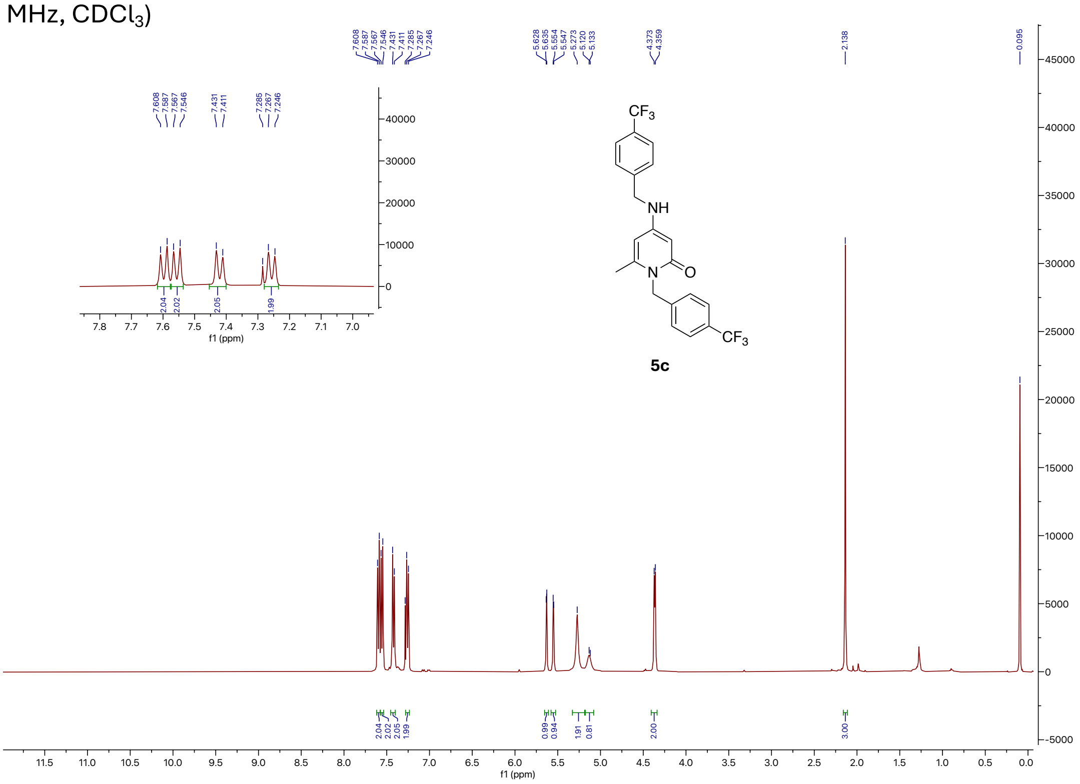

^{13}C NMR (100.6 MHz, $CDCl_3$)

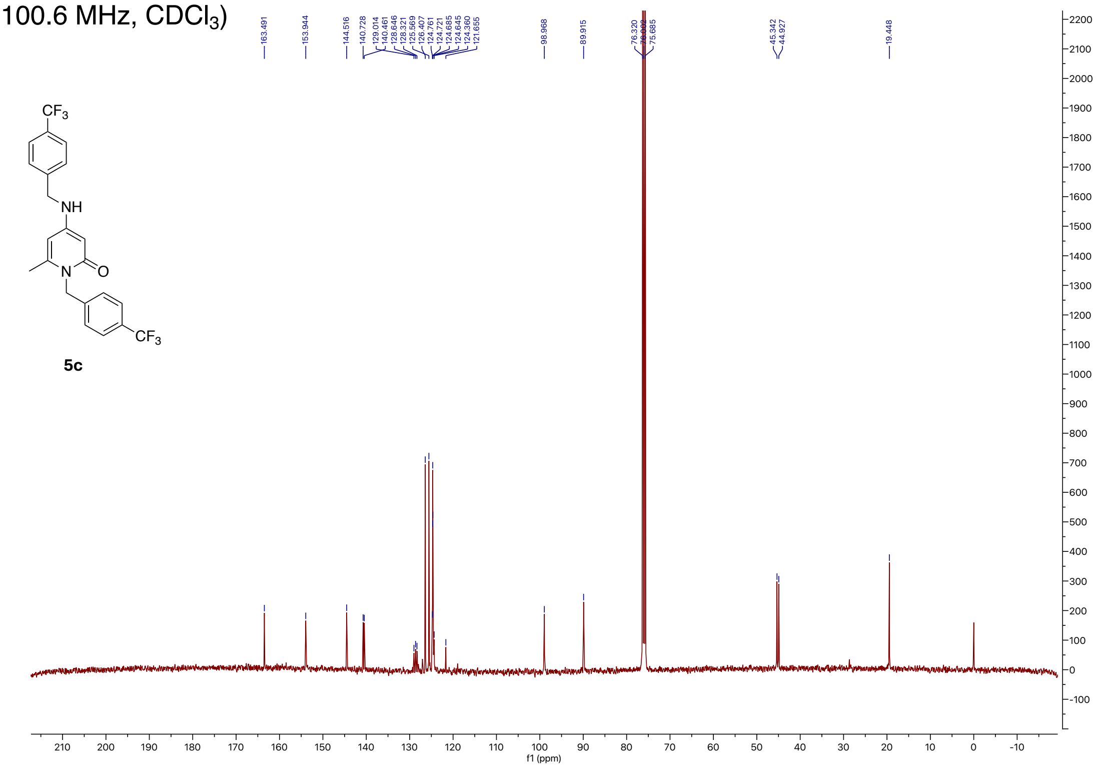

^{19}F NMR (564 MHz, $CDCl_3$)

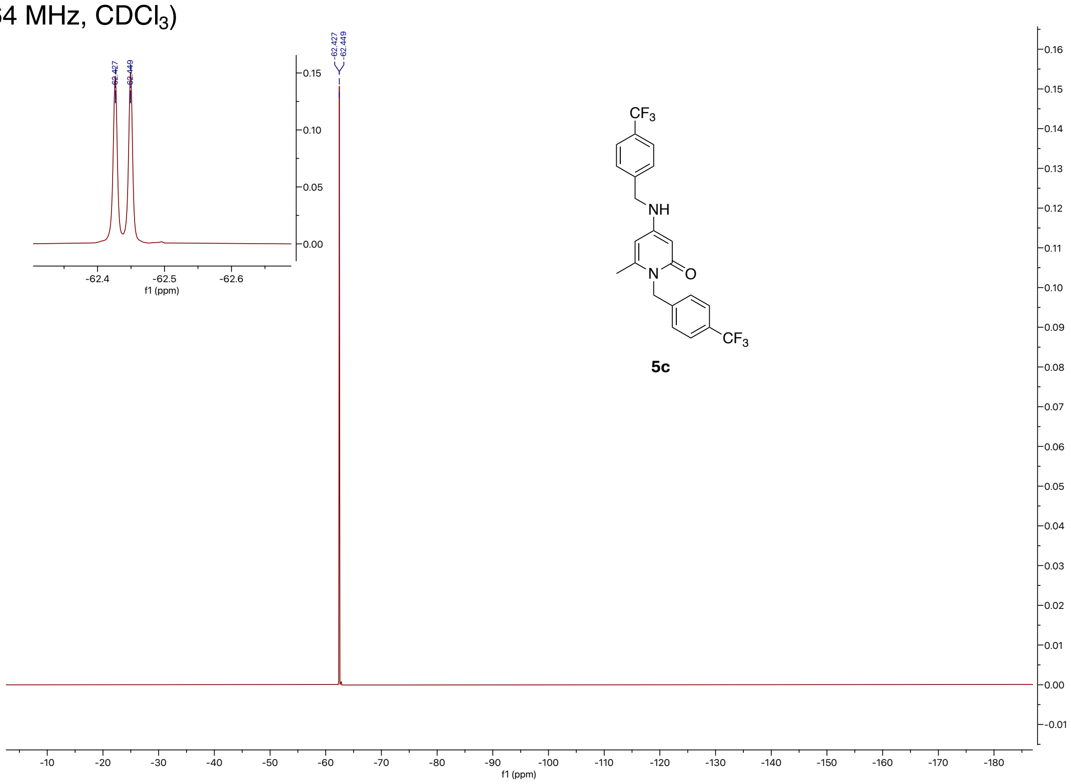

5c

^{1}H NMR (400 MHz, $CDCl_3$)

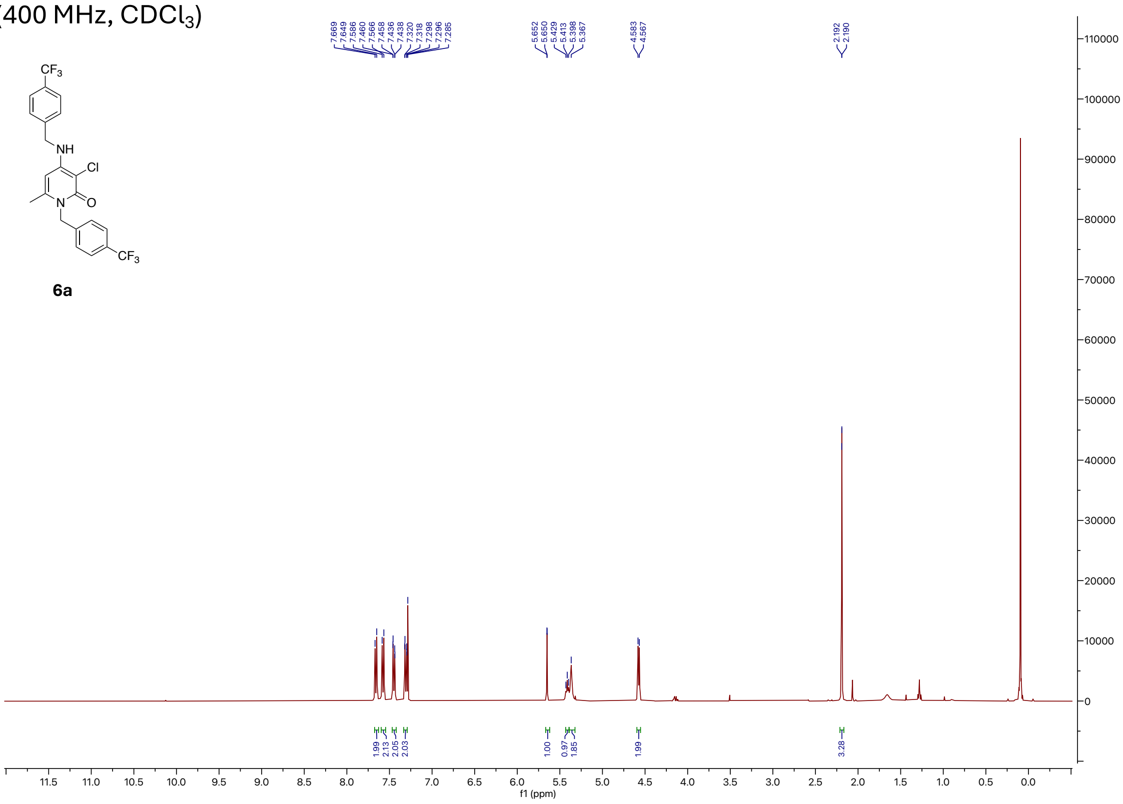

6a

^{13}C NMR (100.6 MHz, $CDCl_3$)

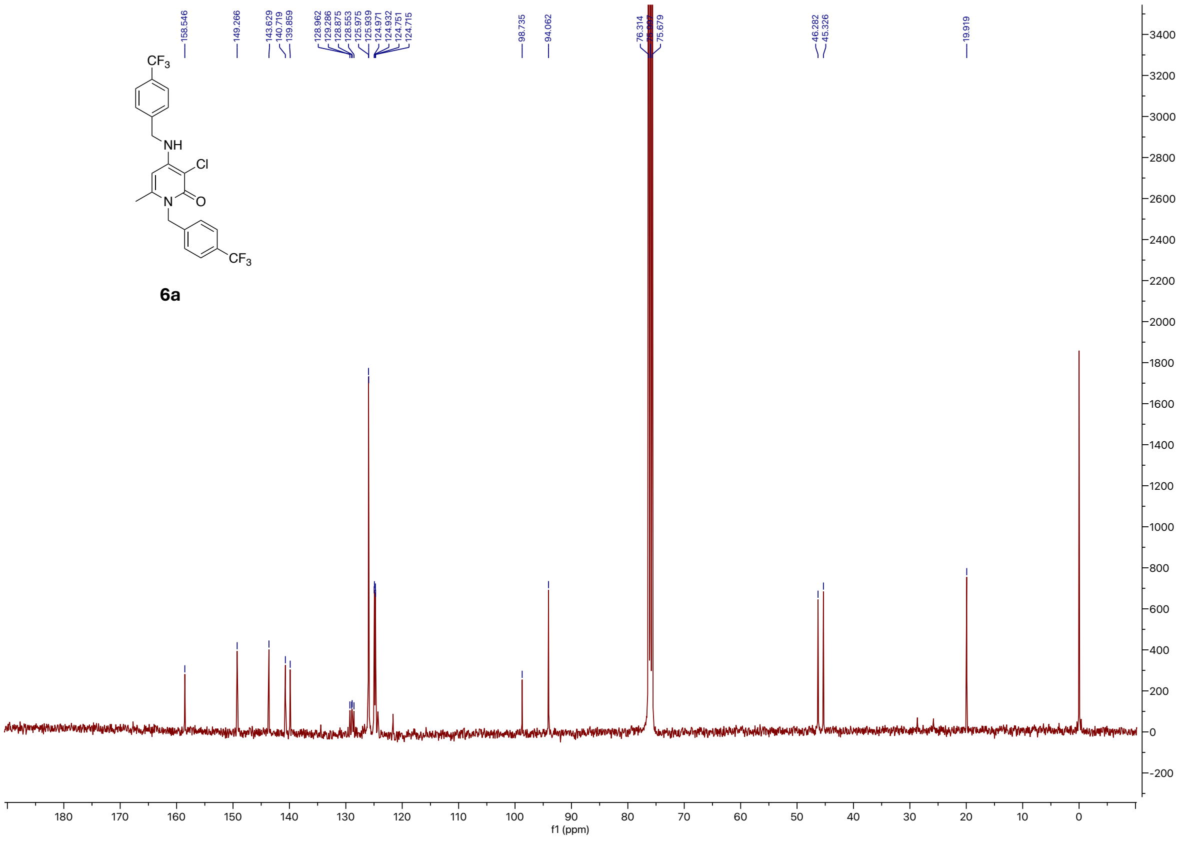

6a

^{19}F NMR (564 MHz, $CDCl_3$)

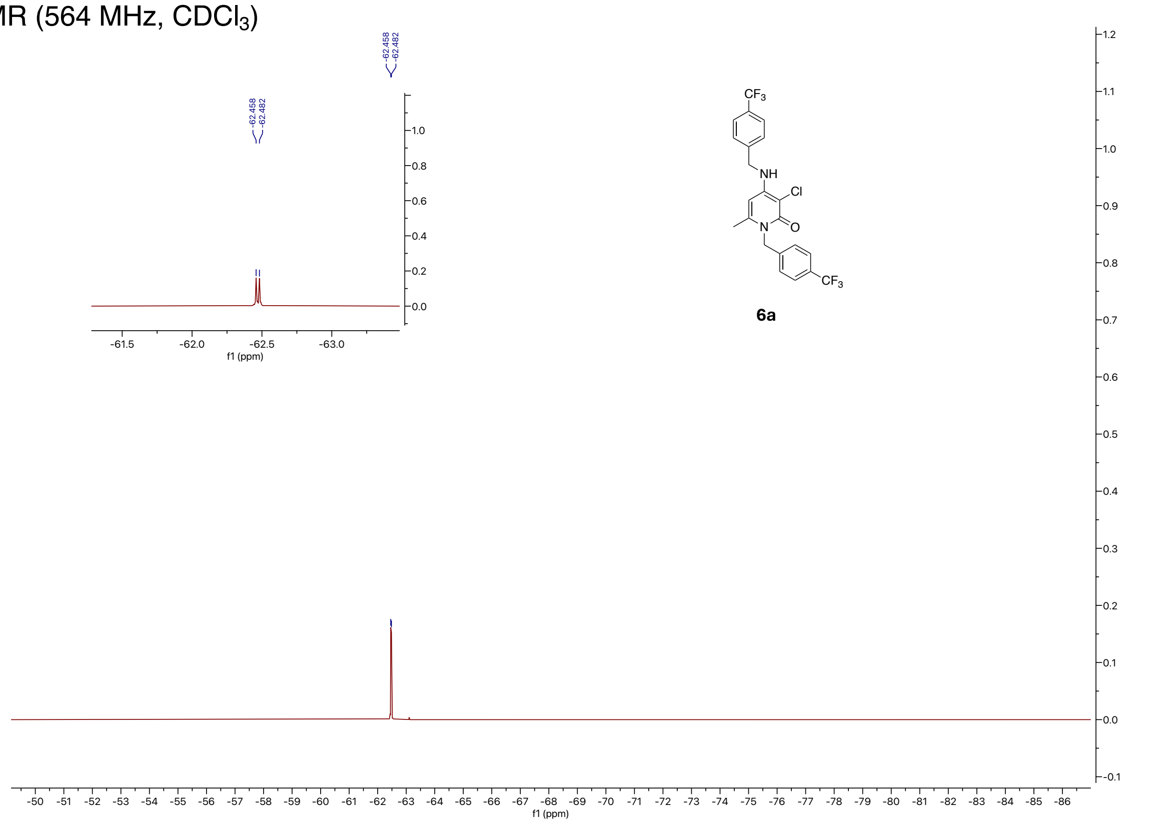

^{1}H NMR (400 MHz, $CDCl_3$)

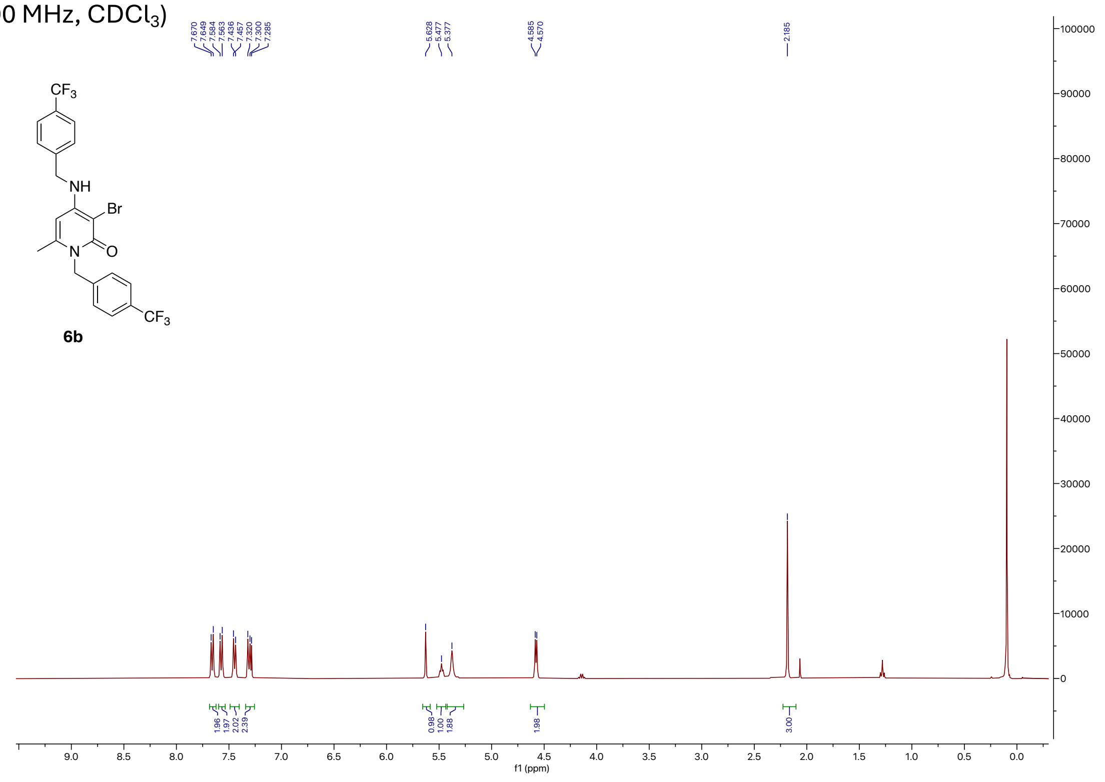

^{13}C NMR (100.6 MHz, $CDCl_3$)

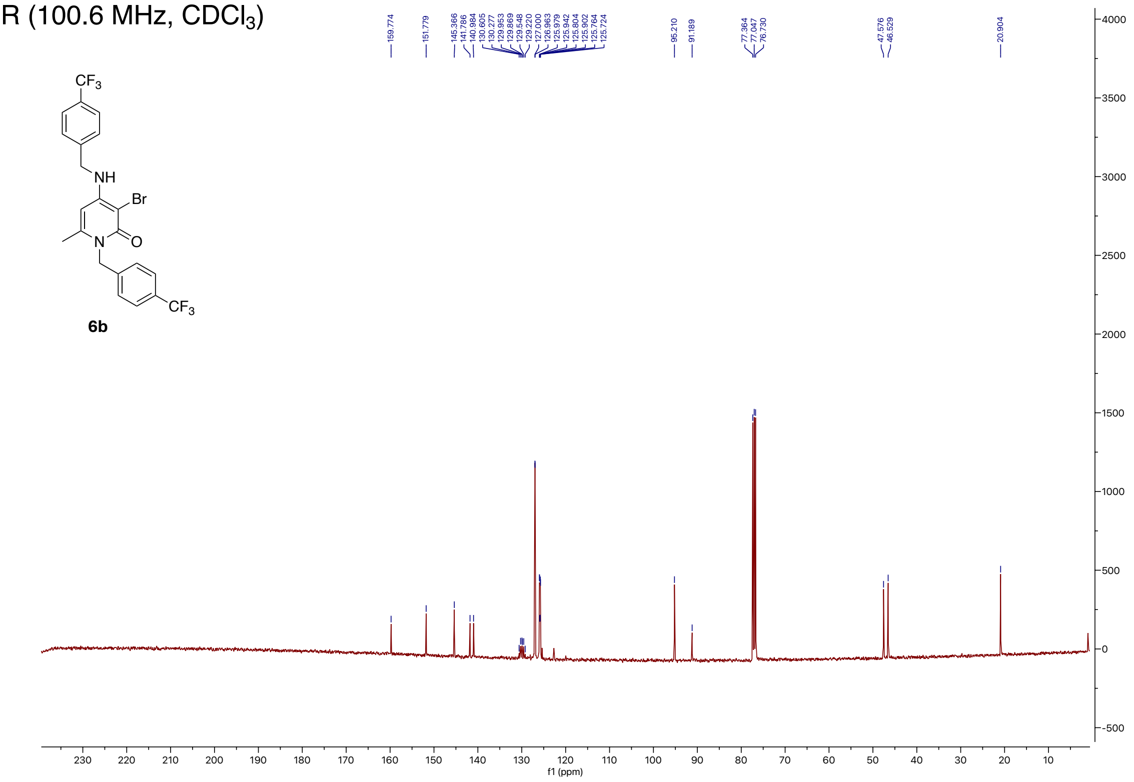

^{19}F NMR (564 MHz, $CDCl_3$)

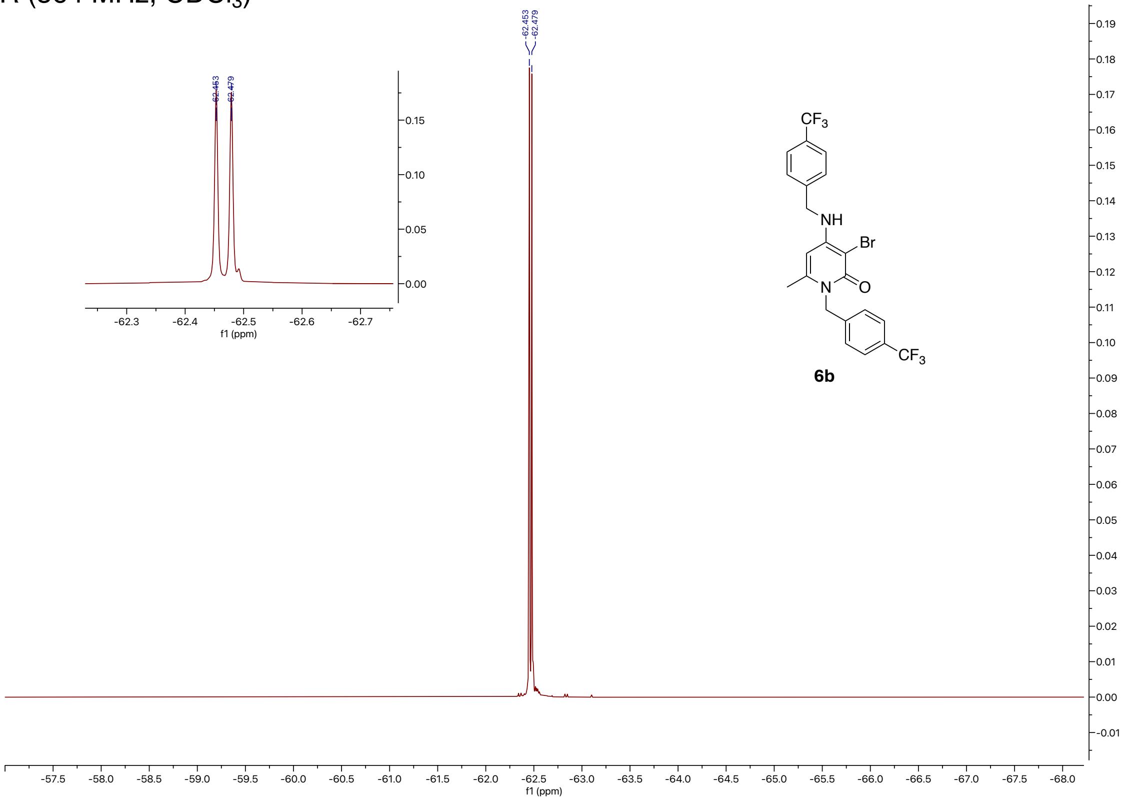

^{1}H NMR (400 MHz, $CDCl_3$)

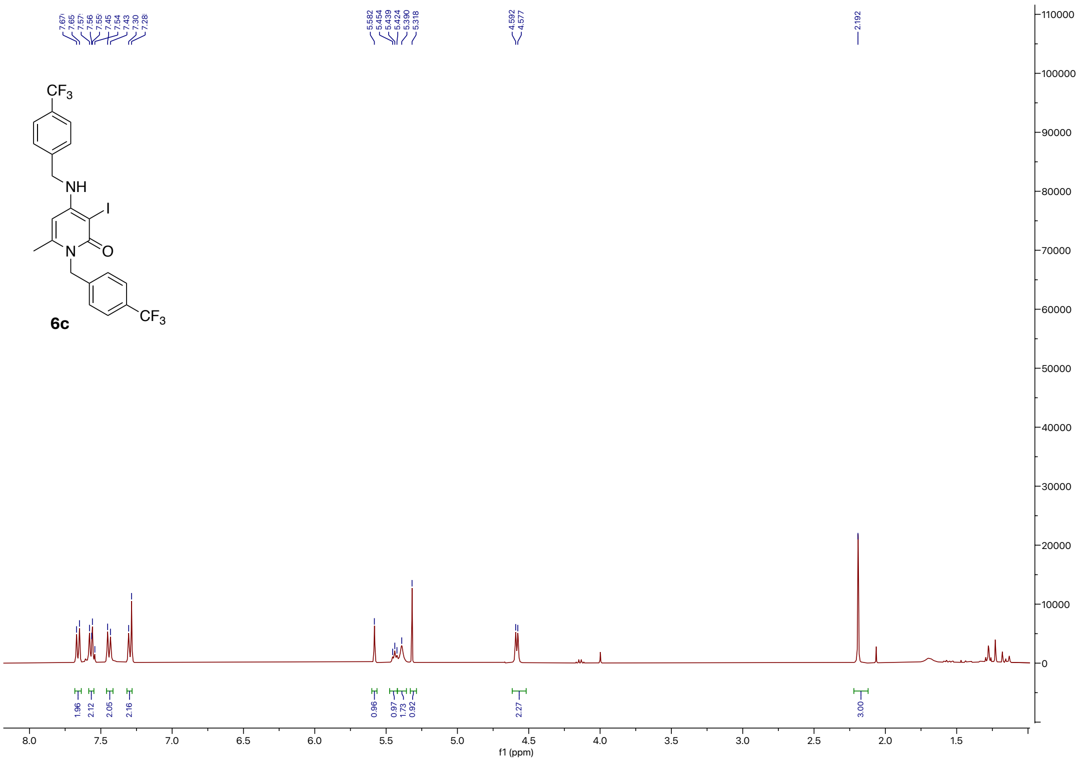

^{13}C NMR (100.6 MHz, $CDCl_3$)

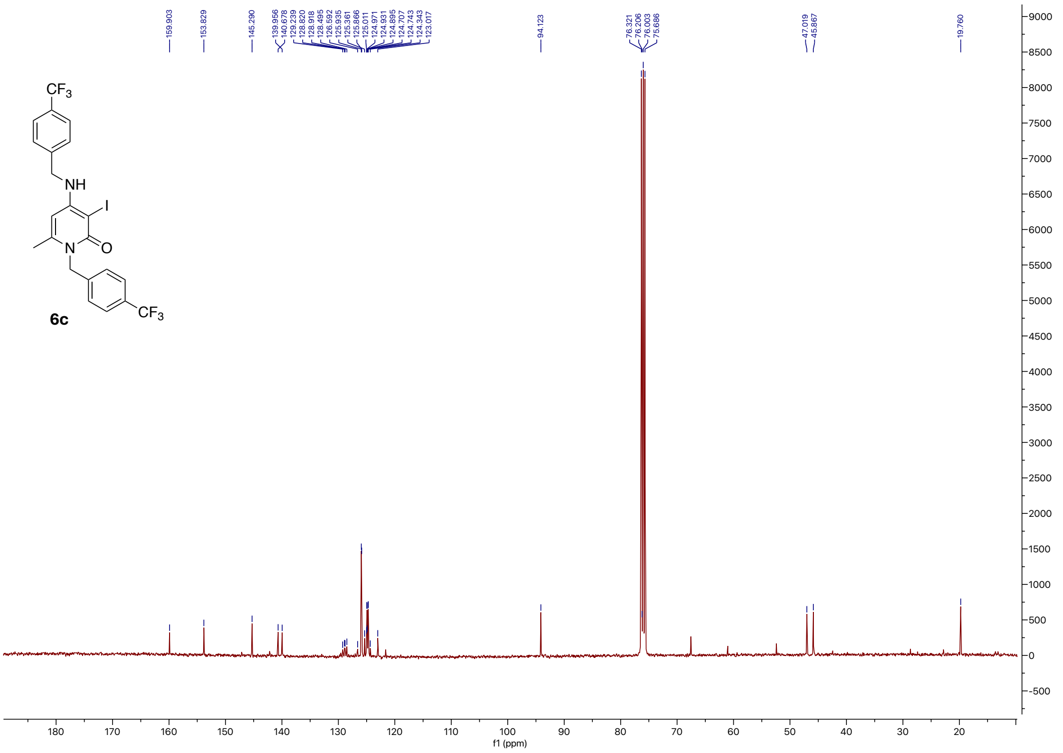

^{19}F NMR (564 MHz, $CDCl_3$)

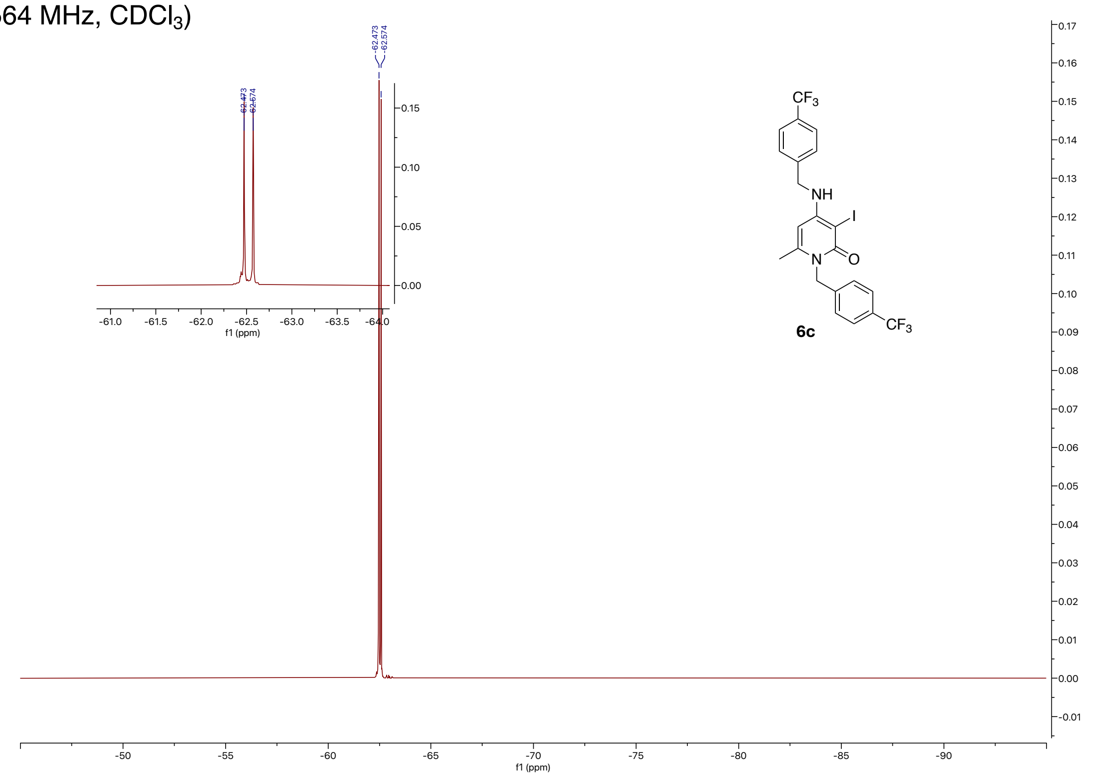

^{1}H NMR (400 MHz, DMSO)

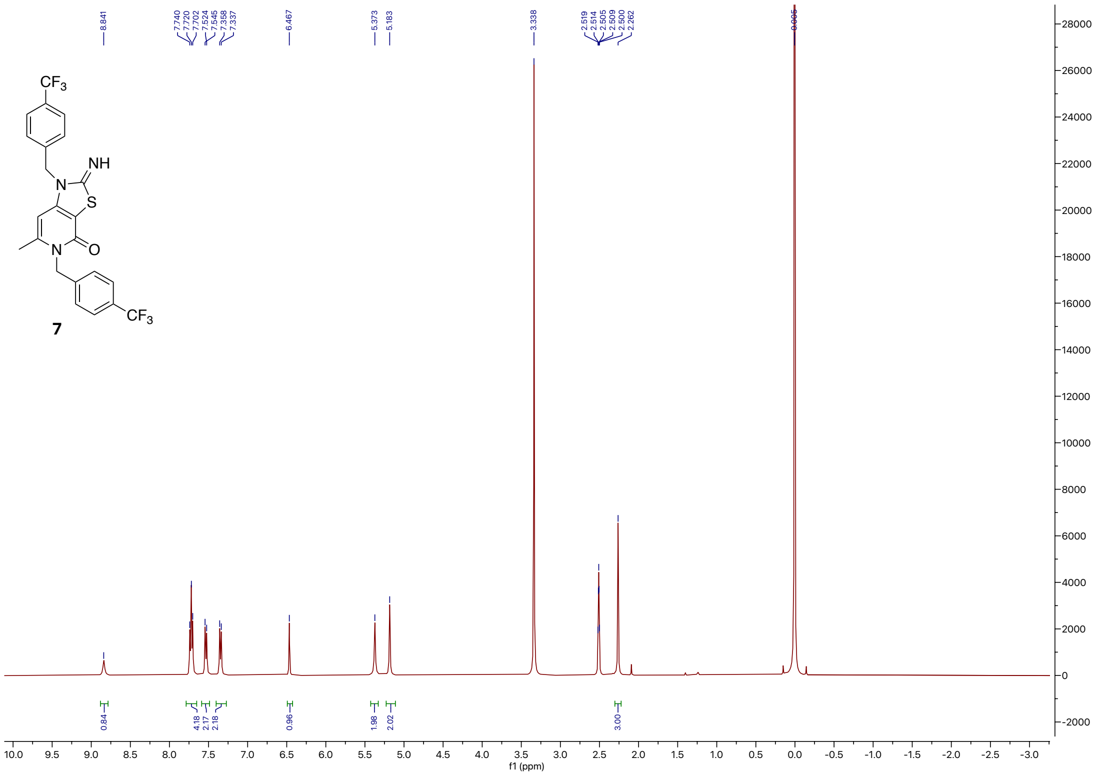

^{13}C NMR (100.6 MHz, DMSO)

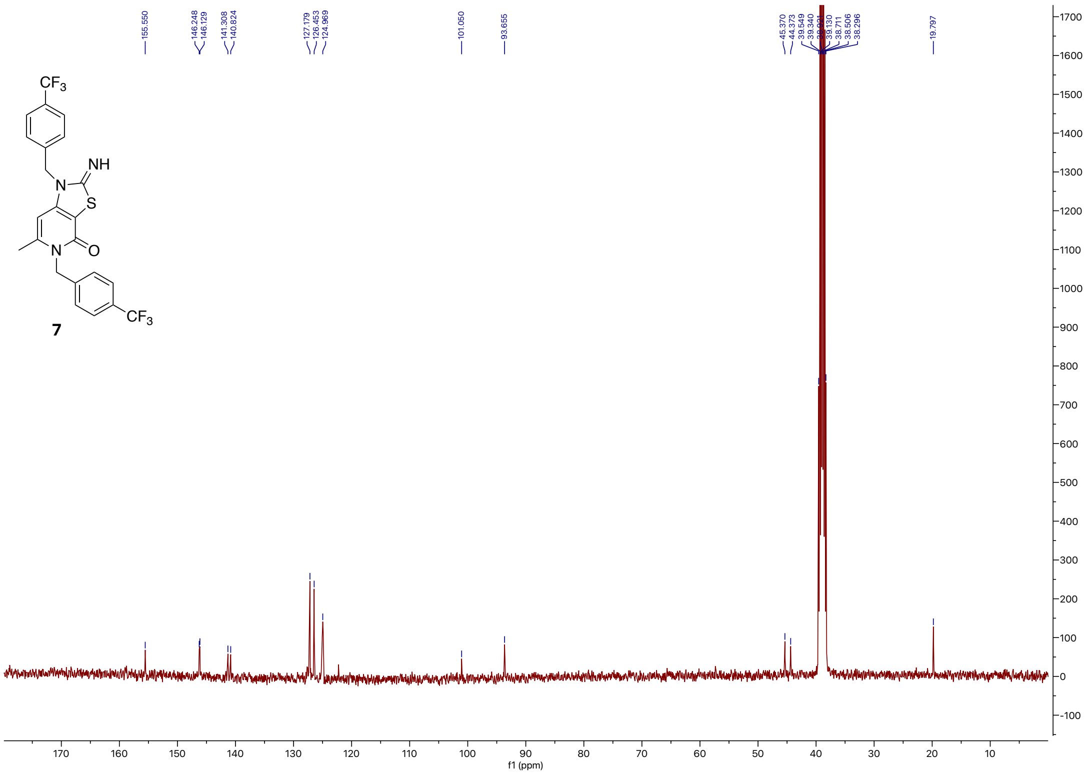

^{19}F NMR (564 MHz, $CDCl_3$)

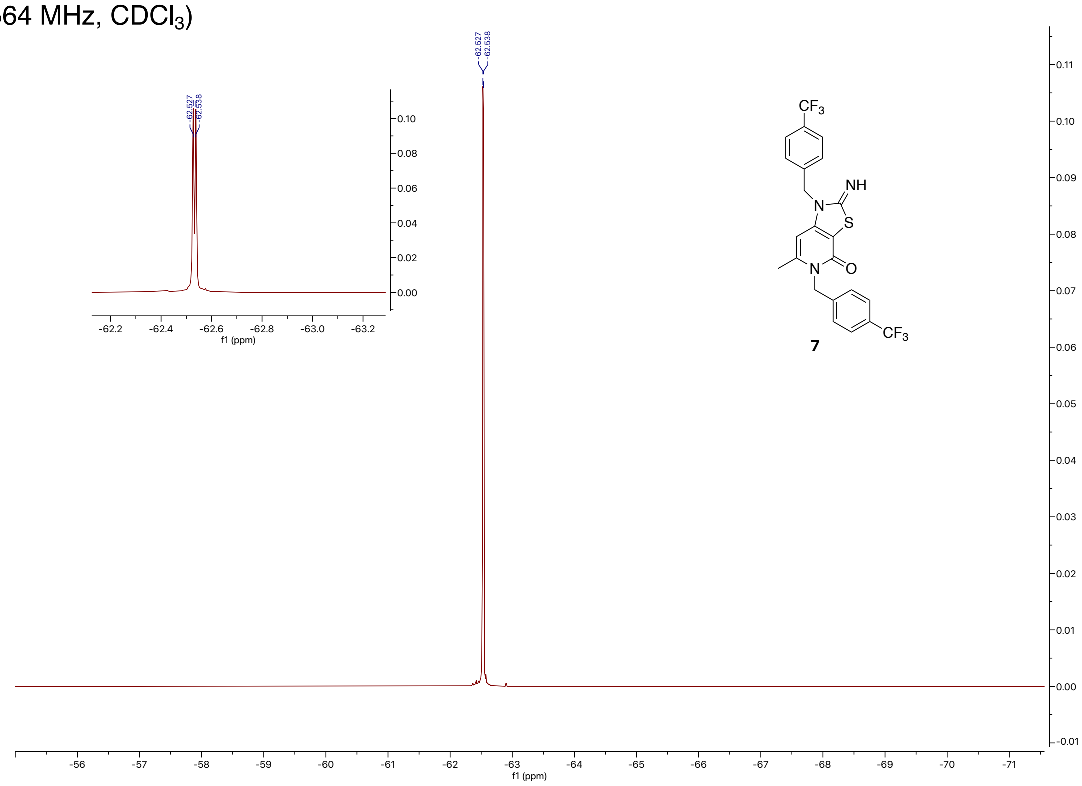

^{1}H NMR (400 MHz, $CDCl_3$)

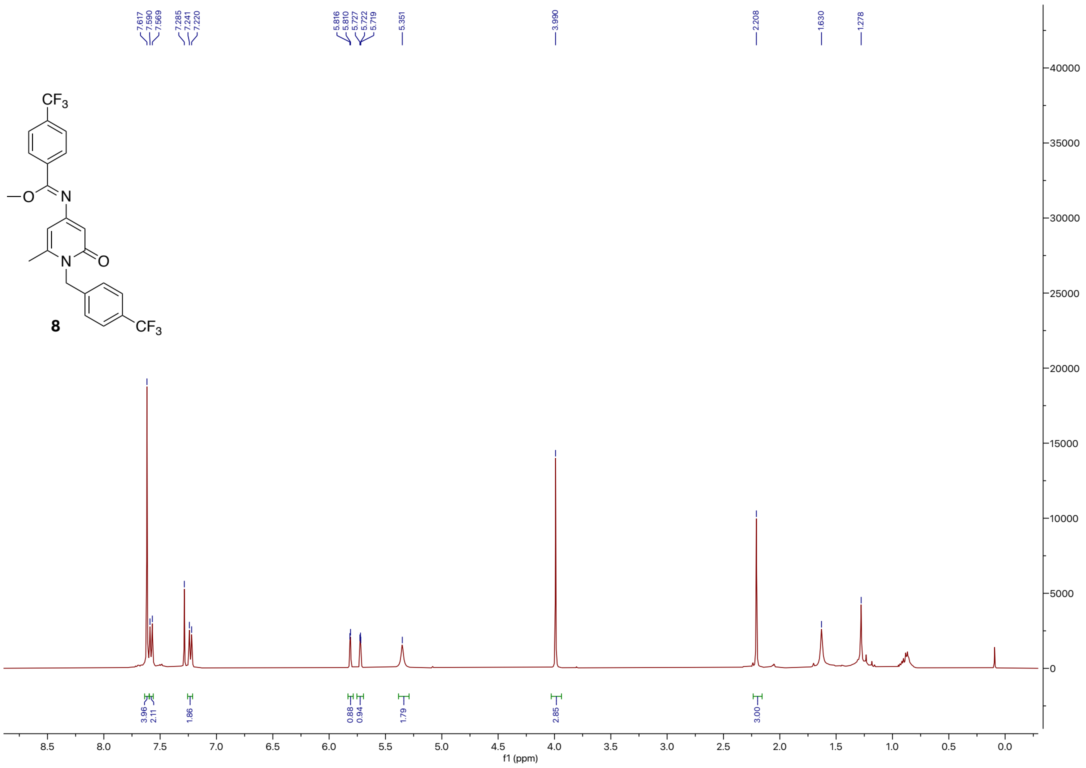

^{13}C NMR (100.6 MHz, $CDCl_3$)

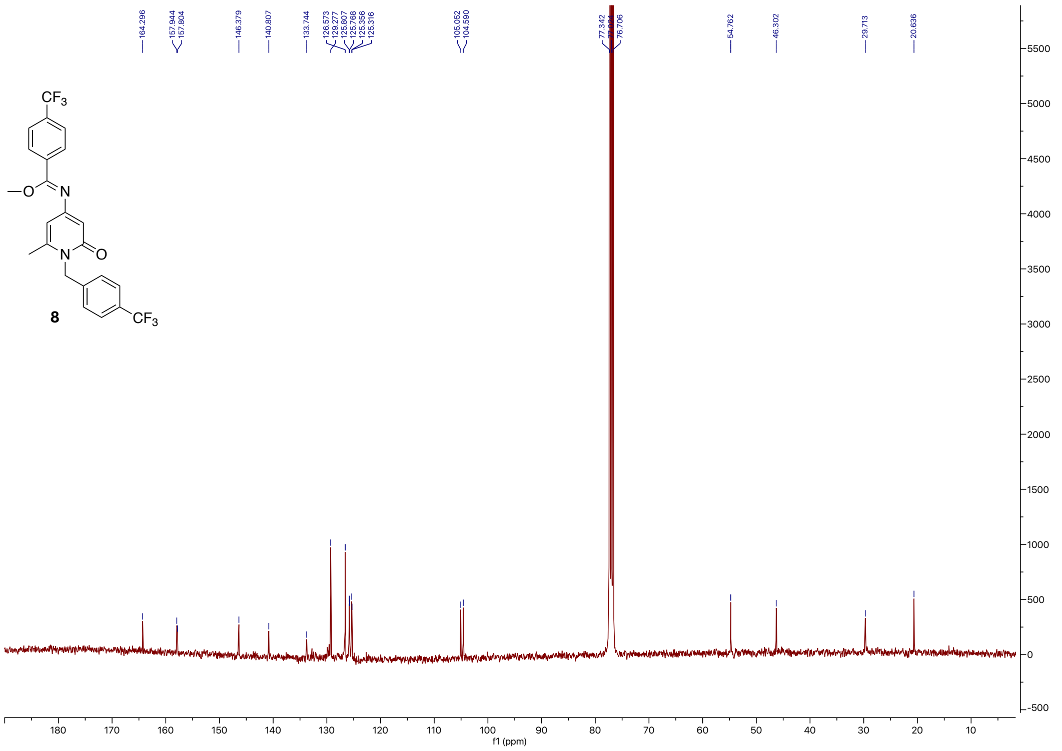

^{19}F NMR (564 MHz, $CDCl_3$)

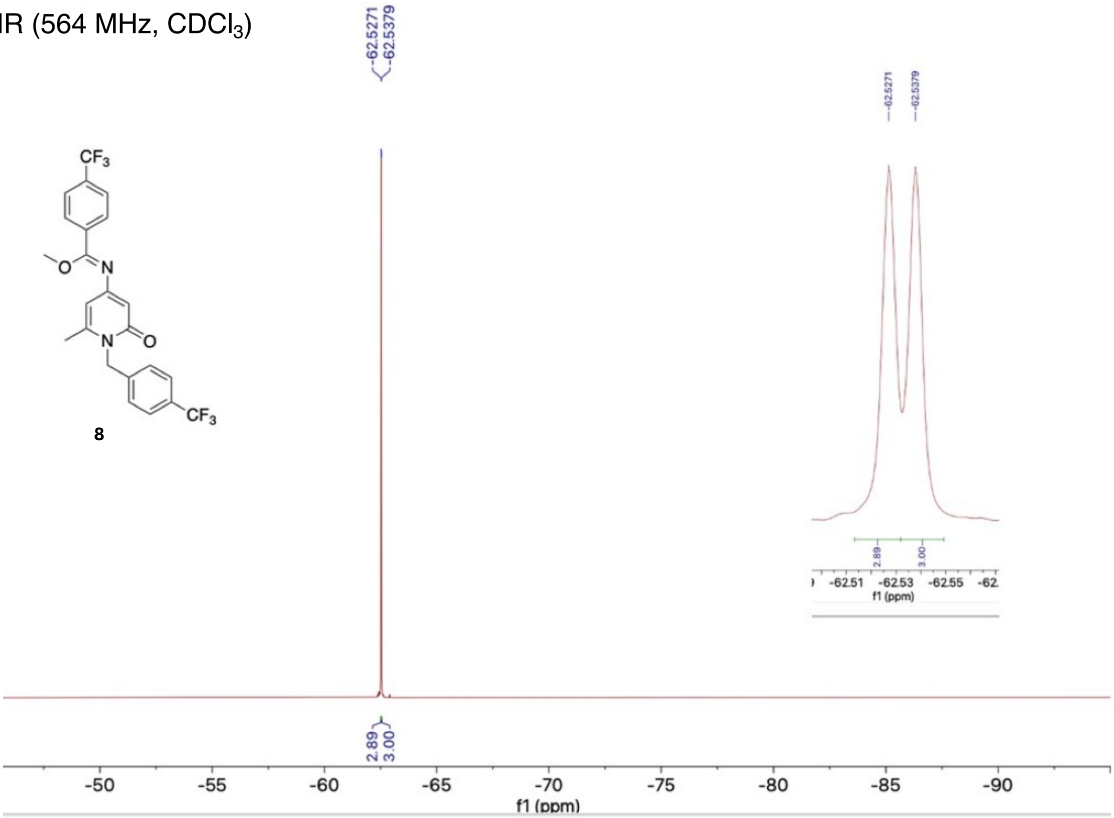

8

^{1}H NMR (400 MHz, $CDCl_3$)

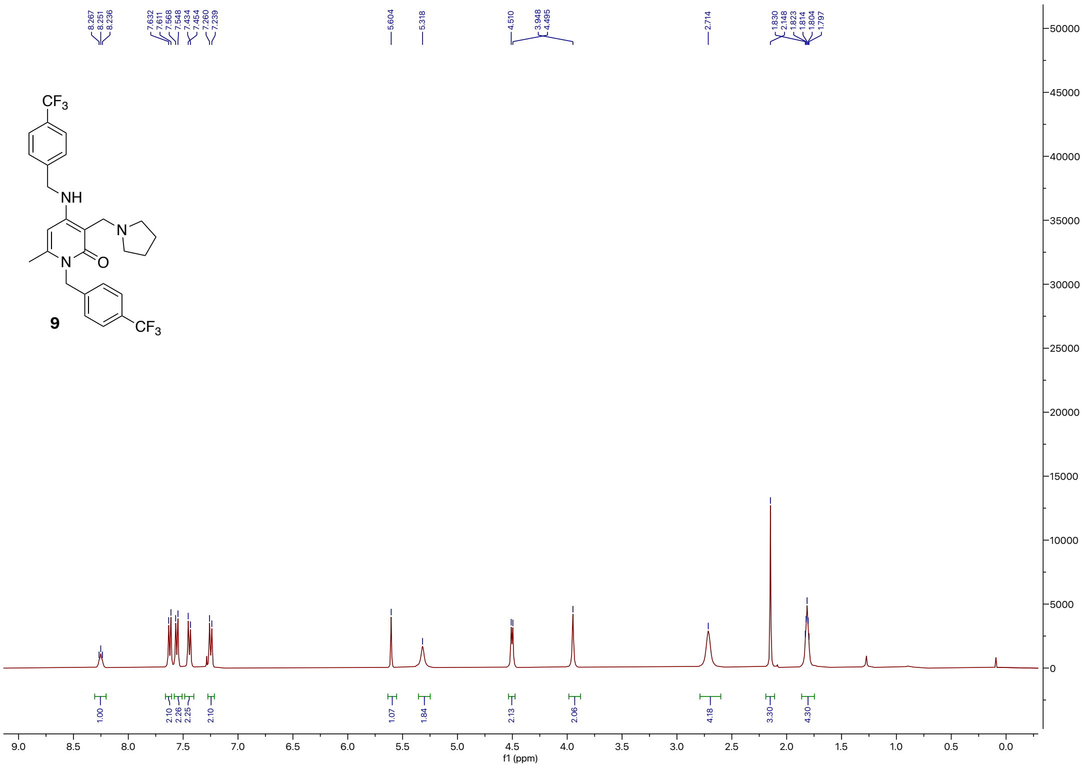

9

^{13}C NMR (100.6 MHz, $CDCl_3$)

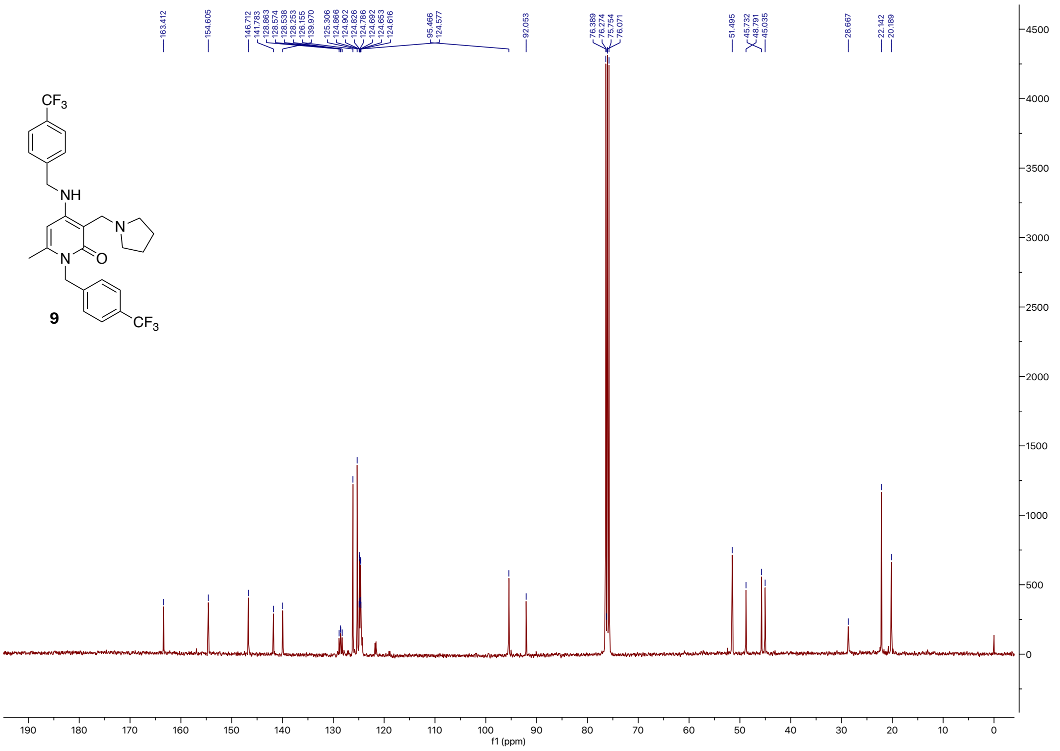

9

^{19}F NMR (564 MHz, $CDCl_3$)

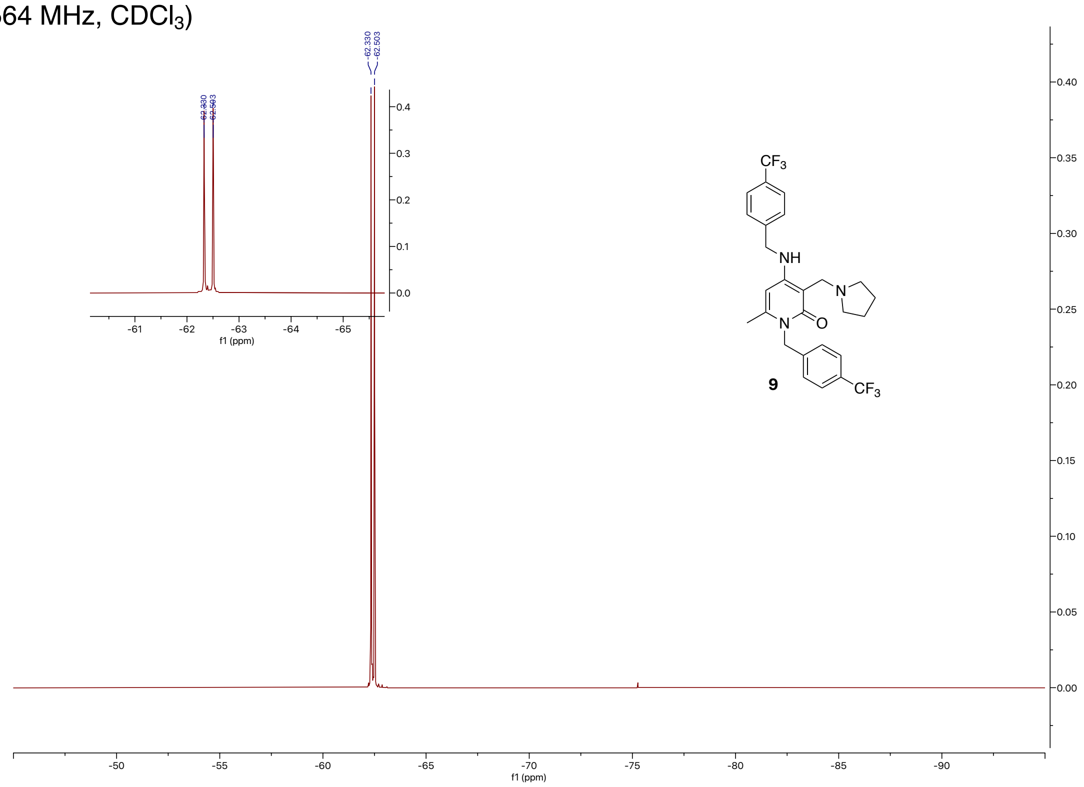

^{1}H NMR (400 MHz, $CDCl_3$)

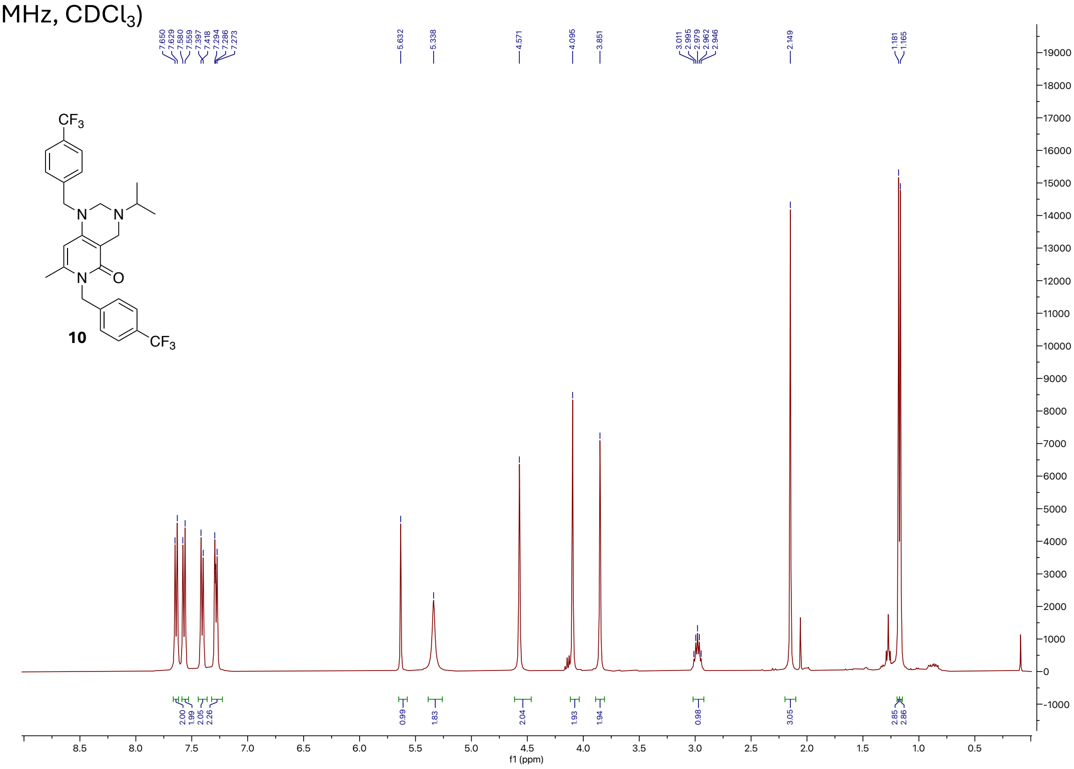

^{13}C NMR (100.6 MHz, $CDCl_3$)

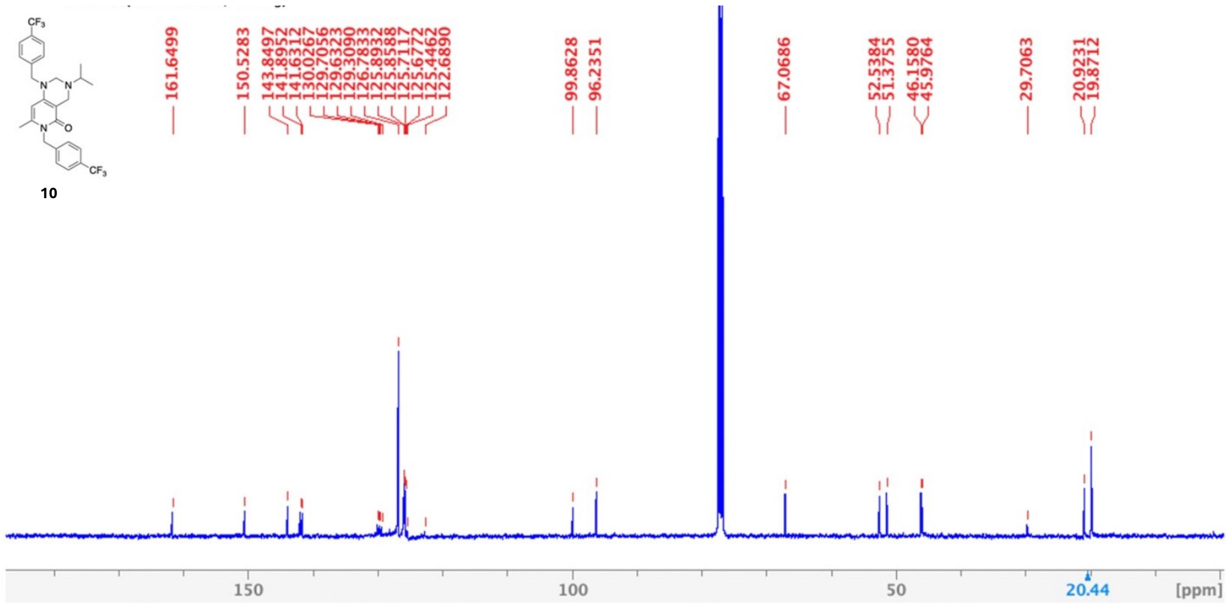

^{19}F NMR (564 MHz, $CDCl_3$)

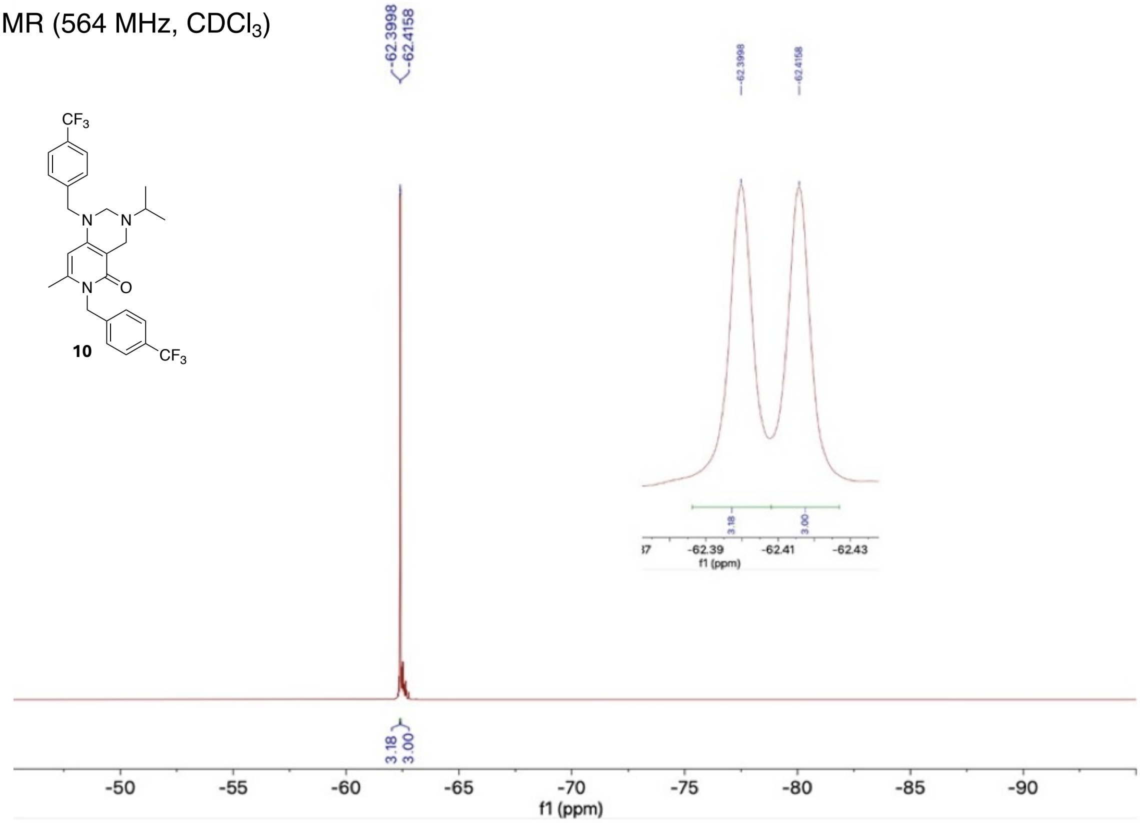

^{1}H NMR (400 MHz, $CDCl_3$)

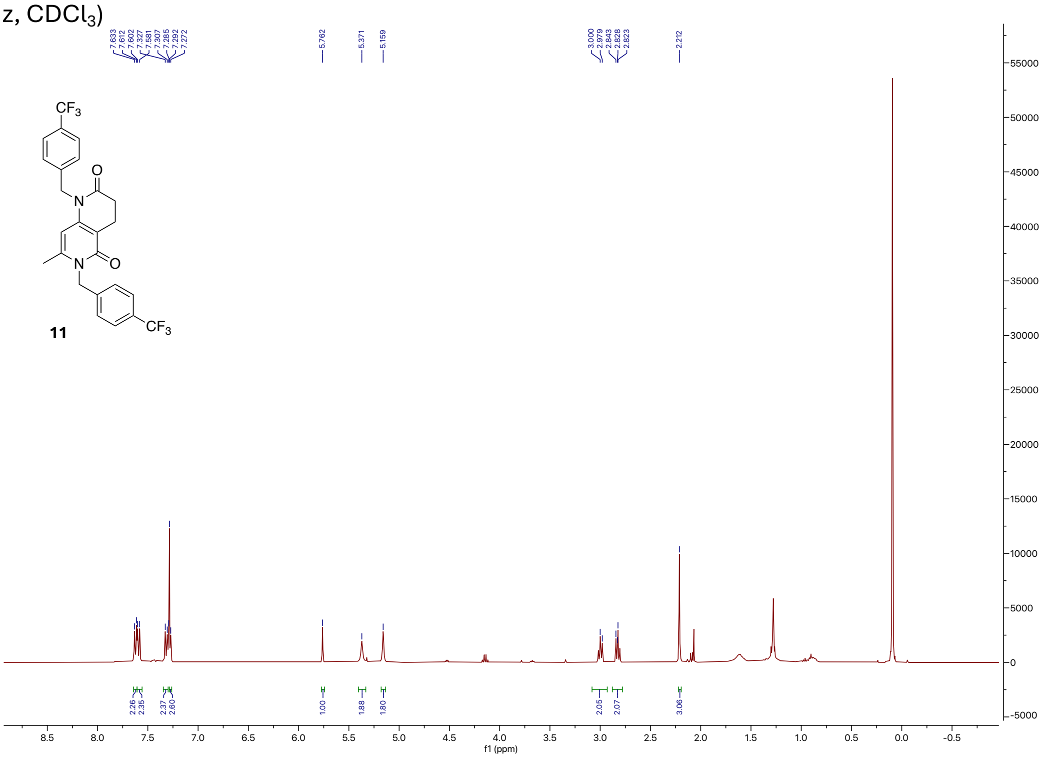

^{13}C NMR (100.6 MHz, $CDCl_3$)

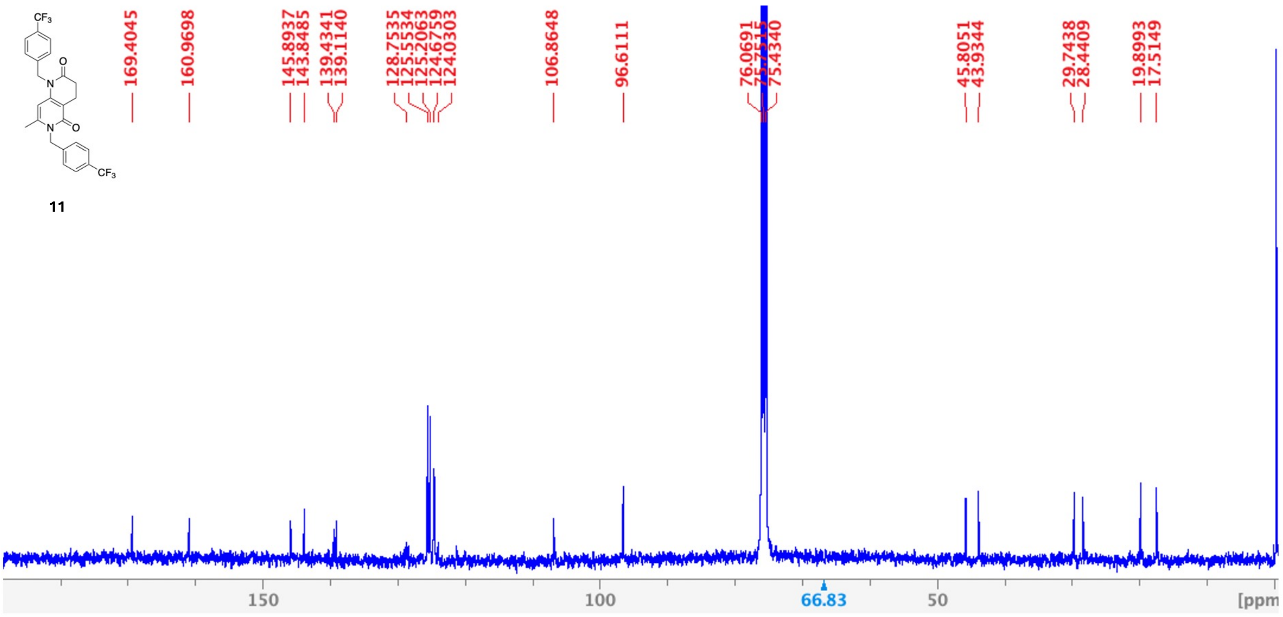

^{19}F NMR (564 MHz, $CDCl_3$)

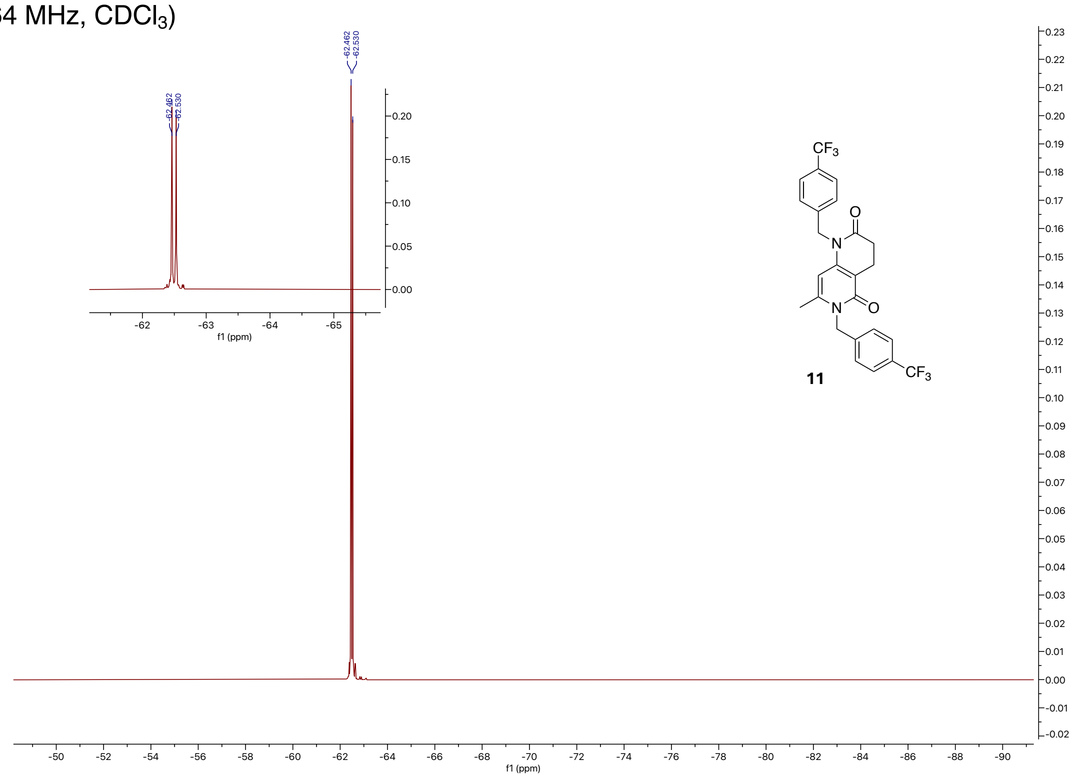

11

^{1}H NMR (400 MHz, $CDCl_3$)

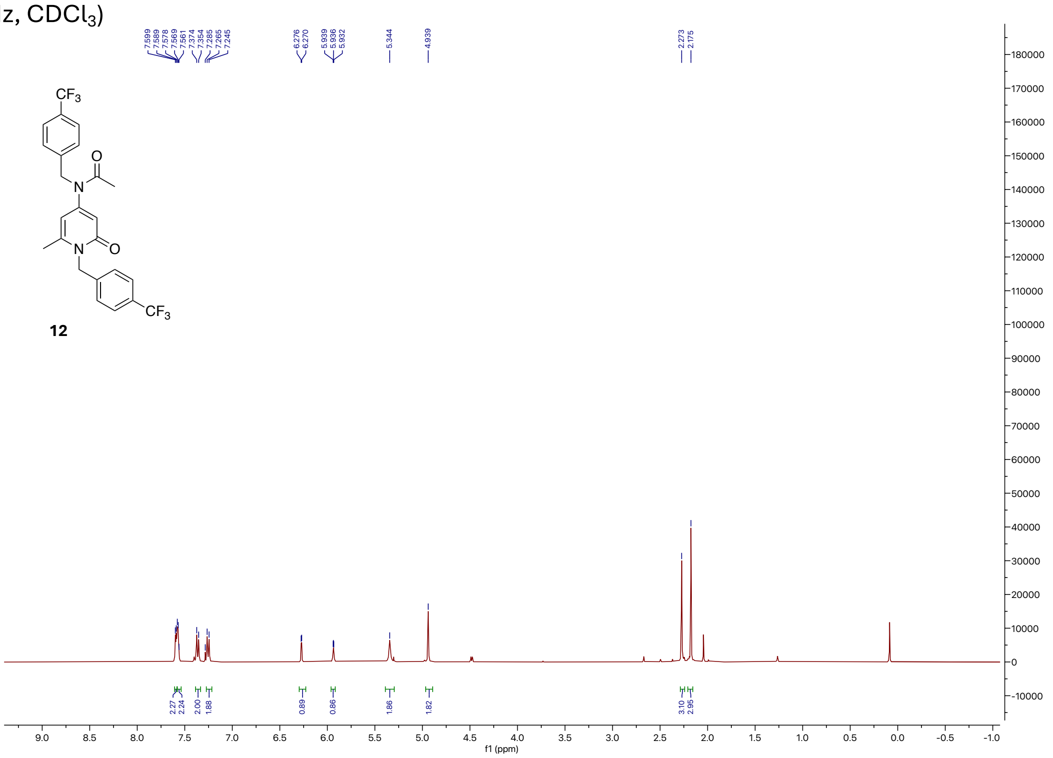

12

^{13}C NMR (100.6 MHz, $CDCl_3$)

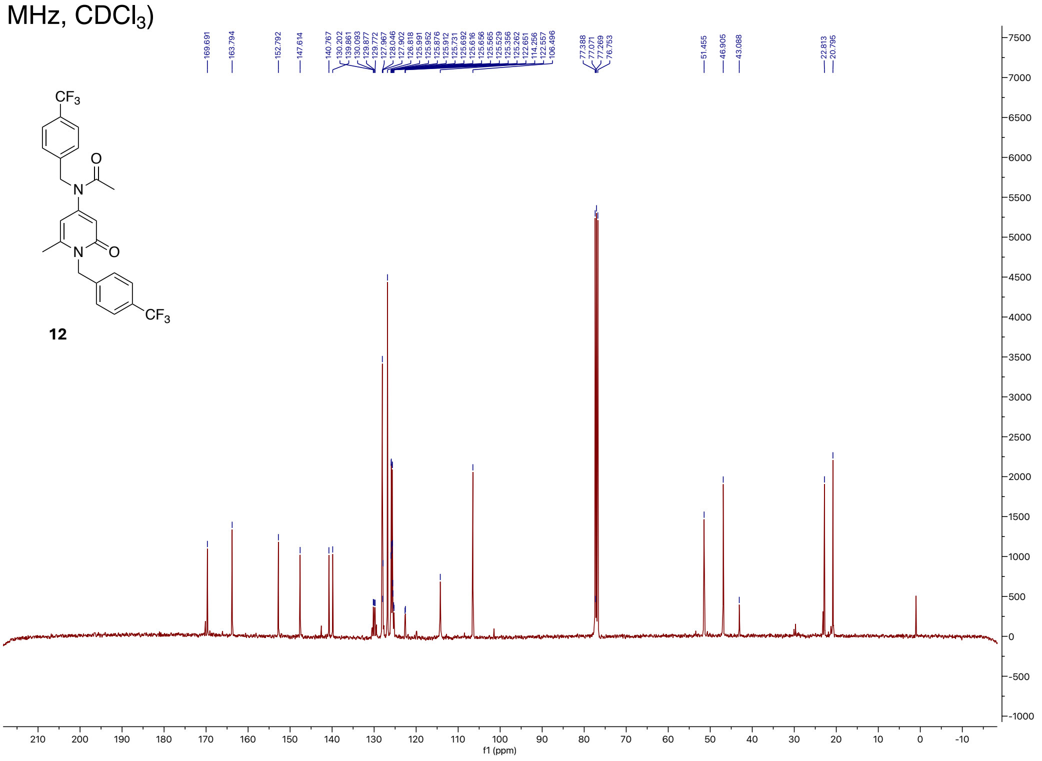

^{19}F NMR (564 MHz, $CDCl_3$)

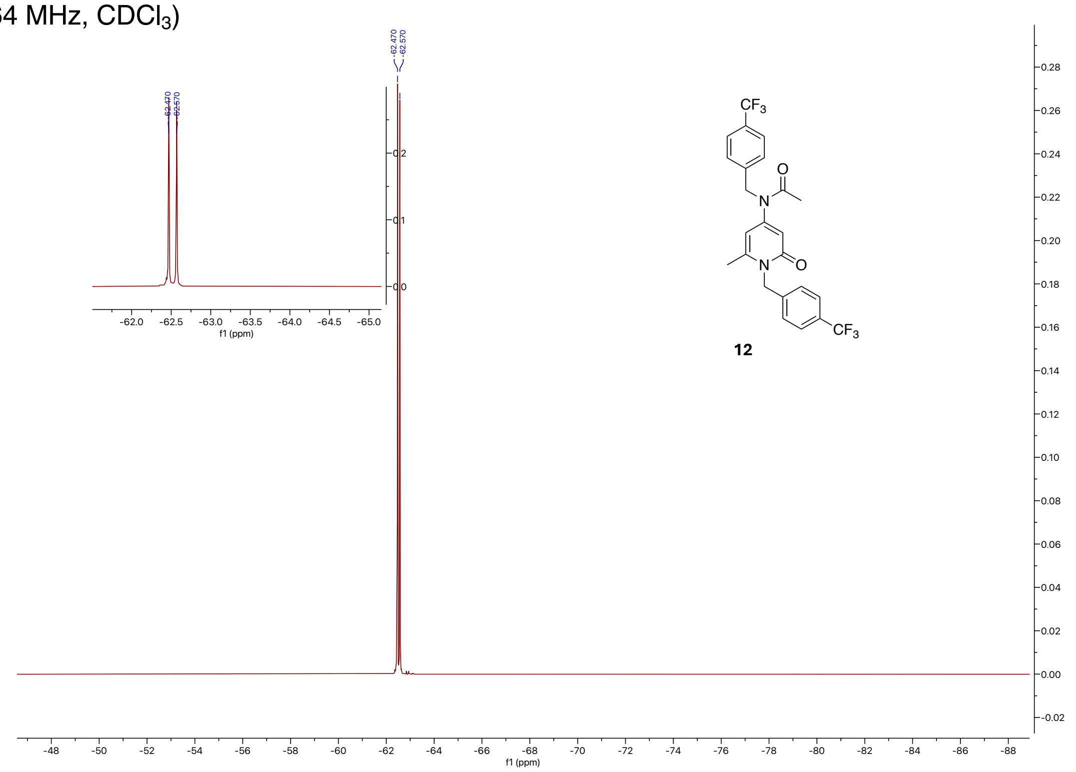

^{1}H NMR (400 MHz, $CDCl_3$)

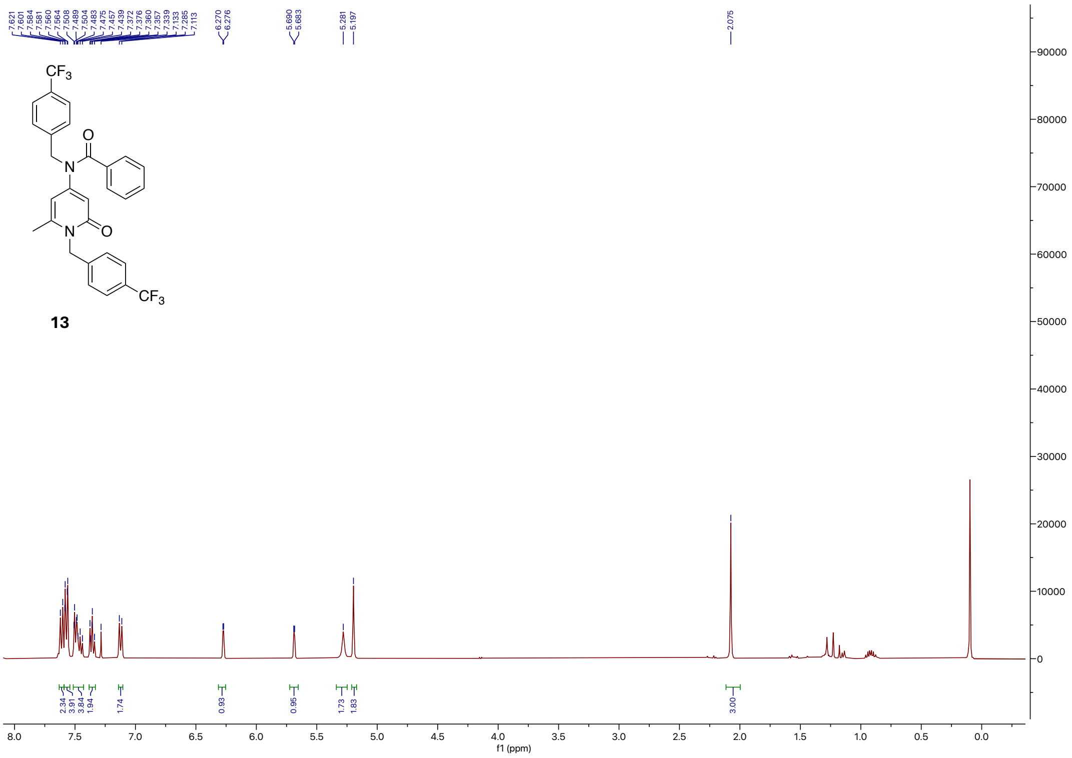

^{13}C NMR (100.6 MHz, $CDCl_3$)

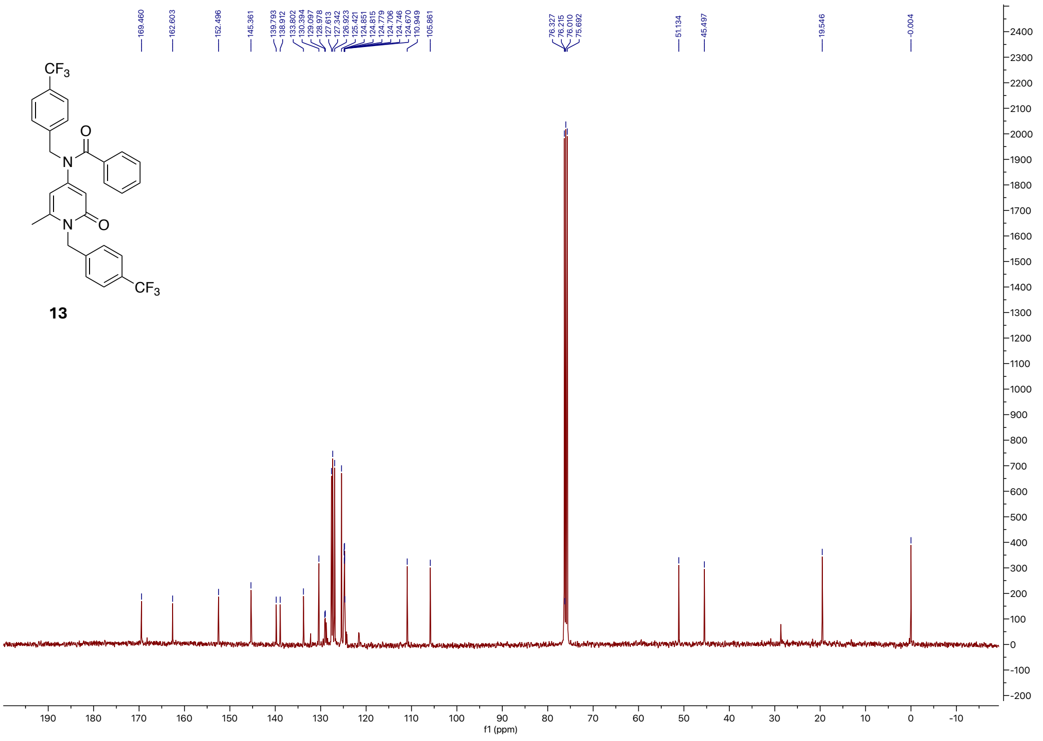

^{19}F NMR (564 MHz, $CDCl_3$)

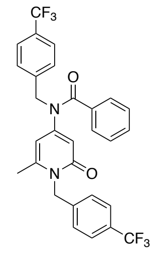

13

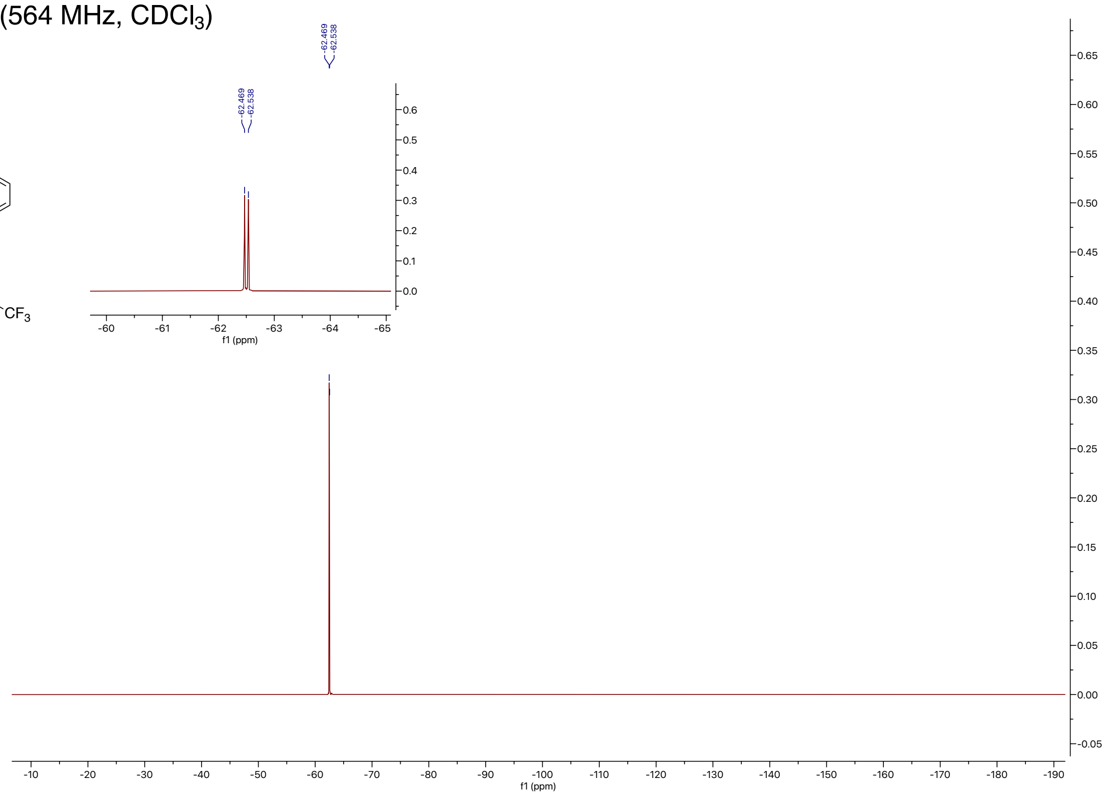

^{1}H NMR (400 MHz, $CDCl_3$)

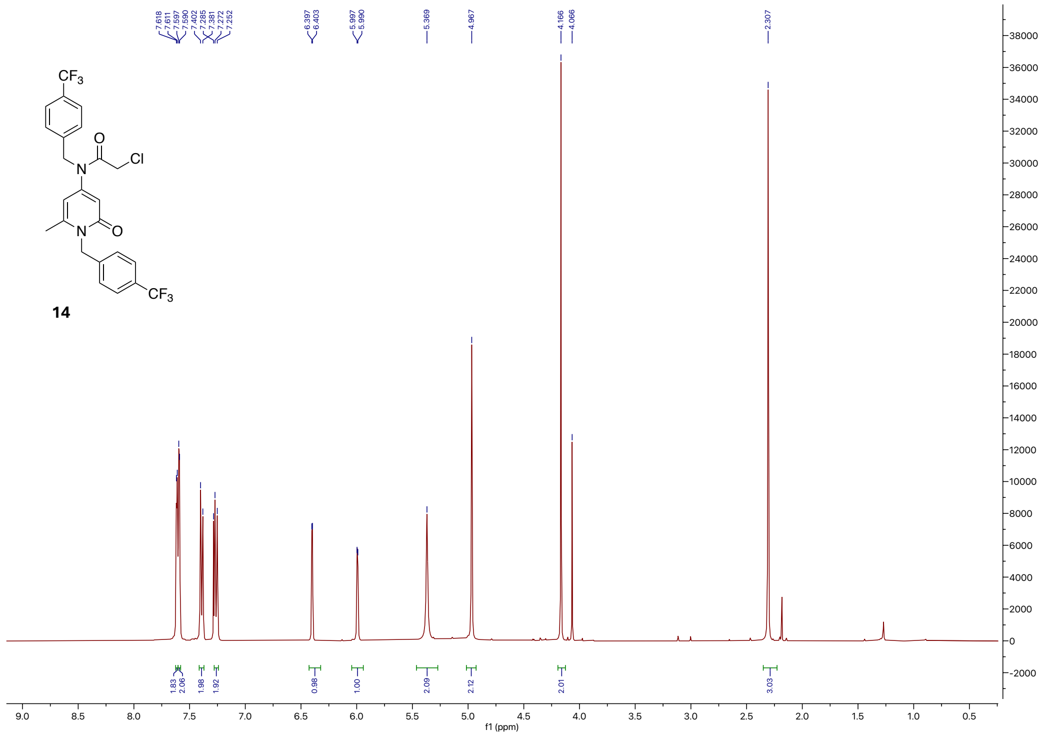

^{13}C NMR (100.6 MHz, $CDCl_3$)

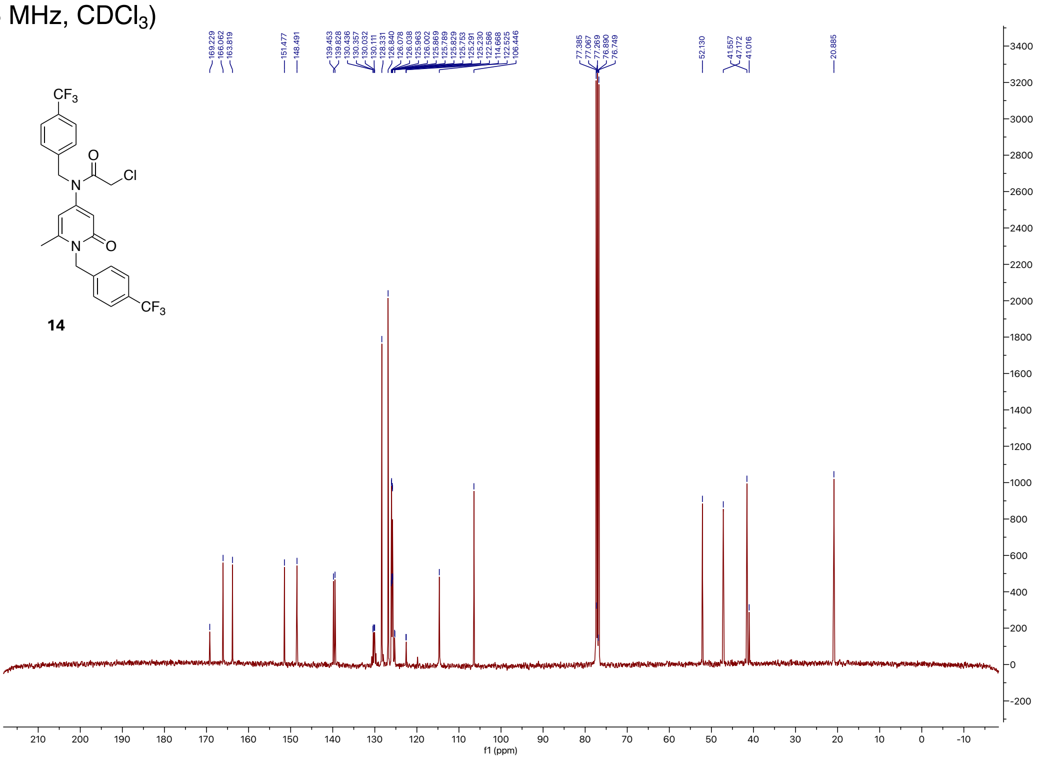

14

^{19}F NMR (564 MHz, $CDCl_3$)

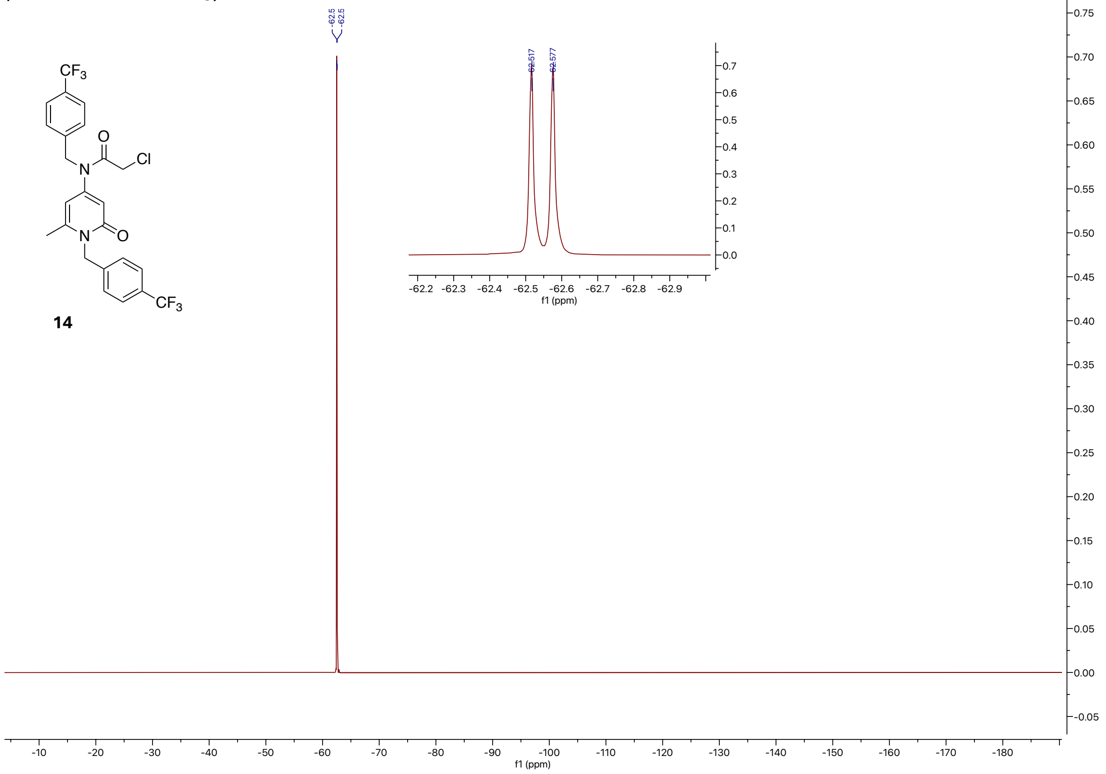

14

^{1}H NMR (400 MHz, $CDCl_3$)

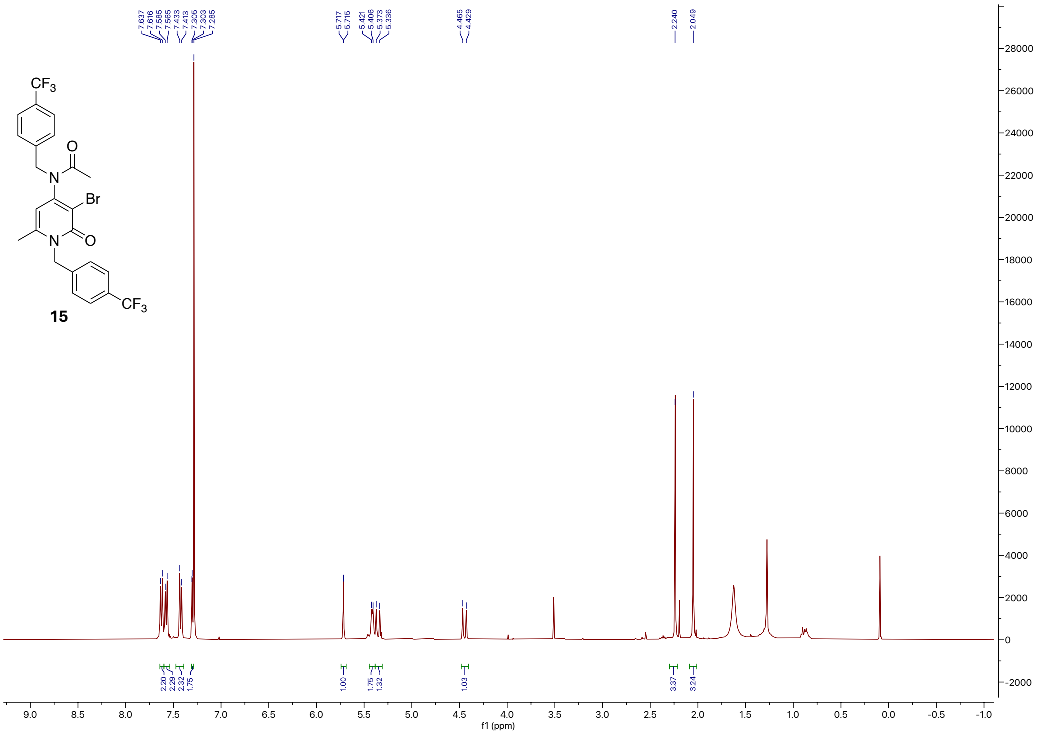

^{13}C NMR (100.6 MHz, $CDCl_3$)

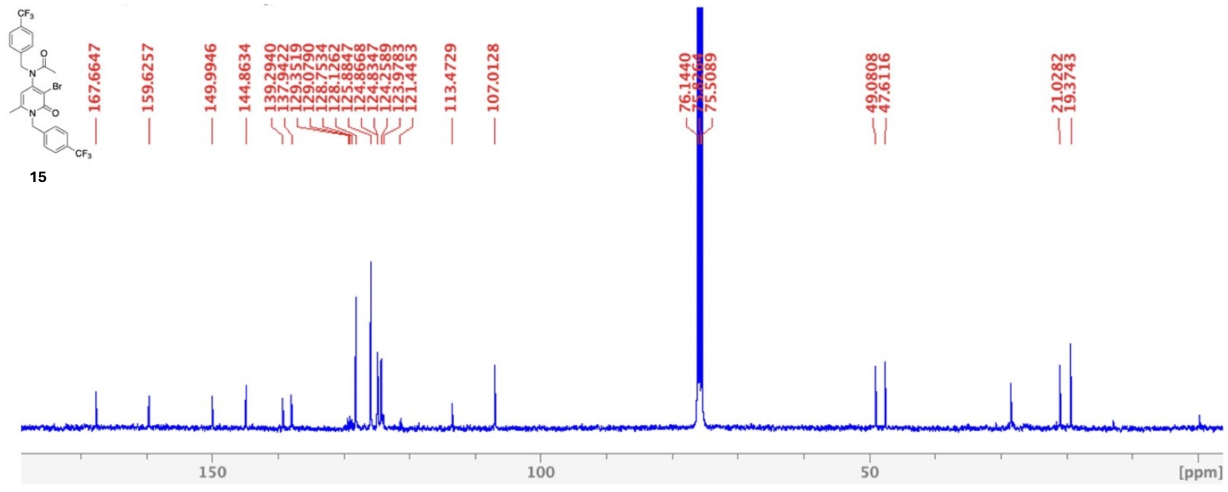

^{19}F NMR (564 MHz, $CDCl_3$)

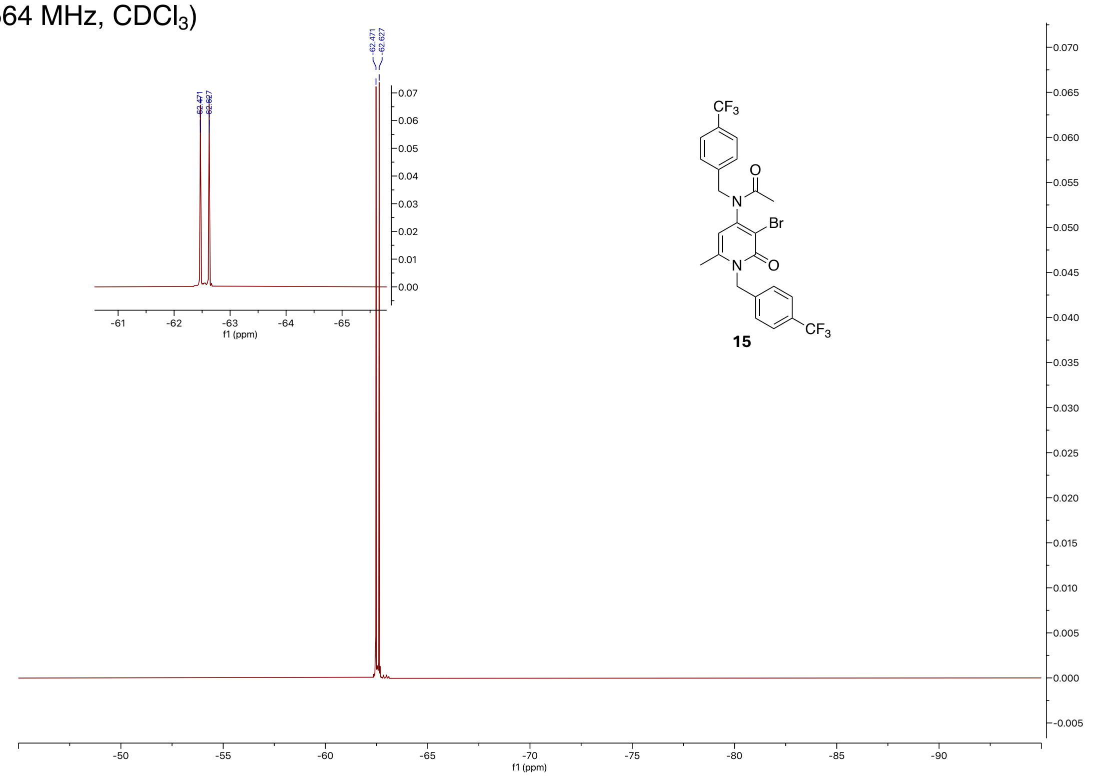

^{1}H NMR (400 MHz, $CDCl_3$)

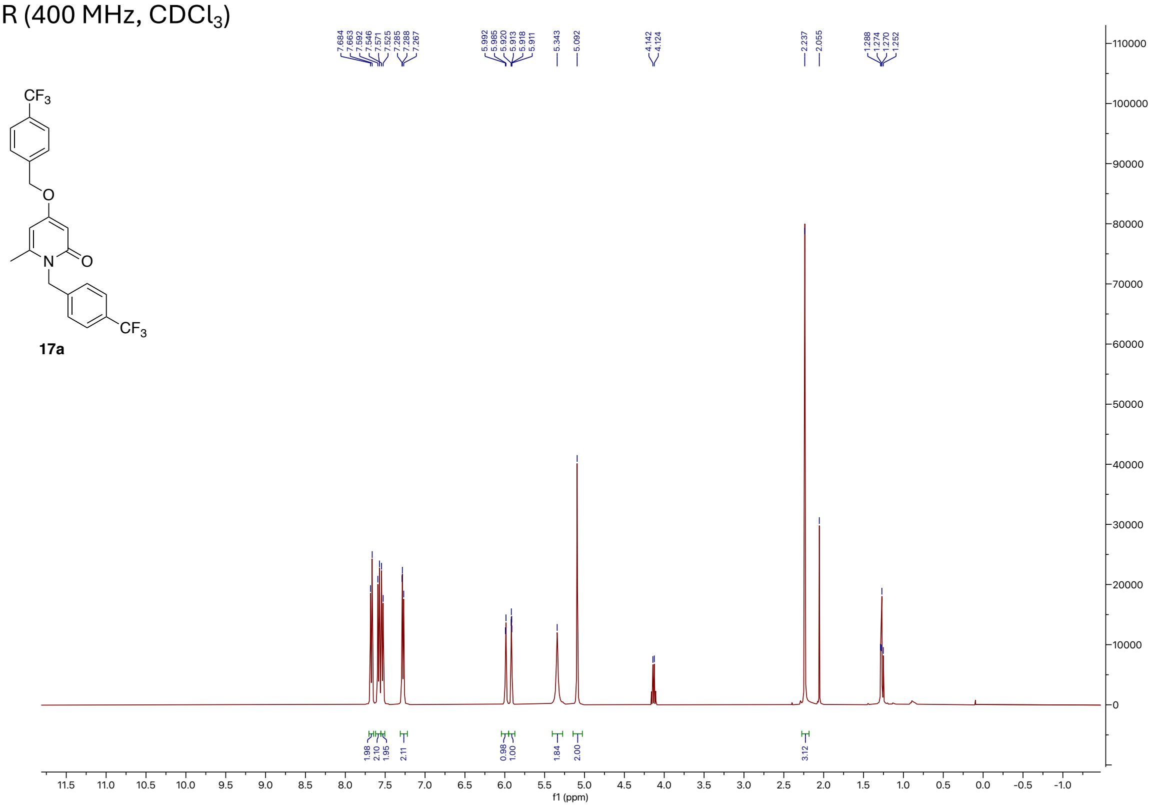

^{13}C NMR (100.6MHz, $CDCl_3$)

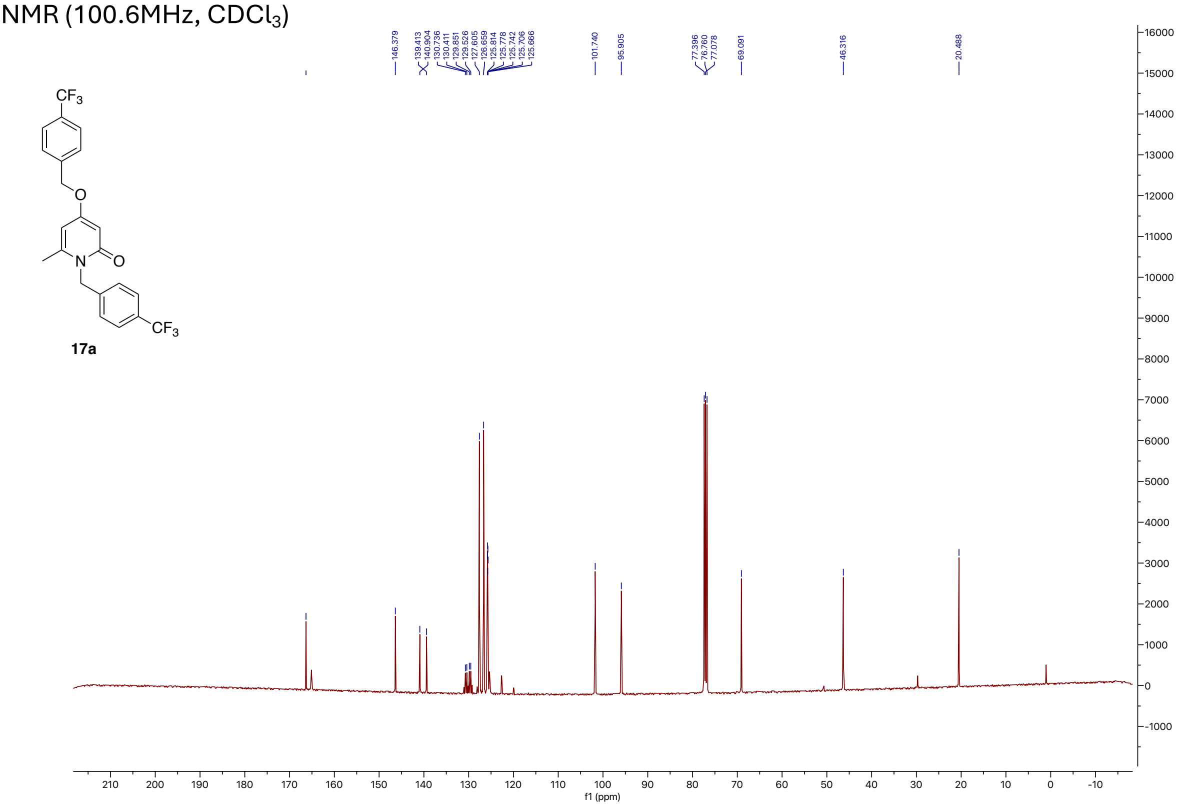

^{19}F NMR (564 MHz, $CDCl_3$)

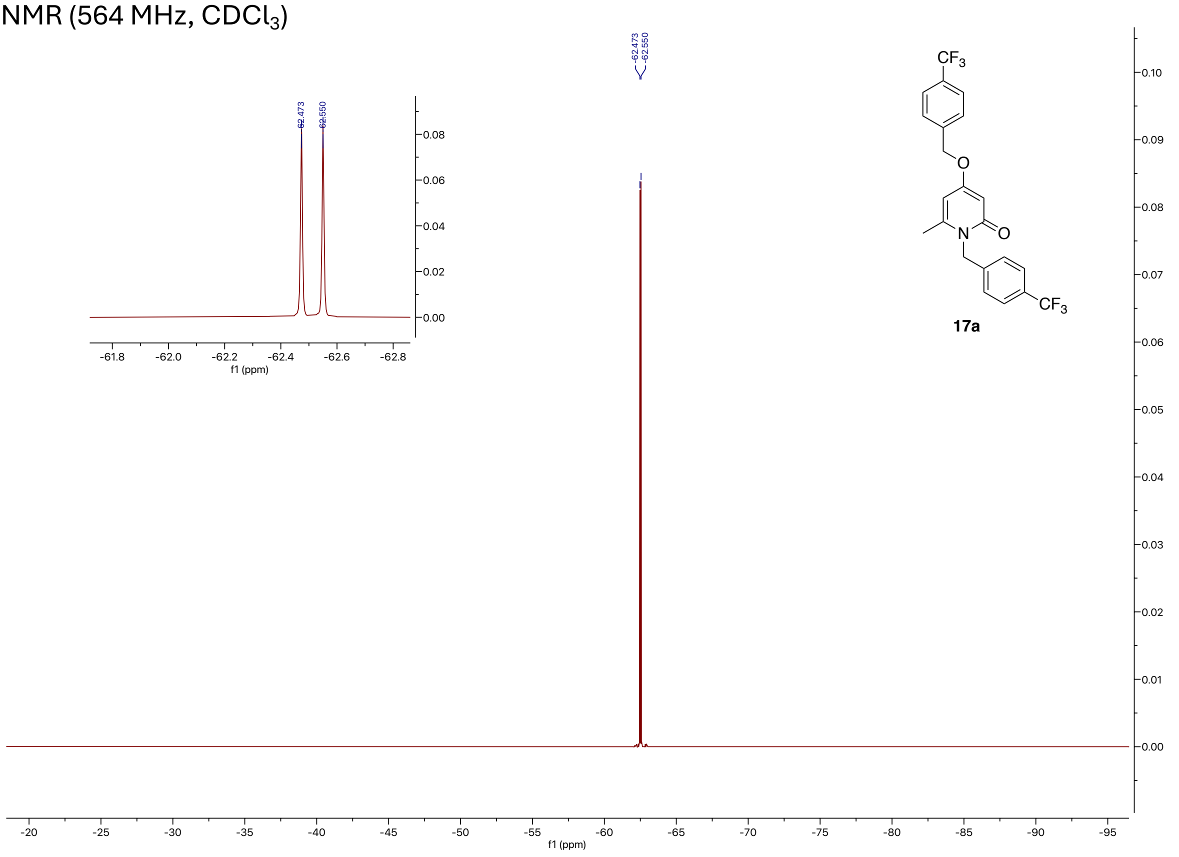

^{1}H NMR (400 MHz, $CDCl_3$)

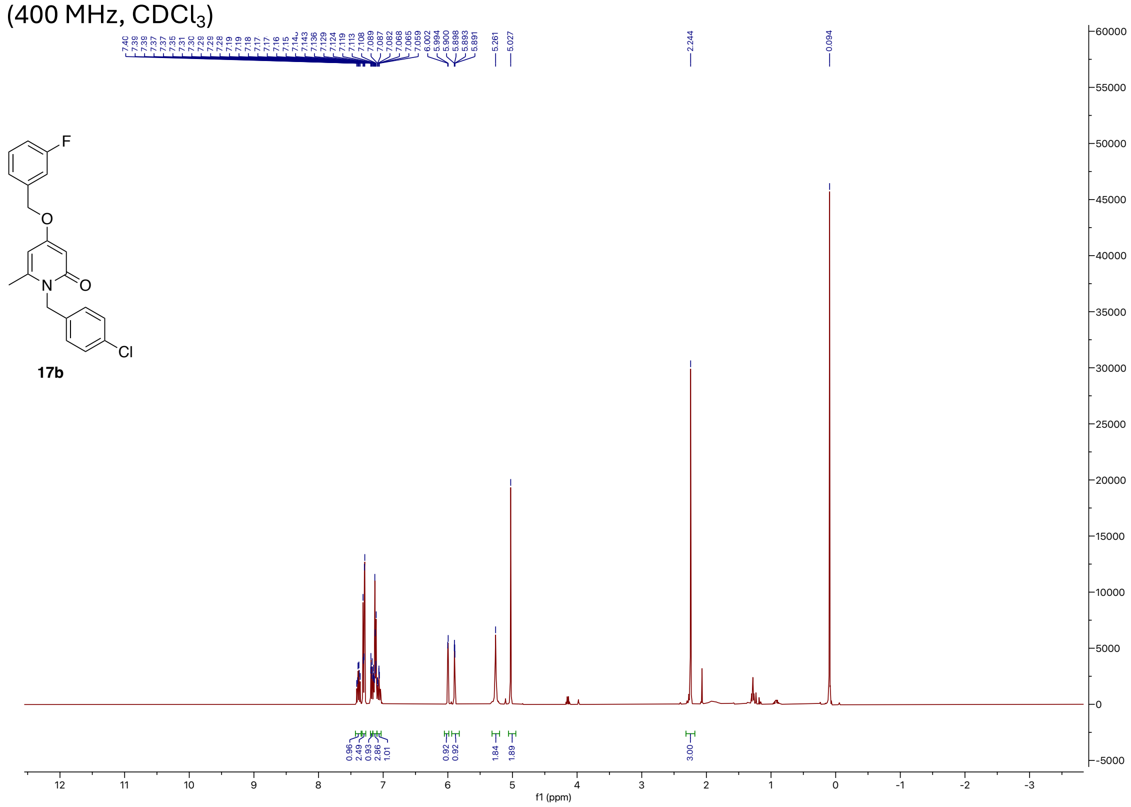

^{13}C NMR (100.6 MHz, $CDCl_3$)

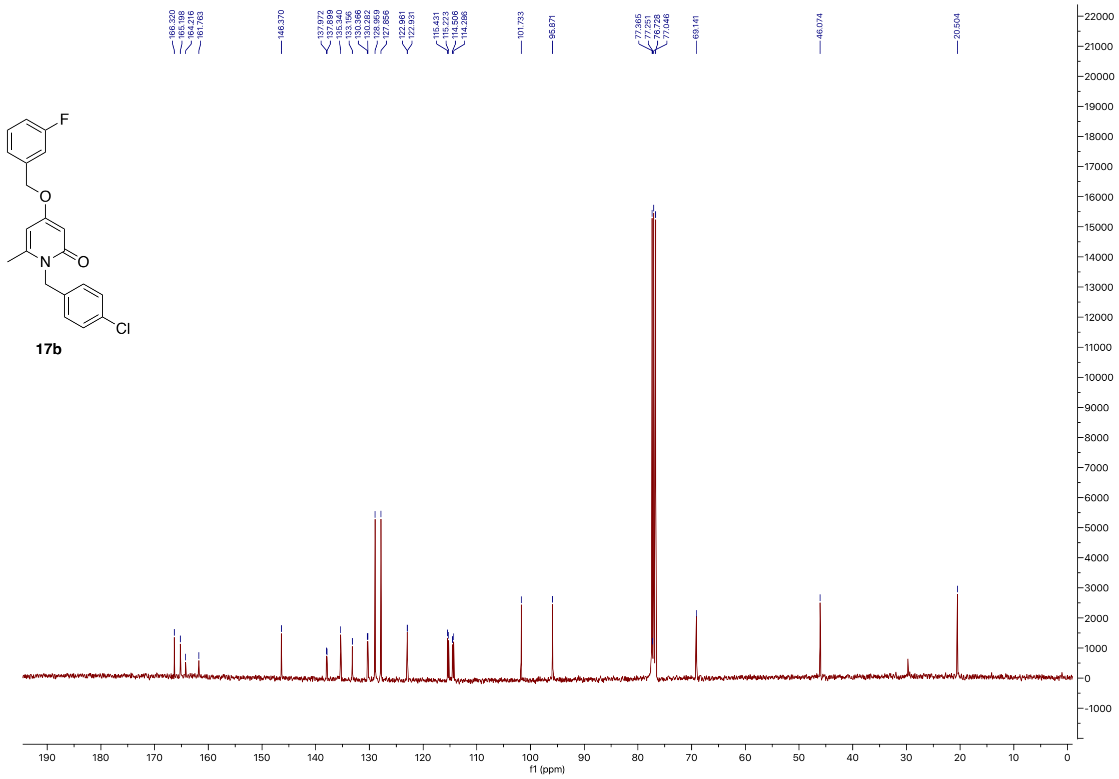

^{19}F NMR (564 MHz, $CDCl_3$)

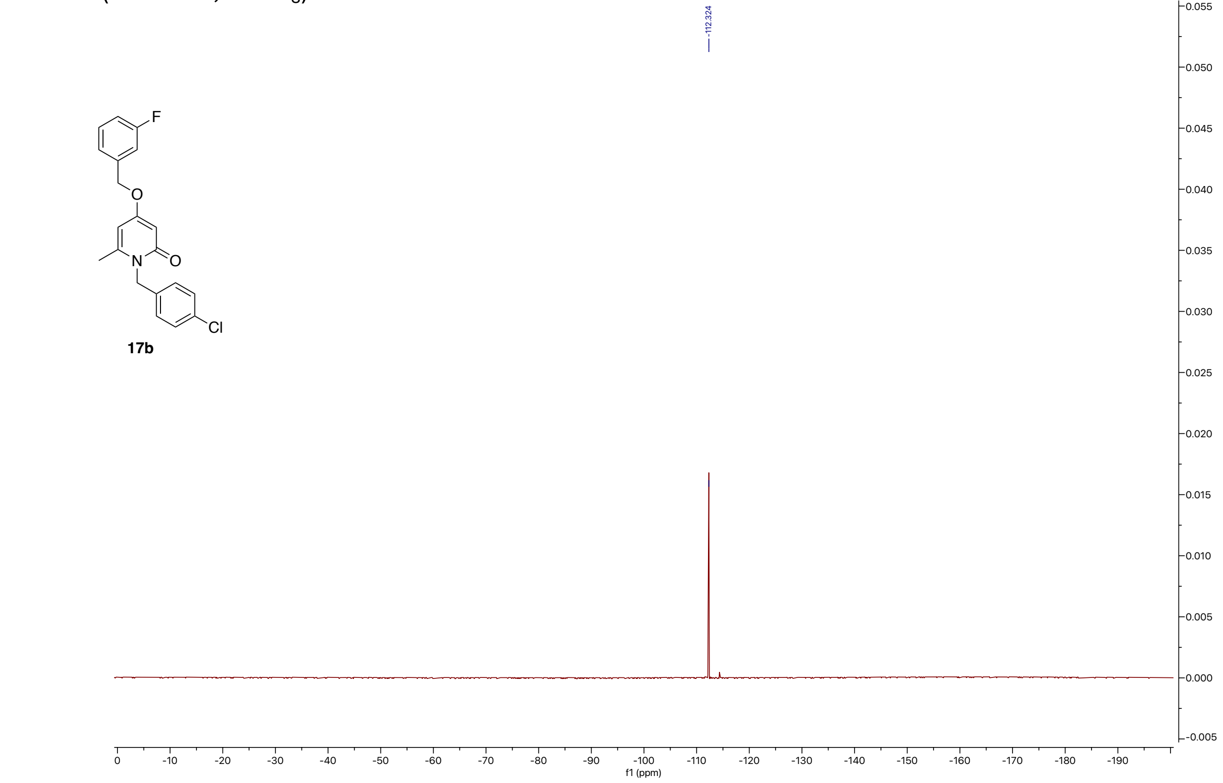

^{1}H NMR (400 MHz, $CDCl_3$)

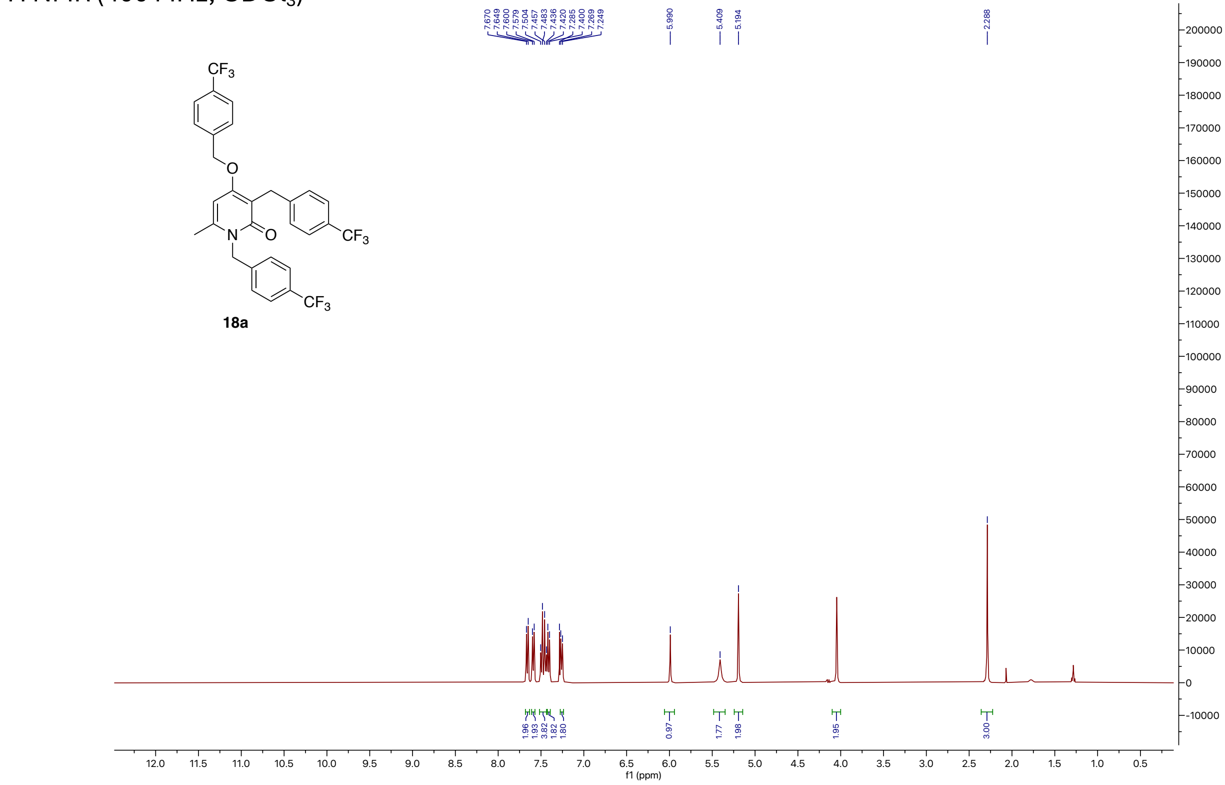

^{13}C NMR (100.6MHz, $CDCl_3$)

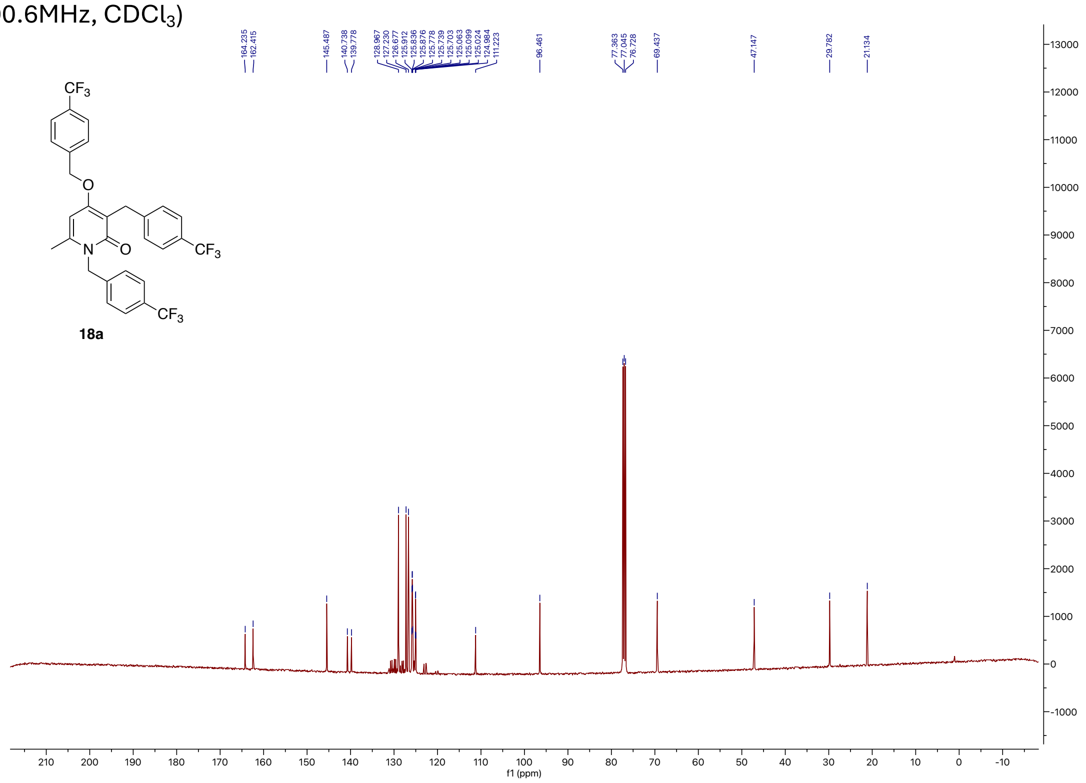

^{19}F NMR (564 MHz, $CDCl_3$)

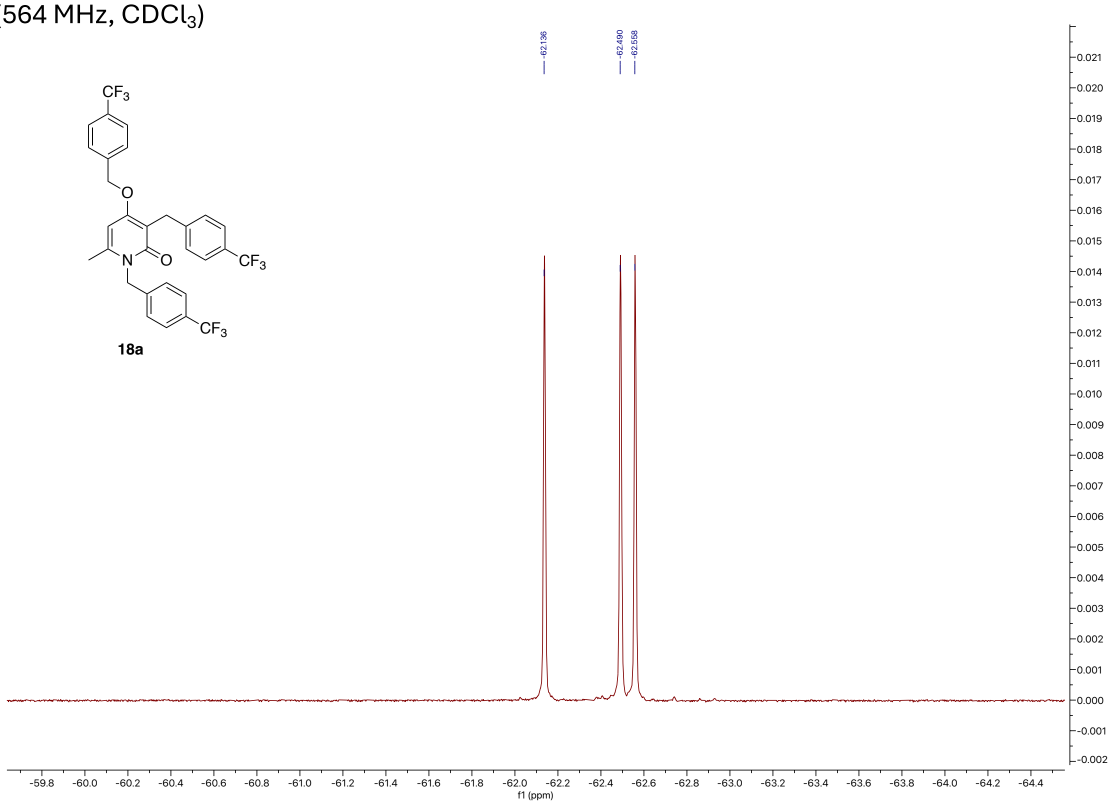

^{1}H NMR (400 MHz, $CDCl_3$)

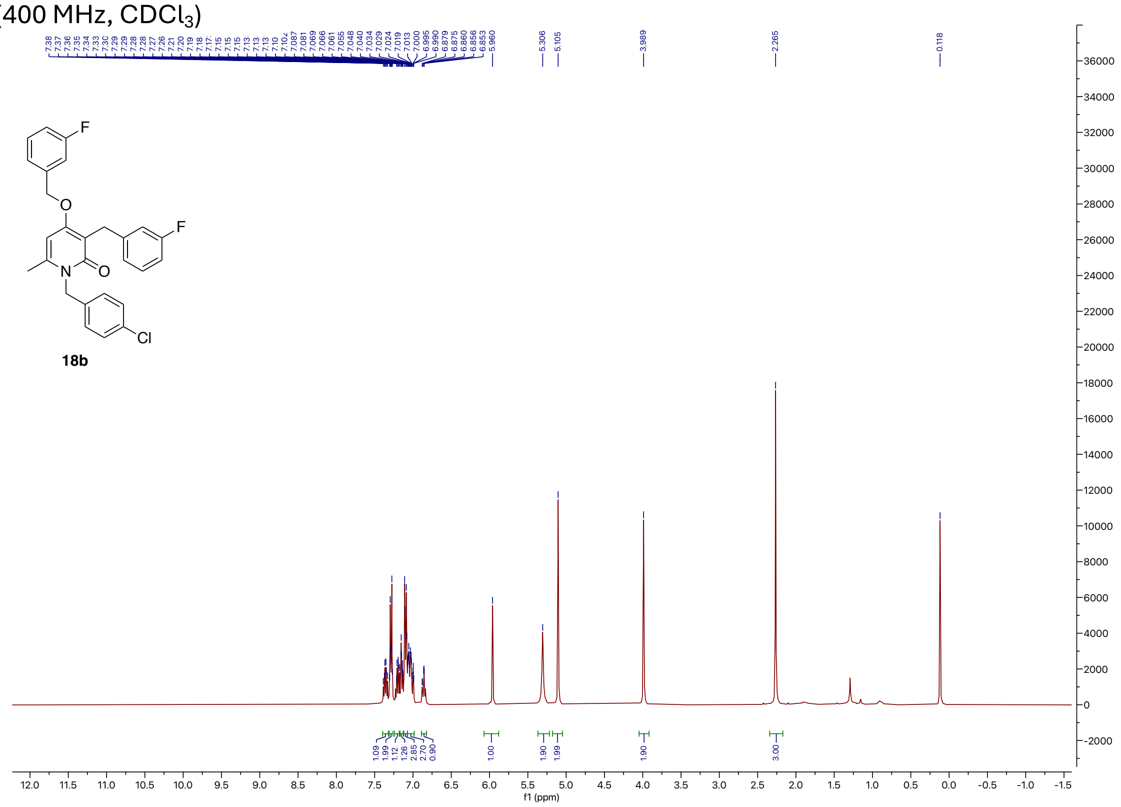

^{13}C NMR (100.6MHz, $CDCl_3$)

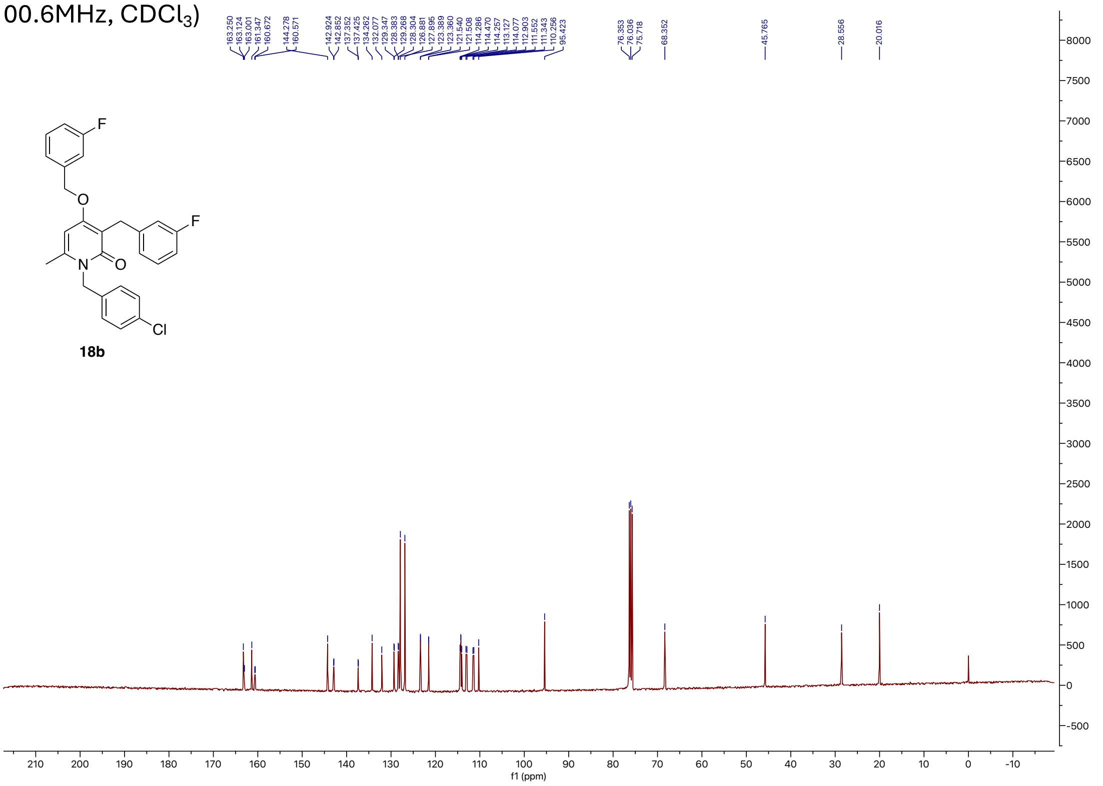

^{19}F NMR (564 MHz, $CDCl_3$)

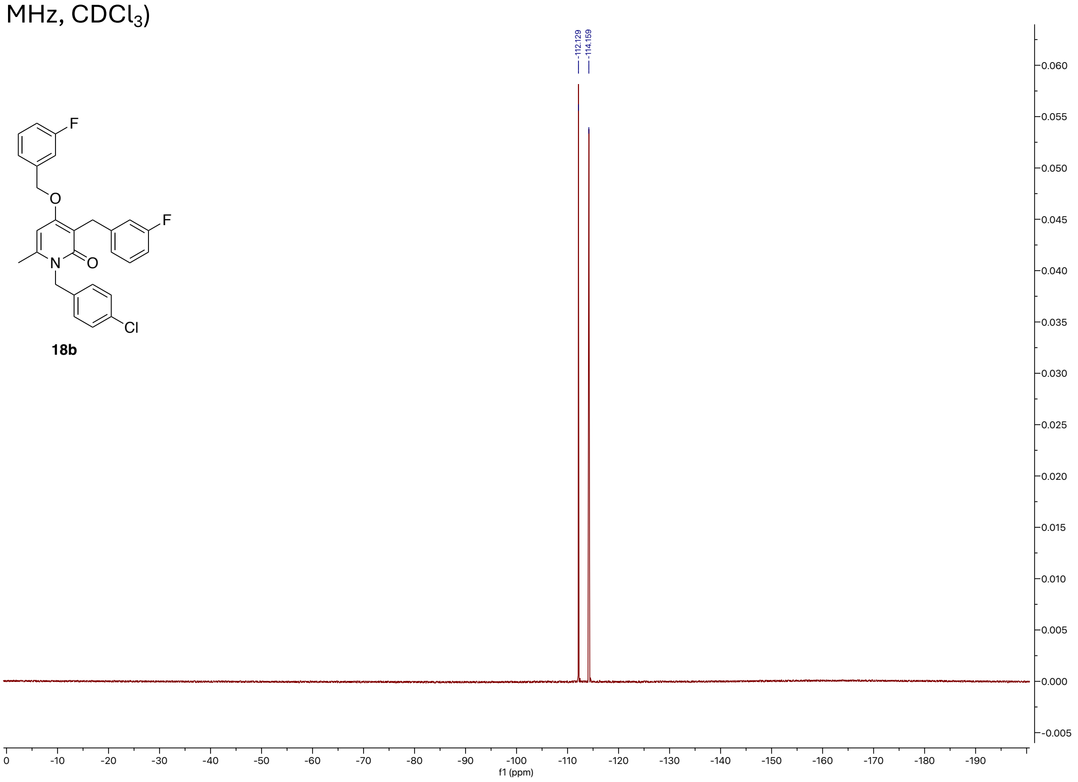

Supplement: Supplementary file 1 — Supplementary Material [file CMDC-21-e202500651-s001.pdf]
